# Selective Catalytic Frustrated Lewis Pair Hydrogenation of CO_2_ in the Presence of Silylhalides

**DOI:** 10.1002/anie.202112233

**Published:** 2021-11-03

**Authors:** Tongtong Wang, Maotong Xu, Andrew R. Jupp, Zheng‐Wang Qu, Stefan Grimme, Douglas W. Stephan

**Affiliations:** ^1^ Department of Chemistry University of Toronto 80 St. George St. Toronto Ontario M5S3H6 Canada; ^2^ School of Chemistry, Faculty of Chemical Environmental and Biological Science and Technology Dalian University of Technology China; ^3^ Mulliken Center for Theoretical Chemistry University of Bonn Beringstr. 4 53115 Bonn Germany

**Keywords:** acetal, CO_2_ hydrogenation catalysis, frustrated Lewis pair, methane, methyliodide

## Abstract

The frustrated Lewis pair (FLP) derived from 2,6‐lutidine and B(C_6_F_5_)_3_ is shown to mediate the catalytic hydrogenation of CO_2_ using H_2_ as the reductant and a silylhalide as an oxophile. The nature of the products can be controlled with the judicious selection of the silylhalide and the solvent. In this fashion, this metal‐free catalysis affords avenues to the selective formation of the disilylacetal (R_3_SiOCH_2_OSiR_3_), methoxysilane (R_3_SiOCH_3_), methyliodide (CH_3_I) and methane (CH_4_) under mild conditions. DFT studies illuminate the complexities of the mechanism and account for the observed selectivity.

## Graphical abstract

The frustrated Lewis pair (FLP), 2,6‐lutidine/ B(C_6_F_5_)_3_ mediates the catalytic reduction of CO_2_ using H_2_ and a silylhalide. Control of the silylhalide and solvent, affords selective avenues to the disilylacetal (R_3_SiOCH_2_OSiR_3_), methoxysilane (R_3_SiOCH_3_), methyliodide (CH_3_I) and methane (CH_4_) under mild conditions. The mechanism is studied by DFT and accounts for the observed selectivity.

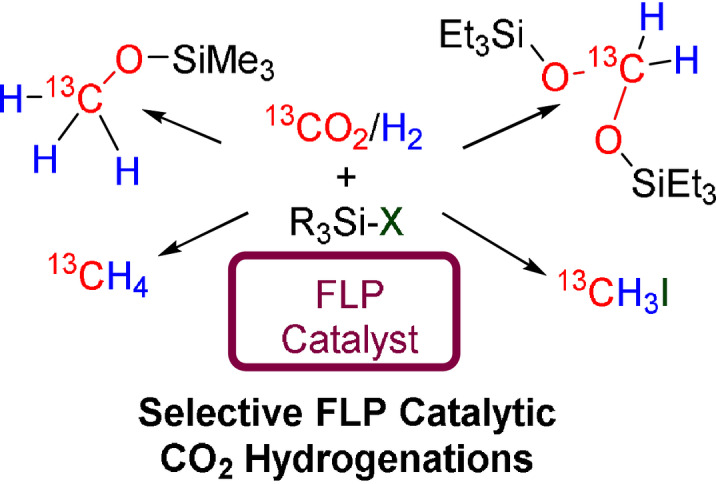

The dramatic and continuous increase in the atmospheric CO_2_ level since the industrial revolution results from the extensive use of fossil fuels and is the major contributor to climate change. This has prompted the scientific community to target a variety of new technologies to reduce emissions or provide alternative energy sources as these offer the most promising avenues to address climate change. Nonetheless, other efforts targeting the capture or use of atmospheric CO_2_ have also garnered attention. One potential avenue to the use of atmospheric CO_2_ involves reduction via hydrogenation.^[1]^ For example, recent reviews have described the conversion of CO_2_ to methanol using homogeneous and heterogeneous transition metal‐based catalysts^[2]^ while other reports have demonstrated the production of longer chain fuels^[3]^ or olefins or higher alcohols.^[4]^ In addition to the above metal‐catalyzed processes, there have also been extensive efforts to employ main group reagents to mediate CO_2_ reduction processes. A number of studies^[5]^ have explored catalytic processes including both base‐mediated and frustrated Lewis pair (FLP) hydrosilylations^[6]^ and hydroborations^[7]^ of CO_2_ while others have probed aminations.^[8]^

Despite the seminal finding in 2009 in which Ashley and O'Hare^[9]^ reported the FLP‐mediated reduction of CO_2_ to methanol (Scheme 1), albeit in low yield and at 160 °C for 6 days, the direct hydrogenation of CO_2_ mediated by a main group species has garnered limited attention. A collaborative effort with the Fontaine group^[10]^ described the stoichiometric reactions of the intramolecular FLP, 1‐BMes_2_‐2‐NMe_2_‐C_6_H_4_, with H_2_ and CO_2_ yielding formyl, acetal and methoxy‐borane derivatives (Scheme 1). This study suggested that judicious selection of the combination of the Lewis acid and the base could plausibly lead to catalytic H_2_/CO_2_ chemistry. More recently, Zhao et al.^[11]^ described the hydrogenation of CO_2_ in the presence of H_2_ and K_2_CO_3_ using B(C_6_F_5_)_3_ as the catalyst, affording effective turn‐over to K[HCO_2_] at comparatively high H_2_/CO_2_ pressures of 60 bar (Scheme 1). While the achievement of catalytic hydrogenation is impressive, the reduction was limited to the formation of formate product.

**Scheme 1:**
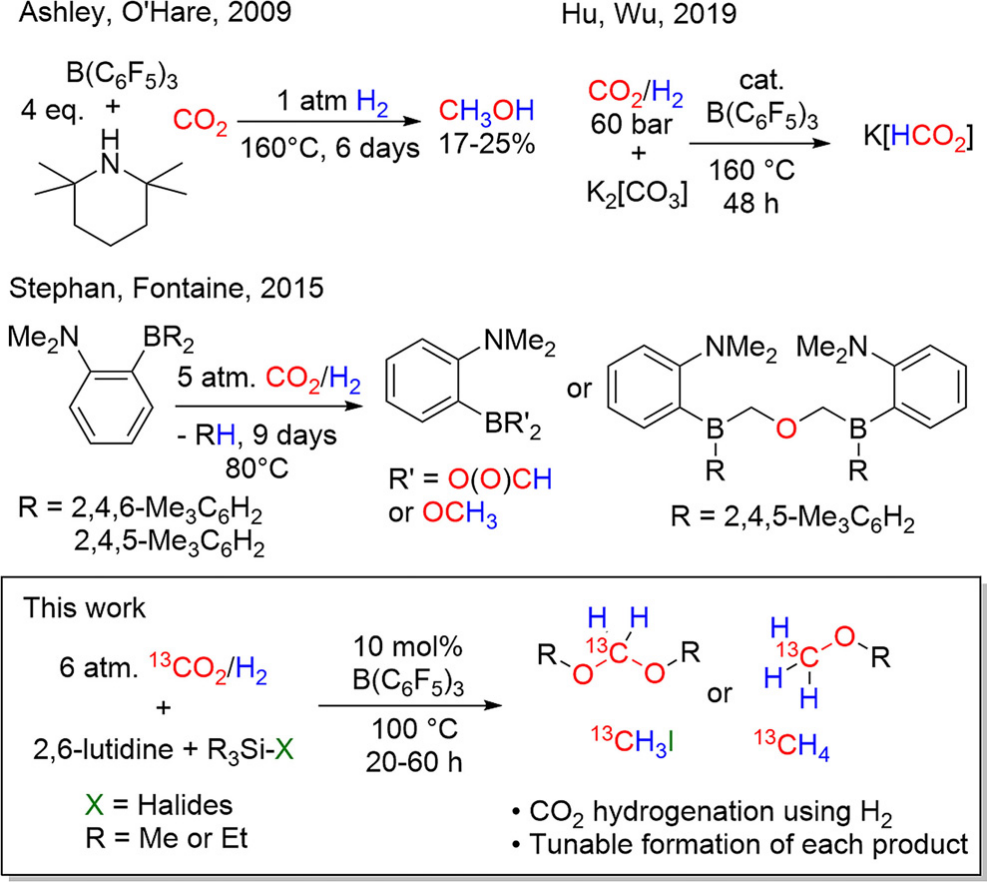
Direct reactions of CO_2_/H_2_ mediated by main group reagents.

Pondering an FLP system that would effect reduction beyond formate, we recognized that in earlier studies methanol or methane were obtained exploited hydrosilanes or hydroboranes that provide both a reducing agent and an oxophile.[^6^, ^7^] In contrast, use of H_2_ as the reducing agent in direct FLP hydrogenations of CO_2_ does not provide such an oxygen‐atom scavenger. Thus, we speculated that further hydrogenation of CO_2_ could be effected in the presence of a silylhalide. Herein, we report the FLP‐mediated catalytic hydrogenation of CO_2_ using H_2_ as the reducing agent performed in the presence of a silylhalide which acts as an oxophile. Judicious choices of the silylhalide and reaction solvent are shown to provide fine control over the nature of the products of catalysis.

The activation of H_2_ by 2,6‐lutidine/B(C_6_F_5_)_3_ (Scheme 2)^[12]^ and subsequent reaction with CO_2_ is known to afford the salt [C_5_H_3_Me_2_NH][HCO_2_B(C_6_F_5_)_3_].^[13]^ This species was allowed to react with 1 equivalent of Et_3_SiI in CDCl_3_ resulting in the upfield shift of the formyl proton in the ^1^H NMR from 8.31 ppm to 8.17 ppm and the appearance of a ^11^B{^1^H} NMR signal at −0.1 ppm. These data affirm the formation of B(C_6_F_5_)_3_ adduct of silyl formate Et_3_SiOC(O)H^[6c]^ and are consistent with the cleavage of the B−O bond in the formyl‐borate salt (Scheme 2). Recognizing that the silyl formate‐borane adduct will exist in an equilibrium with free borane, this implies that it should be accessible for further reaction.

**Scheme 2:**
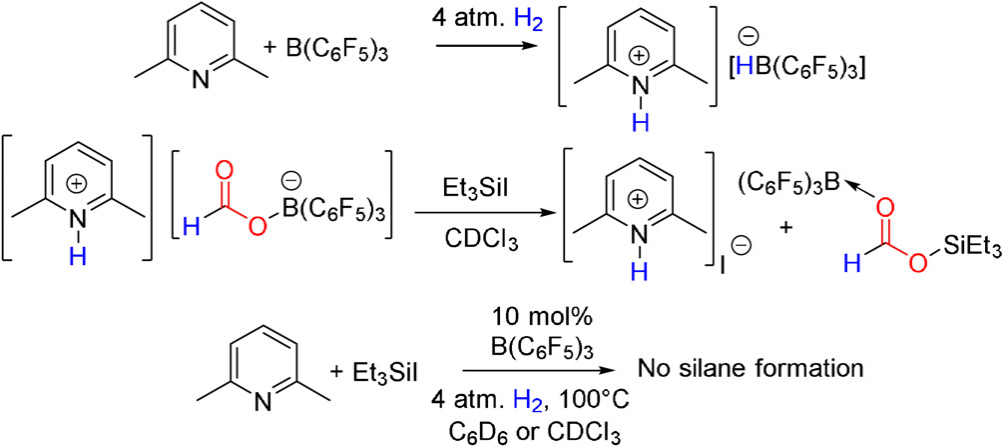
Control reactions.

We also queried the possibility of reduction of Et_3_SiI in the presence of excess base. To this end, Et_3_SiI and 2,6‐lutidine were combined under H_2_ (4 atm) in the presence of 10 mol % B(C_6_F_5_)_3_ in either CDCl_3_ or C_6_D_6_ and heated at 100 °C for 40 h (Scheme 2). In both cases no reduction of the silylhalide was observed. This suggested that the silylhalide could act as an oxophile in the presence of H_2_, for the hydrogenation of CO_2_, without the possibility of invoking a hydrosilylation mechanism.

Thus, targeting FLP hydrogenations of CO_2_, reactions of 10 equivalents of Lewis base and silylhalide were performed in C_6_D_6_ or CDCl_3_ solution of 10 mol % of B(C_6_F_5_)_3_. In these reactions the substituted pyridines, 2,4,6‐collidine and less basic 2,6‐lutidine were employed and the systems were pressurized with H_2_ (4 atm.) and ^13^CO_2_ (2 atm.) and heated to 100 °C for up to 60 h. The reactions were monitored by ^1^H NMR and ^13^C NMR spectroscopy. Initial reactions using Me_3_SiCl and 2,6‐lutidine in C_6_D_6_ or CDCl_3_ (Table 1, entry 1, 2) as the solvent, afforded [C_5_H_3_Me_2_NH][HCO_2_B(C_6_F_5_)_3_]^[13]^ as the major product as evidenced by the doublet resonance (^1^ *J*_C–H_=209 Hz) at 8.37 ppm in the ^1^H NMR spectrum and the doublet resonance in the ^1^H‐coupled ^13^C NMR at 169.5 ppm. The generally poor reactivity in the presence of Me_3_SiCl was attributed to the relatively strong Si−Cl bond and prompted the use of 2,6‐lutidine and Me_3_SiBr. This led to an 83 % yield of methoxysilane Me_3_SiO^13^CH_3,_ after 40 h of heating in C_6_D_6_ (entry 3). In this case, the major product was identified by a ^1^H NMR resonance at 3.25 ppm as a doublet (^1^ *J*_C–H_=141 Hz), the corresponding ^13^C{^1^H} NMR signal is found at 49.9 ppm.^[6b]^ Repetition of the experiment in CDCl_3_ also led to the selective production of Me_3_SiO^13^CH_3_ in 73 % yield after 60 h heating (entry 4). The combination of 2,6‐lutidine and Me_3_SiI generated ^13^CH_4_ in 76 % yield after 60 h (entry 5). As these reactions were done in a sealed J‐Young NMR tube, the methane was identified by ^13^C NMR spectroscopy as a pentet at −4.3 ppm (^1^ *J*_C–H_=126 Hz) and further confirmed by an HSQC experiment, revealing a correlation with the ^1^H signal at 0.19 ppm.^[14]^ Further improvement in the reactivity was seen with use of CDCl_3_ as the solvent as ^13^CH_4_ was produced in 85 % yield after 20 h at 100 °C (entry 6). Reactions with the more sterically hindered halosilane Et_3_SiI afforded the acetal (Et_3_SiO)_2_^13^CH_2_ as the dominant product in 72 % yield after heating at 100 °C for 60 h (entry 7). This product exhibited a doublet at 5.06 ppm in the ^1^H NMR with a ^1^ *J*_C–H_ of 162 Hz and a ^13^C{^1^H} NMR signal at 84.5 ppm. Interestingly, performance of the reaction in the more polar solvent CDCl_3_ (entry 8) afforded ^13^CH_3_I in 82 % yield as evidenced by the quartet resonance in the ^13^C NMR at −23.5 ppm with ^1^ *J*_C–H_=151 Hz, while the HSQC experiment revealed a correlation with the ^1^H signal at 2.16 ppm.^[15]^ Use of the more basic 2,4,6‐collidine resulted in a significant reduction in reactivity affording low yields of the acetal and methoxylsilane in C_6_D_6_ and CDCl_3_, respectively (entry 9, 10), likely due to slightly reduced reactivity for CO_2_ reduction though better H_2_‐activation reactivity is expected.

**Table 1:** CO_2_ hydrogenation in the presence of silylhalides. 
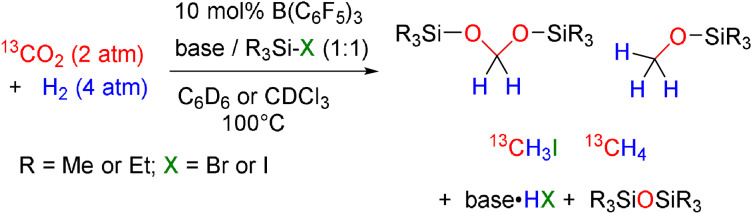

Ent	Solv.	Silylhalide^[a]^	base^[a]^	*t* [h]	Major product	Yield^[b]^
1	C_6_D_6_	Me_3_SiCl	Lut	20	‐	<1 %
2	CDCl_3_	Me_3_SiCl	Lut	20	‐	<1 %
3	C_6_D_6_	Me_3_SiBr	Lut	40	MeOSiMe_3_	83 %
4	CDCl_3_	Me_3_SiBr	Lut	60	MeOSiMe_3_	73 %
5	C_6_D_6_	Me_3_SiI	Lut	60	^13^CH_4_	76 %
6	CDCl_3_	Me_3_SiI	Lut	20	^13^CH_4_	85 %
7	C_6_D_6_	Et_3_SiI	Lut	60	(Et_3_SiO)_2_^13^CH_2_	72 %
8	CDCl_3_	Et_3_SiI	Lut	40	^13^CH_3_I	82 %
9	C_6_D_6_	Et_3_SiI	Col	40	(Et_3_SiO)_2_^13^CH_2_	8 %
10	CDCl_3_	Et_3_SiI	Col	40	MeOSiEt_3_	9 %

[a] 0.05 mmol silylhalide and Lewis base were added; Lut=2,6‐lutidine; Col=2,4,6 collidine. [b] Yields are determined by ^1^H NMR spectroscopy using 10 μL toluene as internal standard.

The above reactions demonstrate that simple tuning of the reaction conditions for FLP hydrogenation of CO_2_ provided variation of the major products. While lutidine was identified as the preferred base in the presence of the Lewis acid catalyst B(C_6_F_5_)_3_, the use of Me_3_SiBr produced Me_3_SiO^13^CH_3_, whereas Me_3_SiI afforded primarily ^13^CH_4_ as the CO_2_ reduction product. The acetal, (Et_3_SiO)_2_^13^CH_2_, was formed preferentially when Et_3_SiI was employed in C_6_D_6_ solution. Perhaps most remarkably, however was the impact of the use of Et_3_SiI in CDCl_3_ which resulted in the formation of ^13^CH_3_I as the major product (Scheme 3).^[16]^

**Scheme 3:**
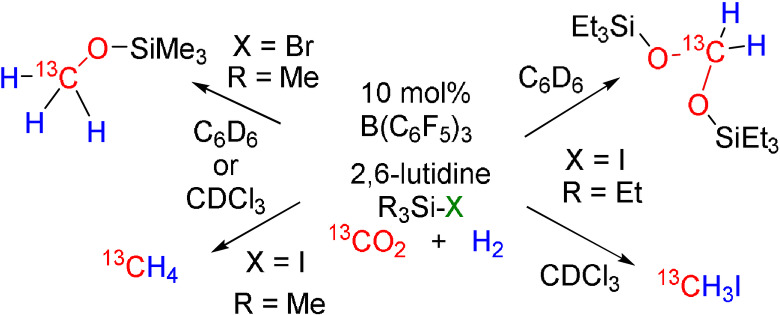
Summary of major products of CO_2_ reduction using the FLP catalyst B(C_6_F_5_)_3_/2,6‐lutidine.

Efforts to probe the reaction affording isotopically enriched methyl iodide prompted us to monitor the reaction of ^13^CO_2_ (2 atm) and D_2_ (2 atm) in the presence of 2,6‐lutidine, Et_3_SiI and 10 mol % B(C_6_F_5_)_3_ in CDCl_3_ at 100 °C. At this lower pressure and with the shorter reaction time of 24 h, the reaction was not complete. However, the NMR spectra revealed the formation of isotopologues of the acetal and methoxy species in 33 % yield and 21 % yield, respectively. The three isotopologues of the acetal, (Et_3_SiO)_2_^13^CH_2_ and (Et_3_SiO)_2_^13^CHD and (Et_3_SiO)_2_^13^CD_2_ were formed in an approximately 1:4:1 ratio. The isotopologue (Et_3_SiO)_2_^13^CHD exhibited a triplet in the ^13^C{^1^H} NMR spectrum at 84.0 ppm (^1^ *J*_C–D_=25 Hz) as well as a doublet at 5.03 ppm (^1^ *J*_C–H_=161 Hz) in the ^1^H NMR spectrum; while the (Et_3_SiO)_2_^13^CD_2_ was found as a pentet in the ^13^C{^1^H} NMR spectrum at 83.6 ppm (^1^ *J*_C–D_=25 Hz). The four isotopologues of methoxy, Et_3_SiO^13^CH_3,_ Et_3_SiO^13^CH_2_D, Et_3_SiO^13^CHD_2_ and Et_3_SiO^13^CD_3_ were generated in a 1:5:8:4 ratio, each of them was found in the ^13^C{^1^H} NMR spectrum at 50.8 ppm, 50.5 ppm, 50.2 ppm and 49.7 ppm as singlet, triplet, pentet and septet resonance with ^1^ *J*_C–D_=22 Hz, respectively. In addition, the NMR data showed the formation of H_2_ as a singlet at 4.63 ppm and HD as a triplet at 4.59 ppm (J_H–D_=43 Hz) and a triplet at 2.39 ppm (^2^ *J*_H–D_=2 Hz) adjacent the methyl resonance of 2,6‐lutidine, which is corresponding to the mono‐methyl‐deuterated 2,6‐lutidine. These data suggest that competitive to reaction with CO_2_, the product of initial activation of D_2_, [C_5_H_3_Me_2_ND][DB(C_6_F_5_)_3_], can evolve HD, generating a transient enamine, while tautomerization regenerates lutidine leading to H/D scrambling into the methyl groups of lutidine, the generation of HD and H_2_, and the generation of the isotopologues of the CO_2_ reduction products (Scheme 4). It is noteworthy that on prolonged reaction for 70 h, the above reaction gave 78 % yield of the expected isotopologues of methyl iodide, CH_3_I, CH_2_DI, CD_2_HI and CD_3_I in a 1:5:5:4 ratio. These species are observed in the ^13^C{^1^H} NMR spectrum at −23.39 ppm, −23.41 ppm, −23.44 ppm and −23.47 ppm as singlet, triplet, pentet and septet resonances, respectively. The deuterated species exhibited ^1^ *J*_C–D_ values of 23 Hz.

**Scheme 4:**
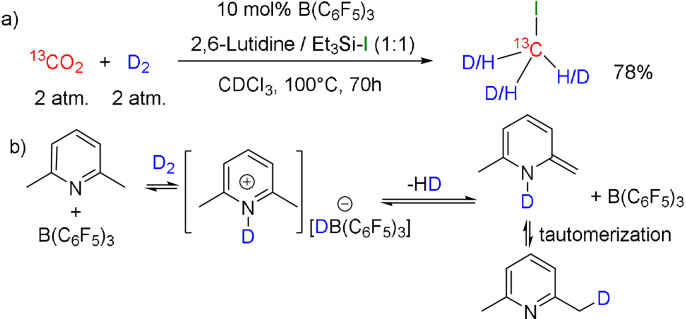
a) Deuteration of CO_2_ b) deuteration of lutidine, mediated by B(C_6_F_5_)_3_ under D_2_.

Mechanistically, the above reactivity indicates that the present hydrogenation of CO_2_ begins with the known FLP activation of H_2_ followed by the reaction with CO_2_ affording a formyl borate anion. Reaction with the silylhalide affords the silyl‐formate and frees the borane for further activation of H_2_. Hydrido‐borate attack of the silyl‐formate and reactions with the silylhalide affords the acetal and subsequently the methyloxy‐silane, although the dominance of these reactions depends on the nature of the silyl‐substituent, the halide and the solvent. In a non‐polar solvent, reaction of the methyloxy‐silane with the hydrido‐borate in the presence of the silylhalide affords methane and the disilylether. In contrast, a polar solvent favors attack by iodide, affording methyl iodide as the dominant product.

This view of the reactivity was further probed by extensive DFT calculations at the dispersion‐corrected PW6B95‐D3/def2‐QZVP + COSMO‐RS// TPSS‐D3/def2‐TZVP + COSMO level of theory in chloroform solution,^[17]^ using the typical substrates of 2,6‐lutidine (Lut), H_2_, CO_2_ and Me_3_SiI along with the Lewis‐acid B(C_6_F_5_)_3_ as the catalyst. The final PW6B95‐D3 free energies (in kcal mol^−1^, at 298 K and 1 M concentration) are discussed.

The activation of H_2_ by the separated FLP Lut/B(C_6_F_5_)_3_ (Figure 1 A) is −10.0 kcal mol^−1^ exergonic over a low free energy barrier of 15.9 kcal mol^−1^ (via **TS1**) giving the ion pair [LutH]^+^[HB(C_6_F_5_)_3_]^−^ (**A**). In CHCl_3_ solution, the separated ions are 1.1 kcal mol^−1^ less stable at room temperature but are easily accessible and even more stable upon heating due to favorable entropic effects. In contrast, both CO_2_ and Me_3_SiI cannot be activated by the FLP, as the adduct LutCOOB(C_6_F_5_)_3_ and the separated ions of [LutSiMe_3_]^+^ and I^−^, are 11.5 and 5.1 kcal mol^−1^ endergonic, respectively (see Supporting Information). However, CO_2_ is easily reduced by **A** via hydride transfer from [HB(C_6_F_5_)_3_]^−^ to the carbon with H‐bonding of [LutH]^+^ to oxygen and the formation of [LutH]^+^[HCOOB(C_6_F_5_)_3_]^−^ (**B**) is −5.3 kcal mol^−1^ exergonic over a free energy barrier of only 18.9 kcal mol^−1^ (via **TS2**). Consistent with experiment, the reduction of Me_3_SiI with **A** to form Me_3_SiH, [LutH]I and regenerated B(C_6_F_5_)_3_ catalyst is 10.1 kcal mol^−1^ endergonic and thus thermodynamically prevented (see Supporting Information). On the other hand, the reaction between Me_3_SiI and **B** is −1.6 kcal mol^−1^ exergonic and proceeds easily over a low barrier of 14.3 kcal mol^−1^ (via **TS3^−^**). This affords the neutral adduct Me_3_SiOCHOB(C_6_F_5_)_3_ (**C**) that still requires 3.9 kcal mol^−1^ to eliminate B(C_6_F_5_)_3_ and give Me_3_SiOCHO (**D**). Such trapping of B(C_6_F_5_)_3_ with **D** effectively increases the free energy barrier to the initial H_2_‐activation to 19.8 kcal mol^−1^ (via **TS1**), which is thus the rate‐limiting step for the formation of **D**. For comparison, the Lewis bases Lut, Col, Cl^−^ and Br^−^ also form stable B(C_6_F_5_) adducts that are −2.0, −4.7, −5.7 and −1.3 kcal mol^−1^ exergonic in CHCl_3_ solution (see Supporting Information), respectively. The higher affinity for Col and Cl^−^ may further inhibit H_2_‐activation reactivity.

**Figure 1:**
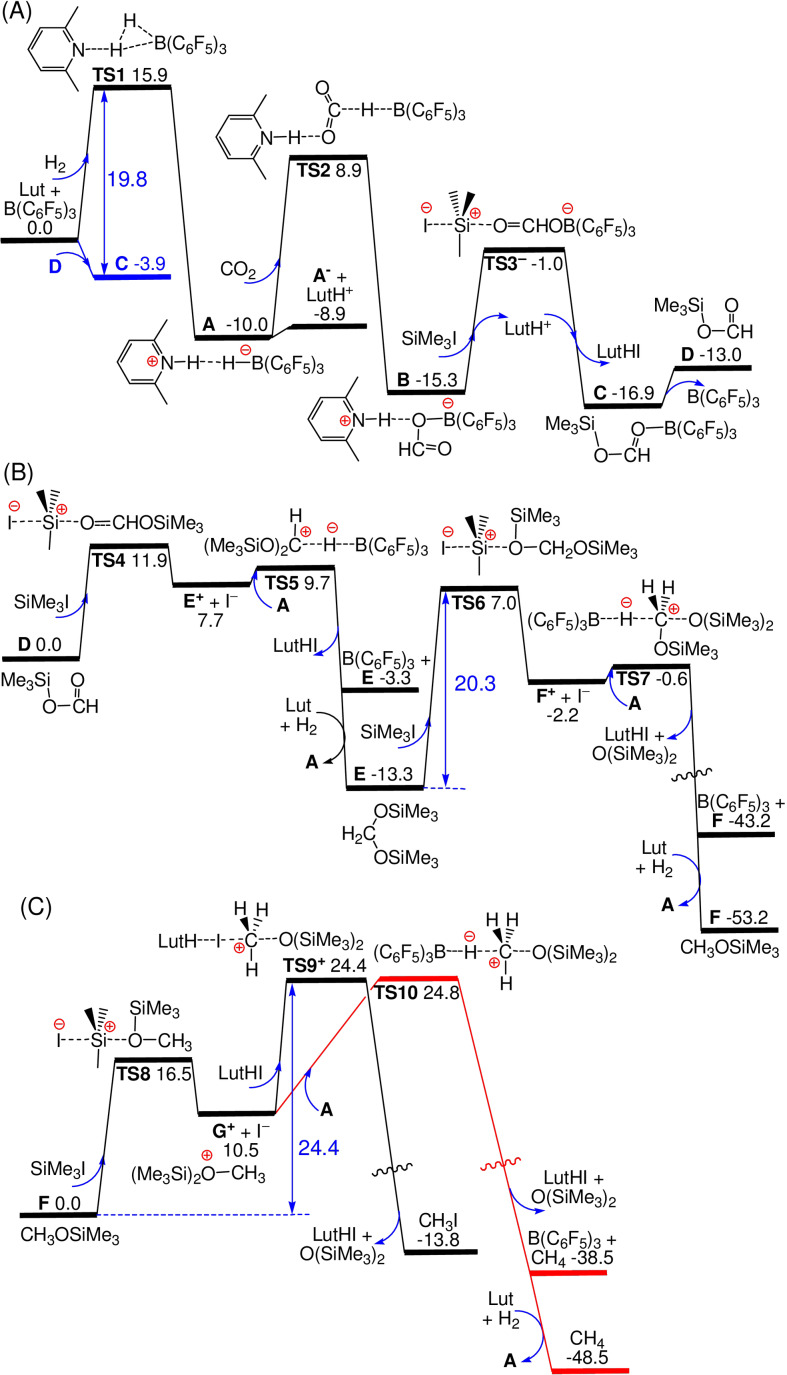
DFT‐computed free energy paths for: A) the lutidine/B(C_6_F_5_)_3_ FLP‐mediated H_2_ activation and further reduction of CO_2_ into HCOOSiMe_3_; B) further reduction into H_2_C(OSiMe_3_)_2_ and even H_3_COSiMe_3_; C) slower and kinetically competitive formation of CH_3_I and CH_4_.

Once intermediate **D** is formed (Figure 1 B), further reduction via silylium transfer from Me_3_SiI (via **TS4**) and subsequent hydride transfer from **A** (via **TS5**) to give the acetal H_2_C(OSiMe_3_)_2_ (**E**) proceeds quickly and is −13.3 kcal mol^−1^ exergonic. Further silylium transfer from Me_3_SiI to **E** (via **TS6**) and subsequent hydride transfer from **A** (via **TS7**) to give H_3_COSiMe_3_ (**F**), O(SiMe_3_)_2_ and [LutH]I is still possible over a slightly higher barrier of 20.3 kcal mol^−1^ (via **TS6)**, but is −39.9 kcal mol^−1^ exergonic. Under moderate heating, both formation of **E** and **F** should be kinetically facile. The use of bulkier silanes such as Et_3_SiI may enhance the barrier to silylium transfer and thus slow formation of **F**, making selective acetal formation possible in less polar benzene solution (Table 1, entry 7).

Silylium transfer from Me_3_SiI to **F** to give the cation H_3_CO(SiMe_3_)_2_^+^ (**G^+^**) and the I^−^ anion (via **TS8**, Figure 1 C), is 10.5 kcal mol^−1^ endergonic over a low barrier of 16.5 kcal mol^−1^ and thus is kinetically feasible. Further nucleophilic iodide transfers from [LutH]I to **G^+^** to give the experimentally observed CH_3_I and O(SiMe_3_)_2_ is −24.3 kcal mol^−1^ exergonic over a low barrier of 13.9 kcal mol^−1^ (via **TS9^+^**). The overall formation of CH_3_I from **F** is thus −13.8 kcal mol^−1^ exergonic over a sizable barrier of 24.4 kcal mol^−1^, consistent with the moderate heating required experimentally. On the other hand, nucleophilic hydride transfer from **A** to **G^+^** to give CH_4_, O(SiMe_3_)_2_ and regenerate B(C_6_F_5_)_3_ is −49.0 kcal mol^−1^ exergonic over a low barrier of 14.3 kcal mol^−1^ (via **TS10**). Coupled with the facile H_2_ activation, the overall formation of CH_4_ from **F** is thus −53.2 kcal mol^−1^ exergonic over a barrier of 24.8 kcal mol^−1^. This is thermodynamically more favorable but kinetically comparable with the formation of CH_3_I. Indeed, the use of Et_3_SiI and Me_3_SiI are found to favor iodide and hydride transfer affording CH_3_I and CH_4_, respectively.

In conclusion, we have achieved metal‐free catalytic hydrogenation of CO_2_ using H_2_ and a silylhalide as an oxophile in the presence of a FLP derived from lutidine and B(C_6_F_5_)_3_. The judicious selection of the steric demands and nature of the silylhalide and the solvent provides control of these catalytic reductions affording avenues to the selective formation of the methoxysilane, Me_3_SiO^13^CH_3,_ the acetal (Et_3_SiO)_2_^13^CH_2_, ^13^CH_4_ and ^13^CH_3_I. The complexities of the mechanisms involved have been detailed using DFT studies. We are continuing to explore the use of FLPs in reactions of interest.

**Supporting Information available**: Synthetic and spectral data, computational details and DFT‐computed energies and Cartesian coordinates are deposited.

## Conflict of interest

The authors declare no conflict of interest.

## Supporting information

As a service to our authors and readers, this journal provides supporting information supplied by the authors. Such materials are peer reviewed and may be re‐organized for online delivery, but are not copy‐edited or typeset. Technical support issues arising from supporting information (other than missing files) should be addressed to the authors.

Supporting Information
