# Supplementary material for: Selective Catalytic Frustrated Lewis Pair Hydrogenation of CO2 in the Presence of Silylhalides
Source: Angew Chem Int Ed Engl. 2021 Nov 3;60(49):25771–5. doi: 10.1002/anie.202112233 (PMC9298004; doi:10.1002/anie.202112233)
Supplement: Supplementary file 1 — Supporting Information [file ANIE-60-25771-s001.pdf]

Angewandte Chemie
Eine Zeitschrift der Gesellschaft Deutscher Chemiker

Supporting Information

Selective Catalytic Frustrated Lewis Pair Hydrogenation of CO_2 in the Presence of Silylhalides

Tongtong Wang[+], Maotong Xu[+], Andrew R. Jupp, Zheng-Wang Qu, Stefan Grimme, and Douglas W. Stephan**

anie_202112233_sm_miscellaneous_information.pdf

Contents

1. General Considerations 2

2. Stoichiometric reaction between [Lut-H][$HCO_2B(C_6F_5)_3$] and Et_3Si-I 2

3. Stoichiometric reaction between [Lut-H][I] and $B(C_6F_5)_3$ 4

4. Attempts to reduce Et_3Si-I to Et_3Si-H by $B(C_6F_5)_3$/2,6-Lutidine FLP and H_2 6

5. $B(C_6F_5)_3$/2,6-Lutidine FLP and halosilanes with H_2/CO_2 6

General Procedure 7

Entry 1: $B(C_6F_5)_3$/2,6-Lutidine/Me_3Si-Cl in C_6D_6 7

Entry 2: $B(C_6F_5)_3$/2,6-Lutidine/Me_3Si-Cl in $CDCl_3$ 8

Entry 3: $B(C_6F_5)_3$/2,6-Lutidine/Me_3Si-Br in C_6D_6 9

Entry 4: $B(C_6F_5)_3$/2,6-Lutidine/Me_3Si-Br in $CDCl_3$ 11

Entry 5: $B(C_6F_5)_3$/2,6-Lutidine/Me_3Si-I in C_6D_6 13

Entry 6: $B(C_6F_5)_3$/2,6-Lutidine/Me_3Si-I in $CDCl_3$ 16

Entry 7: $B(C_6F_5)_3$/2,6-Lutidine/Et_3Si-I in C_6D_6 20

Entry 8: $B(C_6F_5)_3$/2,6-Lutidine/Et_3Si-I in $CDCl_3$ 21

6. $B(C_6F_5)_3$/2,4,6-Collidine FLP and halosilanes with H_2/CO_2 23

Entry 9: $B(C_6F_5)_3$/2,4,6-Collidine/Et_3Si-I in C_6D_6 24

Entry 10: $B(C_6F_5)_3$/2,4,6-Collidine/Et_3Si-I in $CDCl_3$ 25

7. $B(C_6F_5)_3$/2,6-Lutidine FLP and halosilanes with D_2/CO_2 27

Reaction for 24 hours 27

Reaction for 70 hours 30

8. Experimental References 32

9. DFT computational Details: 34

Computational References 63

1. General Considerations

Unless otherwise specified, all manipulations were performed under an inert atmosphere of dry, oxygen-free N_2 using Schlenk techniques or glovebox. 4 Å molecular sieves was purchased from Sigma Aldrich, activated by heating at 300°C under vacuum for 2 days. Solvents were dried by a Grubbs type Innovative Technologies solvent purification system, degassed on Schlenk line and stored over activated 4 Å molecular sieves prior to use. $CDCl_3$ was dried over calcium hydride, distilled and stored in activated 4 Å molecular sieves prior to use. C_6D_6 was degassed on Schlenk line and stored in activated 4 Å molecular sieves prior to use. All glassware was dried in a 180 °C oven overnight prior to use. $B(C_6F_5)_3$ was first purified by sublimination at 110°C under vacuum, the sublimed $B(C_6F_5)_3$ was dissolved in minimum amount of pentane at room temperature. Storing at a -25°C freezer afforded pure $B(C_6F_5)_3$ as white crystalline solids. Carbon ^{13}C dioxide (99 atom % ^{13}C, <3 atom % ^{18}O) were purchased from Sigma Aldrich. [Lut-H][$HCO_2B(C_6F_5)_3$] was synthesized according to literature method.[1] NMR spectra were obtained at room temperature on Bruker Advance III 400 MHz, Bruker Ultrashield 400 MHz, Agilent DD2 500 MHz and Agilent DD2 600 MHz spectrometer. NMR chemical shifts are reported in ppm. Multiplicity is reported as follows: s = singlet, d = doublet, t = triplet, m = multiplet, b = broad.

2. Stoichiometric reaction between [Lut-H][$HCO_2B(C_6F_5)_3$] and Et_3Si-I

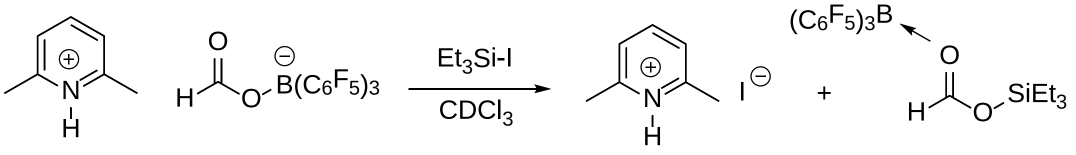

[Lut-H][$HCO_2B(C_6F_5)_3$][1] (113 mg, 0.17 mmol) and Et_3Si-I (30 µL, 0.17 mmol) were mixed in 0.5 mL $CDCl_3$. The reaction was completed within 10 minutes at room temperature, afforded 2,6-lutidinium iodide [Lut-H][I] and $Et_3SiOCHO{\cdot}B(C_6F_5)_3$ adduct, which has NMR data agreed with the literature values.[2]

[Lut-H][$HCO_2B(C_6F_5)_3$]: ^1H NMR (400 MHz, $CDCl_3$) δ 8.31 (s, 1H), 8.18 (t, *J* = 7.9 Hz, 1H), 7.49 (d, *J* = 7.9 Hz, 2H), 2.72 (s, 6H); $^{11}B\{^1H\}$ NMR (128 MHz, $CDCl_3$) δ -2.62; $^{19}F\{^1H\}$ NMR (377 MHz, $CDCl_3$) δ -134.59 (dd, *J* = 23.9, 8.4 Hz), -158.96 (t, *J* = 20.3 Hz), -164.90 (ddd, *J* = 23.6, 19.2, 8.5 Hz).

[Lut-H][I] and $Et_3SiOCHO{\cdot}B(C_6F_5)_3$: ^1H NMR (400 MHz, $CDCl_3$) 8.22 (t, *J* = 8.0 Hz, 1H), 8.17 (s, 1H), 7.53 (d, *J* = 7.9 Hz, 2H), 2.98 (s, 6H), 1.12 – 0.73 (m, 15H, Et); $^{11}B\{^1H\}$ NMR (128 MHz, $CDCl_3$) δ -0.11; ^{19}F NMR (377 MHz, $CDCl_3$) δ -133.89 (d, *J* = 22.4 Hz), -158.13 (t, *J* = 20.3 Hz), -164.78 (m).

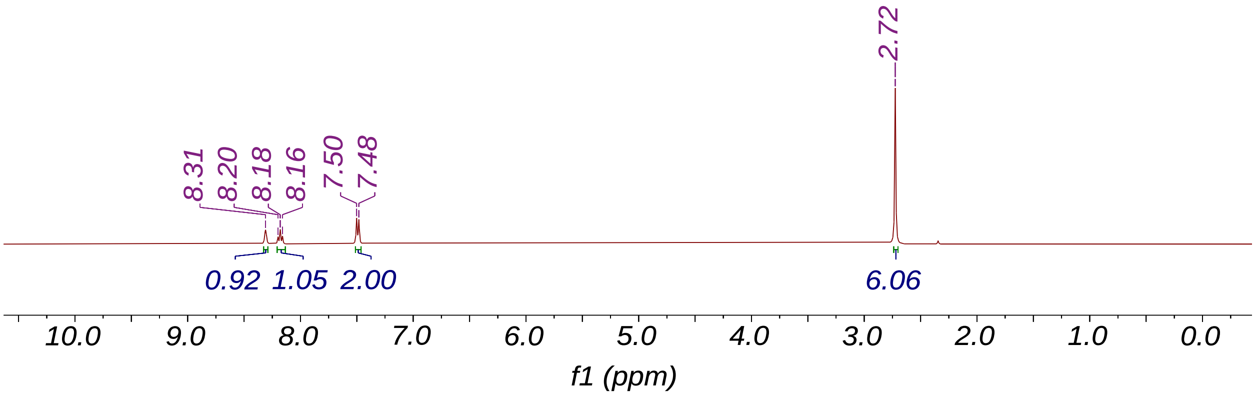

Figure S 1. ^{1}H NMR spectrum of [Lut-H][$HCO_2B(C_6F_5)_3$], $CDCl_3$

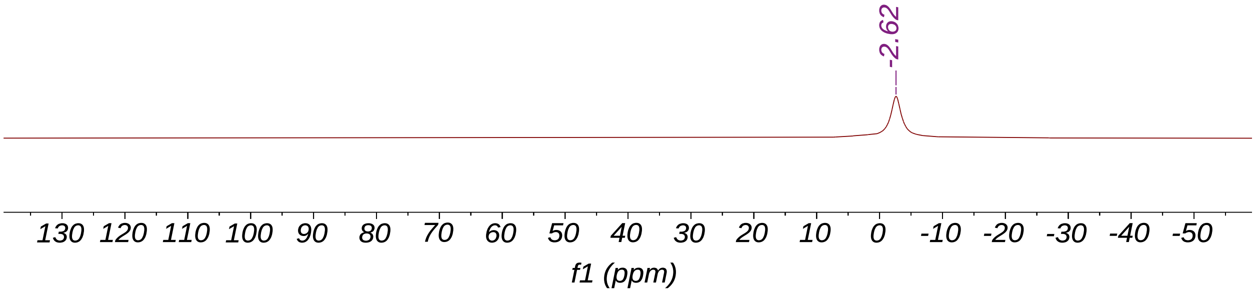

Figure S 2. ^{11}B{^{1}H} NMR spectrum of [Lut-H][$HCO_2B(C_6F_5)_3$], $CDCl_3$

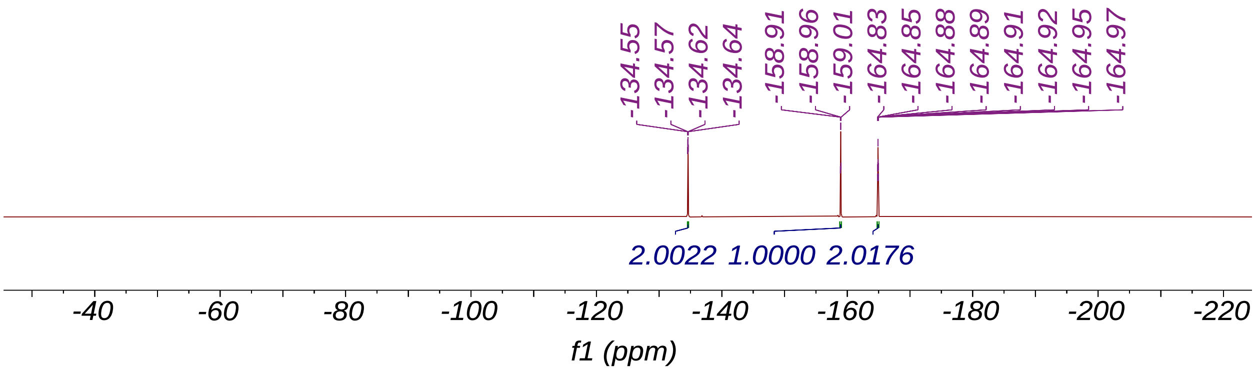

Figure S 3. ^{19}F{^{1}H} NMR spectrum of [Lut-H][$HCO_2B(C_6F_5)_3$], $CDCl_3$

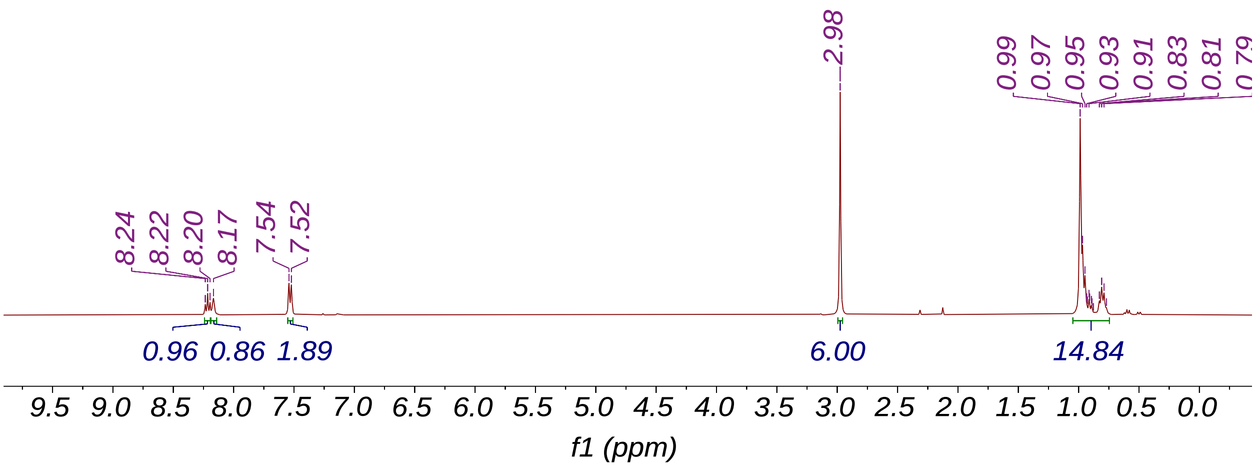

Figure S 4. ^{1}H NMR spectrum of [Lut-H][I] and $Et_3SiOCHO{\cdot}B(C_6F_5)_3$, $CDCl_3$

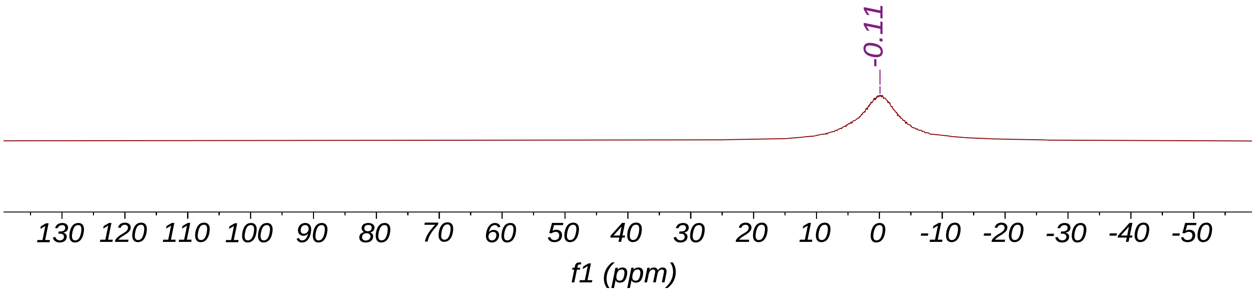

Figure S 5. $^{11}B\{^{1}H\}$ NMR spectrum of [Lut-H][I] and $Et_3SiOCHO{\cdot}B(C_6F_5)_3$, $CDCl_3$

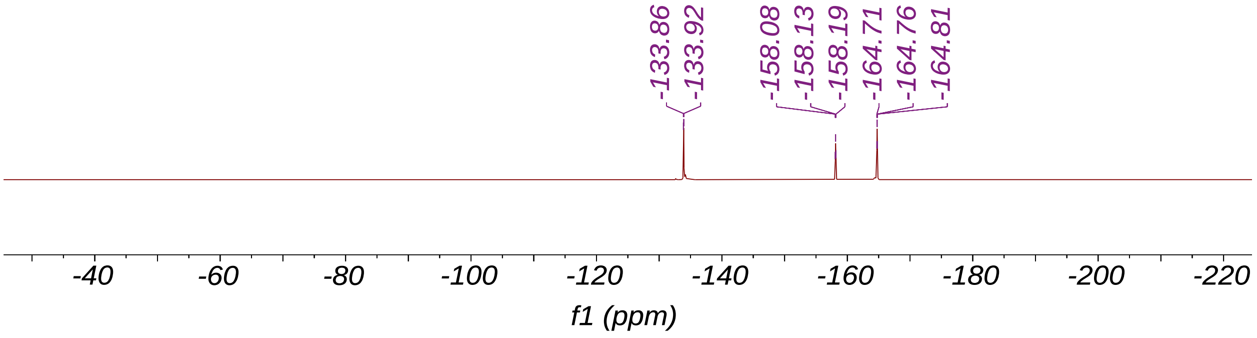

Figure S 6. $^{19}F\{^{1}H\}$ NMR spectrum of [Lut-H][I] and $Et_3SiOCHO{\cdot}B(C_6F_5)_3$, $CDCl_3$

3. Stoichiometric reaction between [Lut-H][I] and $B(C_6F_5)_3$

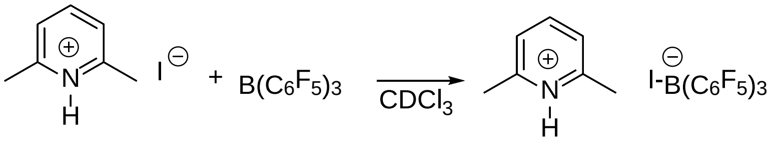

[Lut-H][I] (2.3 mg, 0.01 mmol) and $B(C_6F_5)_3$ (5.0 mg, 0.01 mmol) were mixed in 0.5 mL $CDCl_3$. The reaction was completed within 10 minutes at room temperature, afforded the corresponding adduct [Lut-H][I·$B(C_6F_5)_3$].

^{1}H NMR (400 MHz, $CDCl_3$) δ 15.16 (t, J = 56.5 Hz, 1H), 8.20 (t, J = 7.9 Hz, 1H), 7.51 (d, J = 8.0 Hz, 2H), 3.04 (s, 6H); ^{11}B{^{1}H} NMR (128 MHz, $CDCl_3$) δ 43.33; ^{19}F{^{1}H} NMR (377 MHz, $CDCl_3$) δ -127.87 (bs), -146.13 (bs), -161.11 (bs).

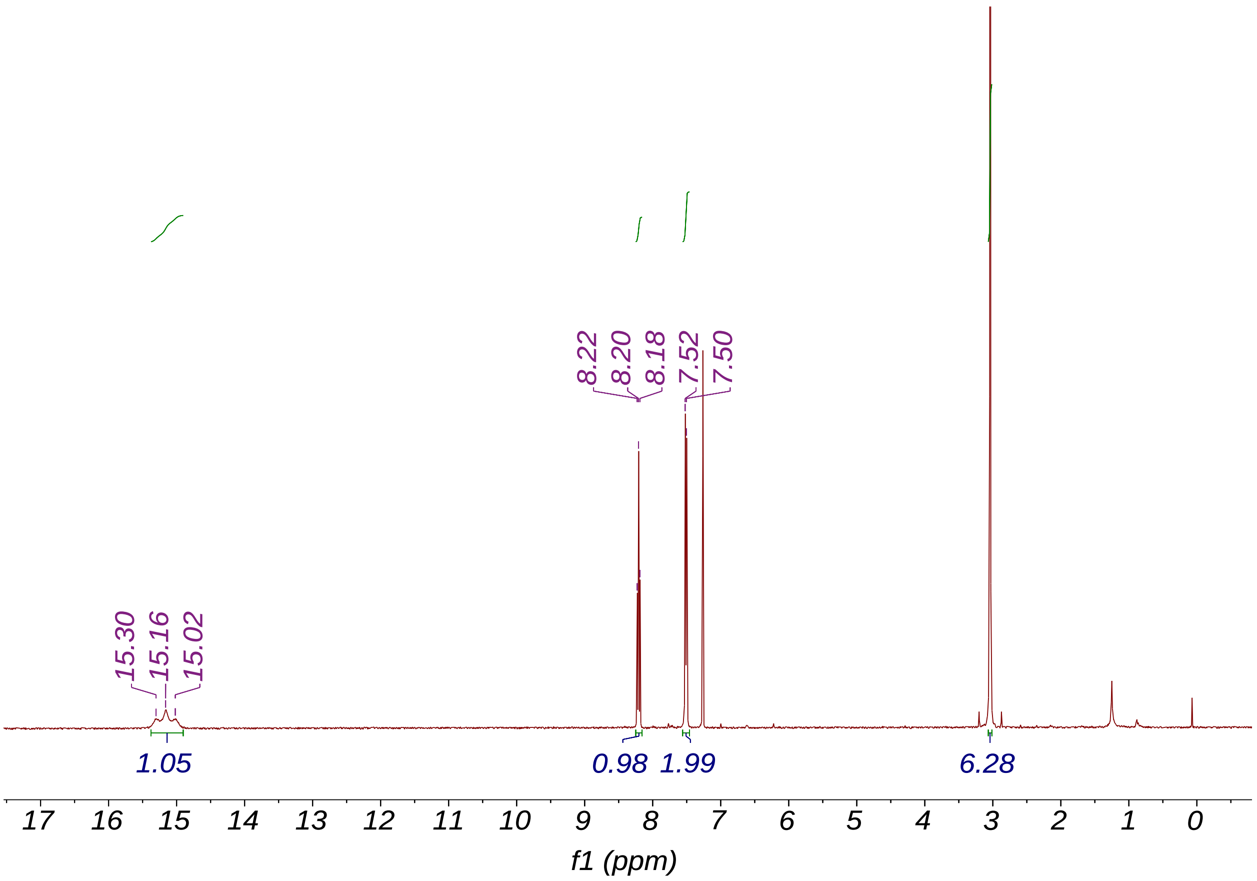

Figure S 7. ^{1}H NMR spectrum of [Lut-H][I·B(C_6F_5)$_3$], $CDCl_3$

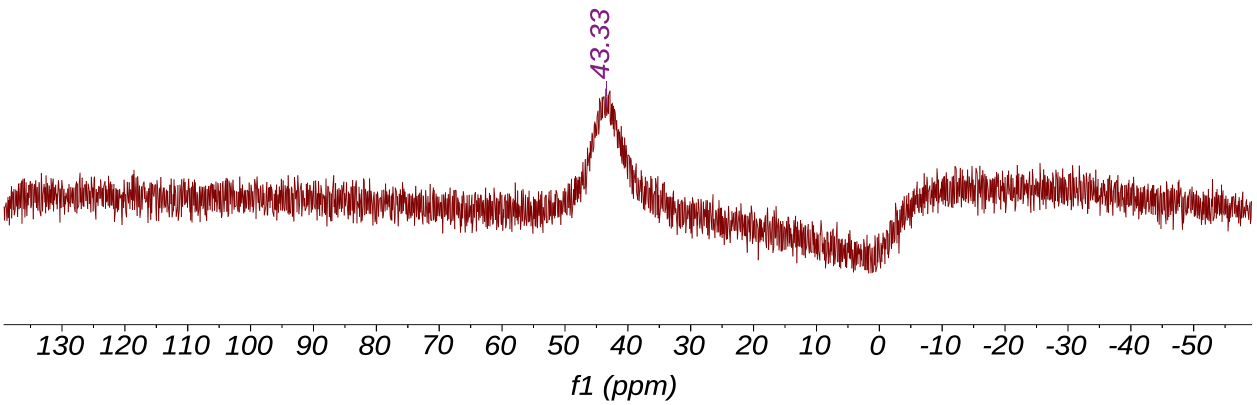

Figure S 8. $^{11}B\{^1H\}$ NMR spectrum of [Lut-H][I·B(C_6F_5)$_3$], $CDCl_3$

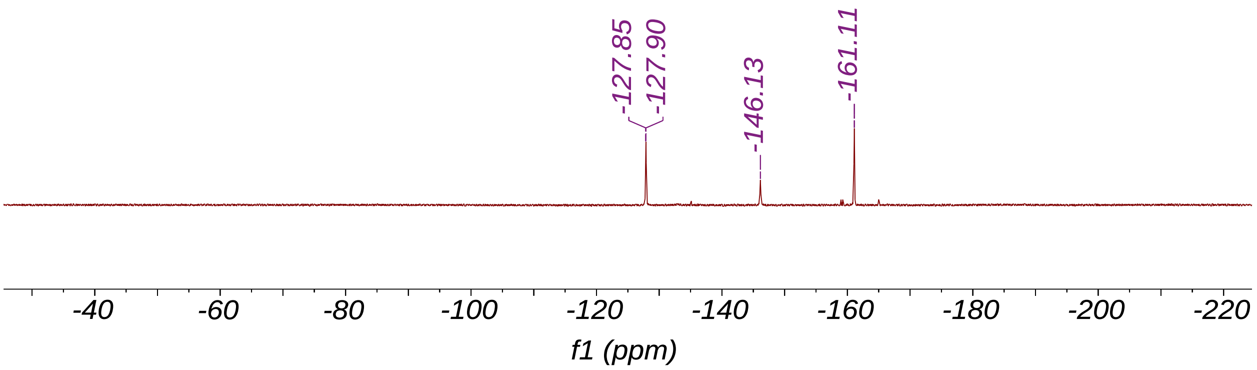

Figure S 9. $^{19}F\{^1H\}$ NMR spectrum of [Lut-H][I·B(C_6F_5)$_3$], $CDCl_3$

4. Attempts to reduce Et_3Si-I to Et_3Si-H by B(C_6F_5)$_3$/2,6-Lutidine FLP and H_2

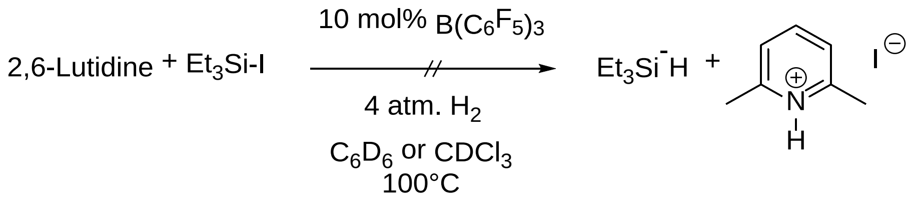

10 mol% B(C_6F_5)$_3$ (2.6 mg, 0.0051 mmol) in 0.4 mL C_6D_6 or 0.4 mL $CDCl_3$ was transferred to a J-young tube, followed by the addition of Et_3Si-I (8.9 µL, 0.051 mmol, 10 eq), 2,6-lutidine (6.0 µL, 0.051 mmol, 10 eq). After freeze-pump-thaw degassing, 4 atm. H_2 was added to the J-young tubes. No reaction was observed after overnight at room temperature. The J-young tubes were heated in a 100°C oil bath for 40 hours, no formation of Et_3Si-H was seen indicated by the absence of Si-H signal at 3.85 ppm in C_6D_6 or 3.61 ppm in $CDCl_3$.[3]

5. B(C_6F_5)$_3$/2,6-Lutidine FLP and halosilanes with H_2/CO_2

General Procedure

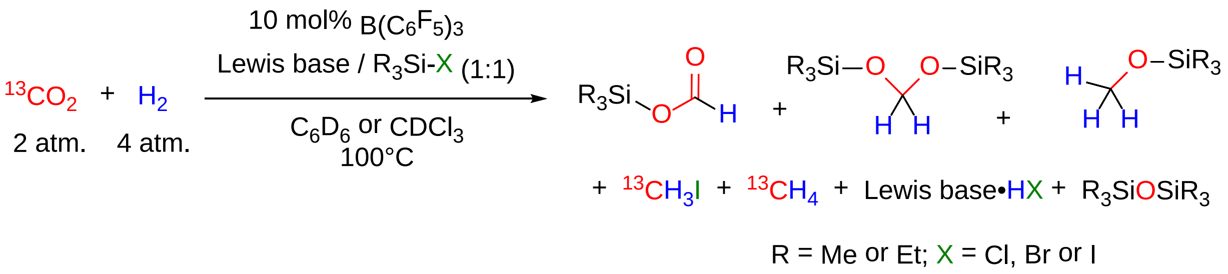

10 mol% $B(C_6F_5)_3$ (2.6 mg, 0.0051 mmol) in 0.4 mL C_6D_6 was transferred to a J-young tube, followed by the addition of Me_3Si-I (7.3 µL, 0.051 mmol, 10 eq), 2,6-lutidine (6.0 µL, 0.051 mmol, 10 eq) and 10 µL toluene (internal standard). After freeze-pump-thaw degassing, the solution was kept frozen in a liquid nitrogen bath, 2 atm. $^{13}CO_2$ and 4 atm. H_2 was added to the J-young tube. After warming up back to room temperature, the J-young tube was heated in a 100°C oil bath.

Note: hydrogenation of 2,6-lutidine or 2,4,6-collidine to the corresponding amines was also observed under elongated heating.[4]

Entry 1: $B(C_6F_5)_3$/2,6-Lutidine/Me_3Si-Cl in C_6D_6

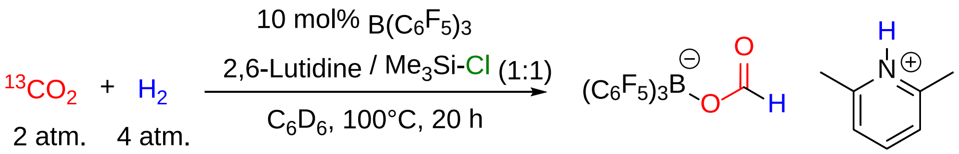

^{1}H NMR (400 MHz, C_6D_6) δ 8.37 (d, J = 209.0 Hz, $[C_5H_3Me_2NH][\underline{H}^{13}CO_2B(C_6F_5)_3]$).

$^{13}C\{^1H\}$ NMR (101 MHz, C_6D_6) δ 169.5 ($[C_5H_3Me_2NH][H\underline{^{13}C}O_2B(C_6F_5)_3]$); ^{13}C NMR (101 MHz, C_6D_6) δ 169.5 (d, J = 208.3 Hz, $[C_5H_3Me_2NH][H\underline{^{13}C}O_2B(C_6F_5)_3]$).

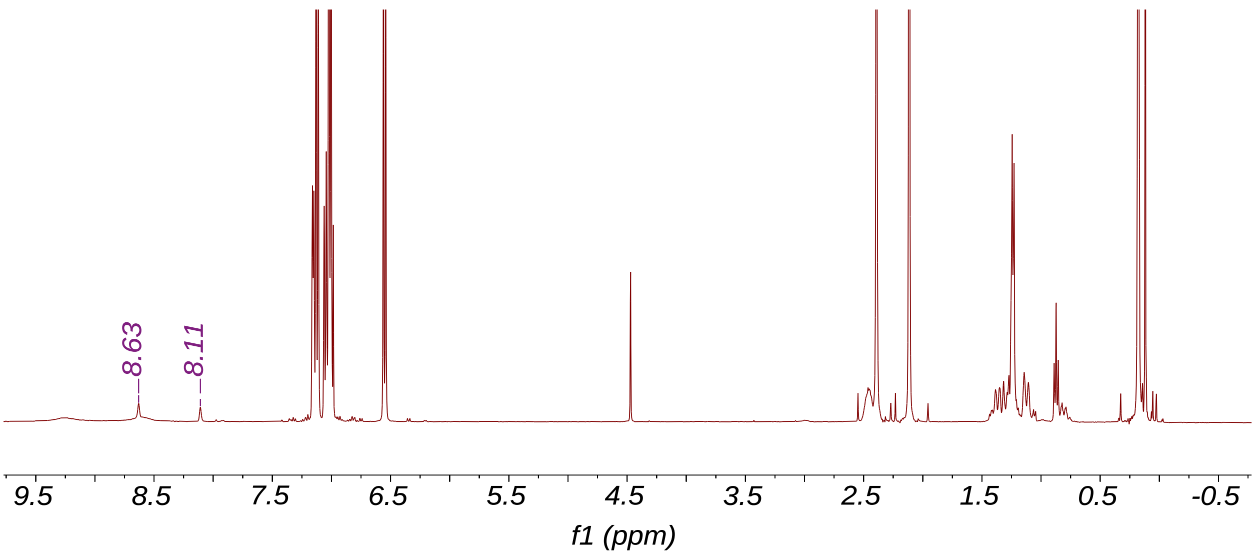

Figure S 10. *^{1}H NMR of $B(C_6F_5)_3$/2,6-Lutidine/Me_3Si-Cl, 20 hours at 100°C, C_6D_6*

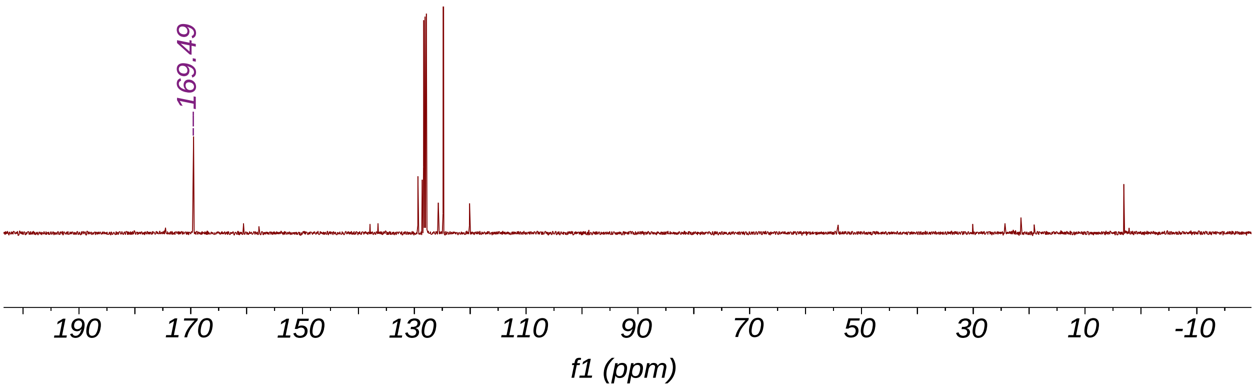

Figure S 11. *$^{13}C\{^{1}H\}$ NMR of $B(C_6F_5)_3$/2,6-Lutidine/Me_3Si-Cl, 20 hours at 100°C, C_6D_6*

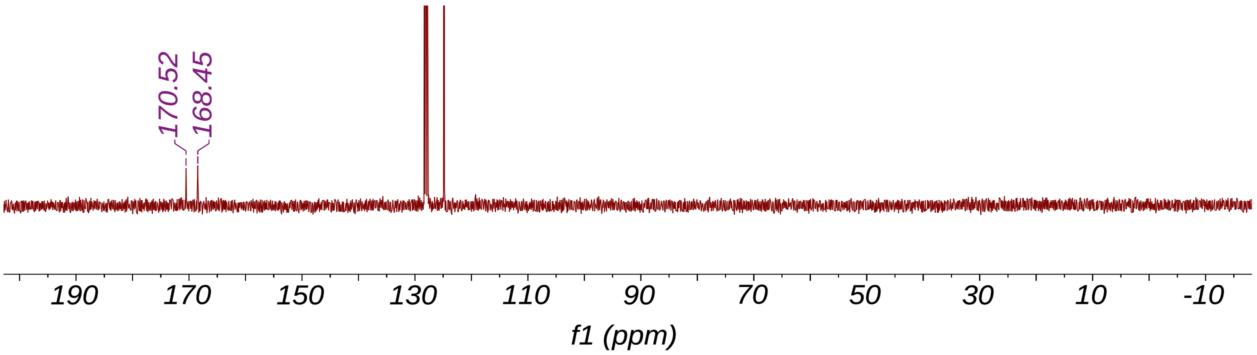

Figure S 12. *^{13}C NMR of $B(C_6F_5)_3$/2,6-Lutidine/Me_3Si-Cl, 20 hours at 100°C, C_6D_6*

Entry 2: $B(C_6F_5)_3$/2,6-Lutidine/Me_3Si-Cl in $CDCl_3$

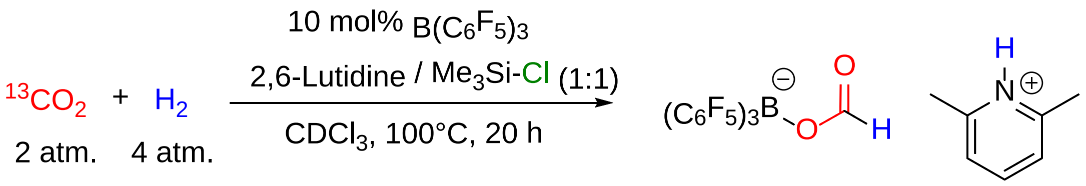

^{1}H NMR (400 MHz, $CDCl_3$) δ 8.24 (d, *J* = 209.1 Hz, $[C_5H_3Me_2NH][\underline{H}^{13}CO_2B(C_6F_5)_3]$);

$^{13}C\{^{1}H\}$ NMR (101 MHz, $CDCl_3$) δ 169.4 ($[C_5H_3Me_2NH][H^{13}\underline{C}O_2B(C_6F_5)_3]$).

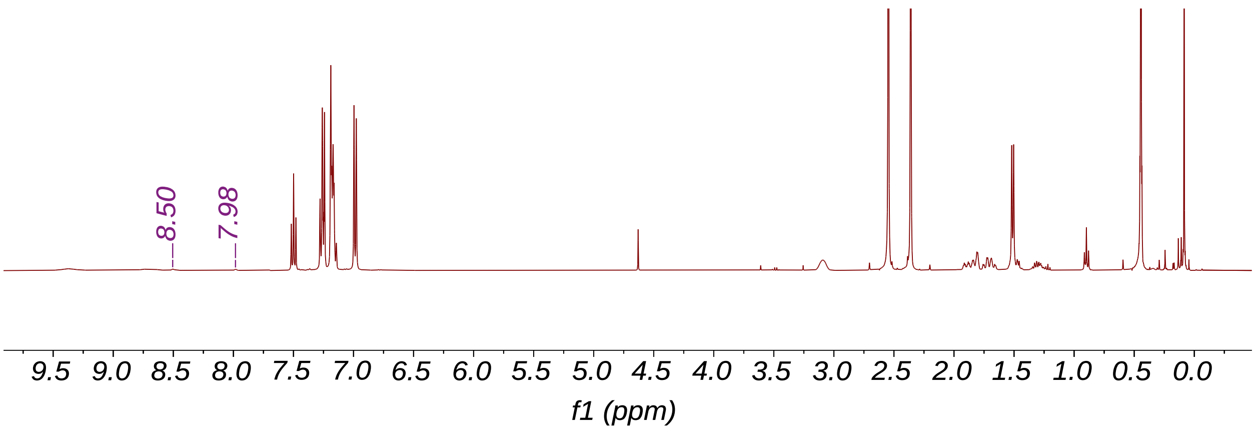

Figure S 13. ^{1}H NMR of $B(C_6F_5)_3$/2,6-Lutidine/Me_3Si-Cl, 20 hours at 100°C, $CDCl_3$

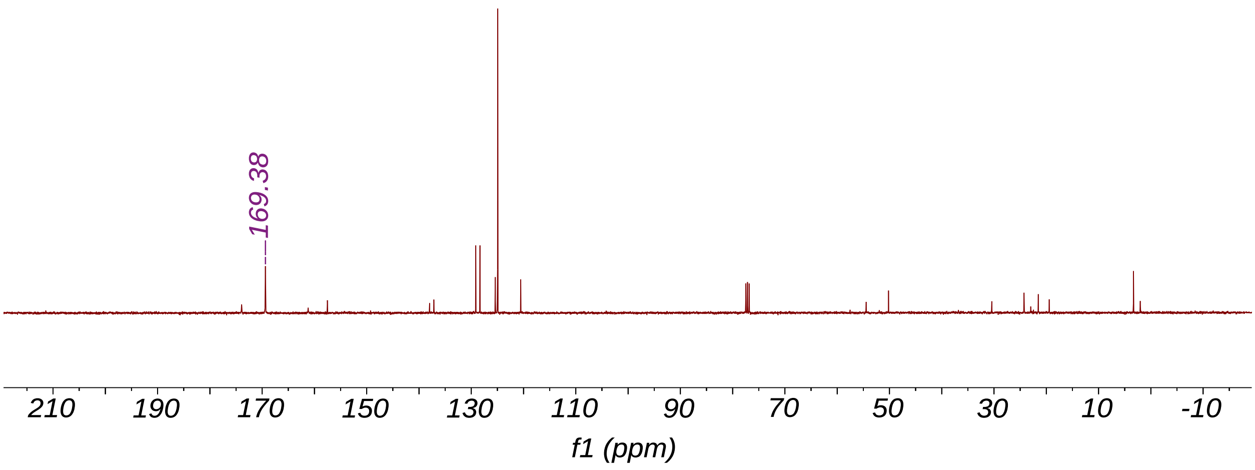

Figure S 14. $^{13}C\{^{1}H\}$ NMR of $B(C_6F_5)_3$/2,6-Lutidine/Me_3Si-Cl, 20 hours at 100°C, $CDCl_3$

Entry 3: $B(C_6F_5)_3$/2,6-Lutidine/Me_3Si-Br in C_6D_6

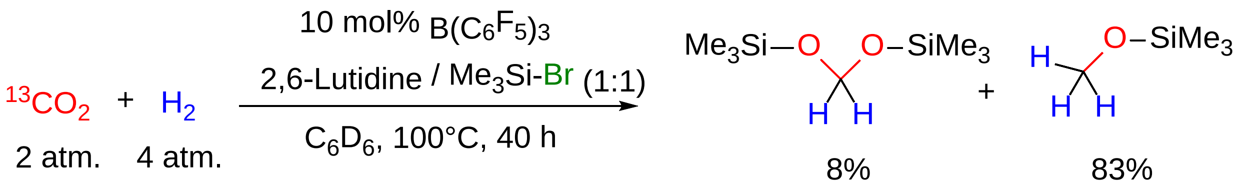

^{1}H NMR (400 MHz, C_6D_6) δ 5.02 (d, *J* = 162.3 Hz, $(Me_3SiO)_2{}^{13}CH_2$), 3.25 (d, *J* = 141.0 Hz, $Me_3SiO^{13}CH_3$).

$^{13}C\{^{1}H\}$ NMR (101 MHz, C_6D_6) δ 84.4 ($(Me_3SiO)_2{}^{13}CH_2$), 49.9 ($Me_3SiO^{13}CH_3$).

Overall yield = 91% at 40 hours, $(Me_3SiO)_2{}^{13}CH_2$: $Me_3SiO^{13}CH_3$= 1:11

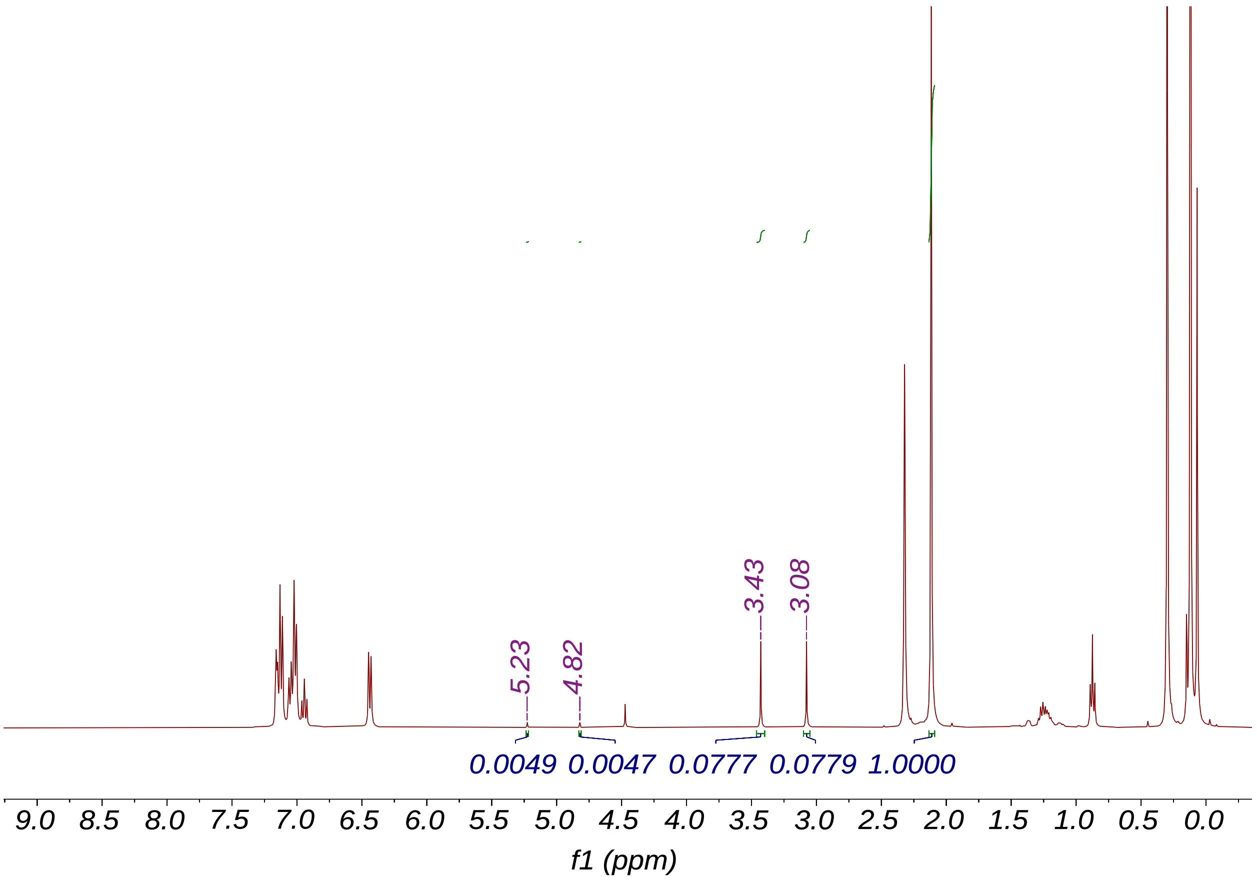

Figure S 15. ^{1}H NMR of $B(C_6F_5)_3$/2,6-Lutidine/Me_3Si-Br, 40 hours at 100°C, C_6D_6

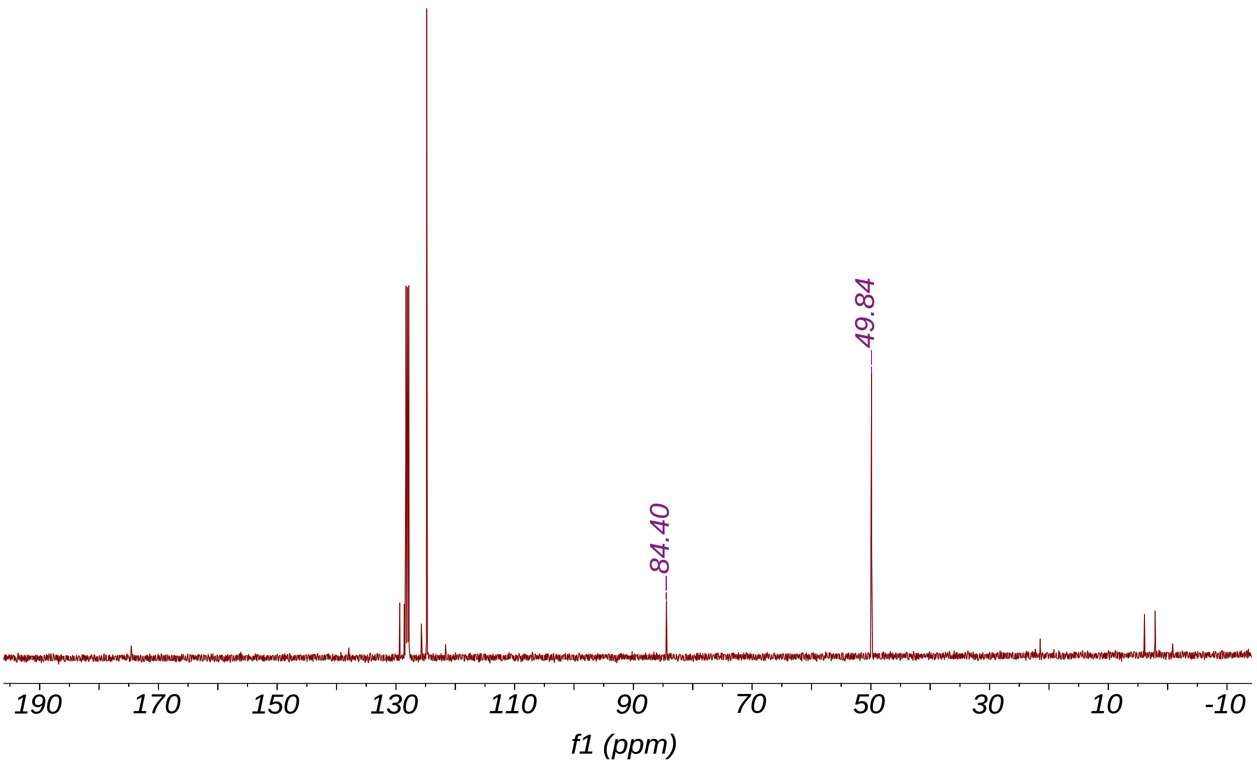

Figure S 16. $^{13}C\{^{1}H\}$ *NMR of* $B(C_6F_5)_3$*/2,6-Lutidine/*Me_3Si*-Br, 40 hours at 100°C,* C_6D_6

Entry 4: $B(C_6F_5)_3$/2,6-Lutidine/Me_3Si-Br in $CDCl_3$

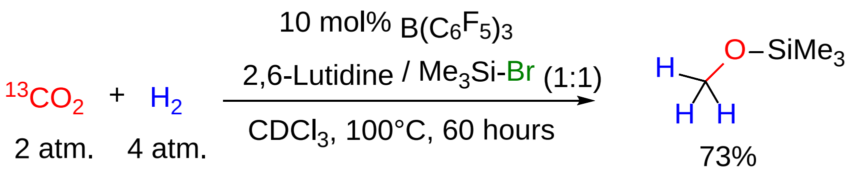

^{1}H NMR (400 MHz, $CDCl_3$) δ 3.43 (d, *J* = 141.5 Hz, $Me_3SiO^{13}C\underline{H}_3$).

$^{13}C\{^{1}H\}$ NMR (101 MHz, $CDCl_3$) δ 50.2 ($Me_3SiO\underline{^{13}C}H_3$); ^{13}C NMR (101 MHz, $CDCl_3$) δ 50.2 (q, *J* = 141.5 Hz, $Me_3SiO\underline{^{13}C}H_3$).

Overall yield = 73% at 60 hours, $Me_3SiO^{13}CH_3$ only

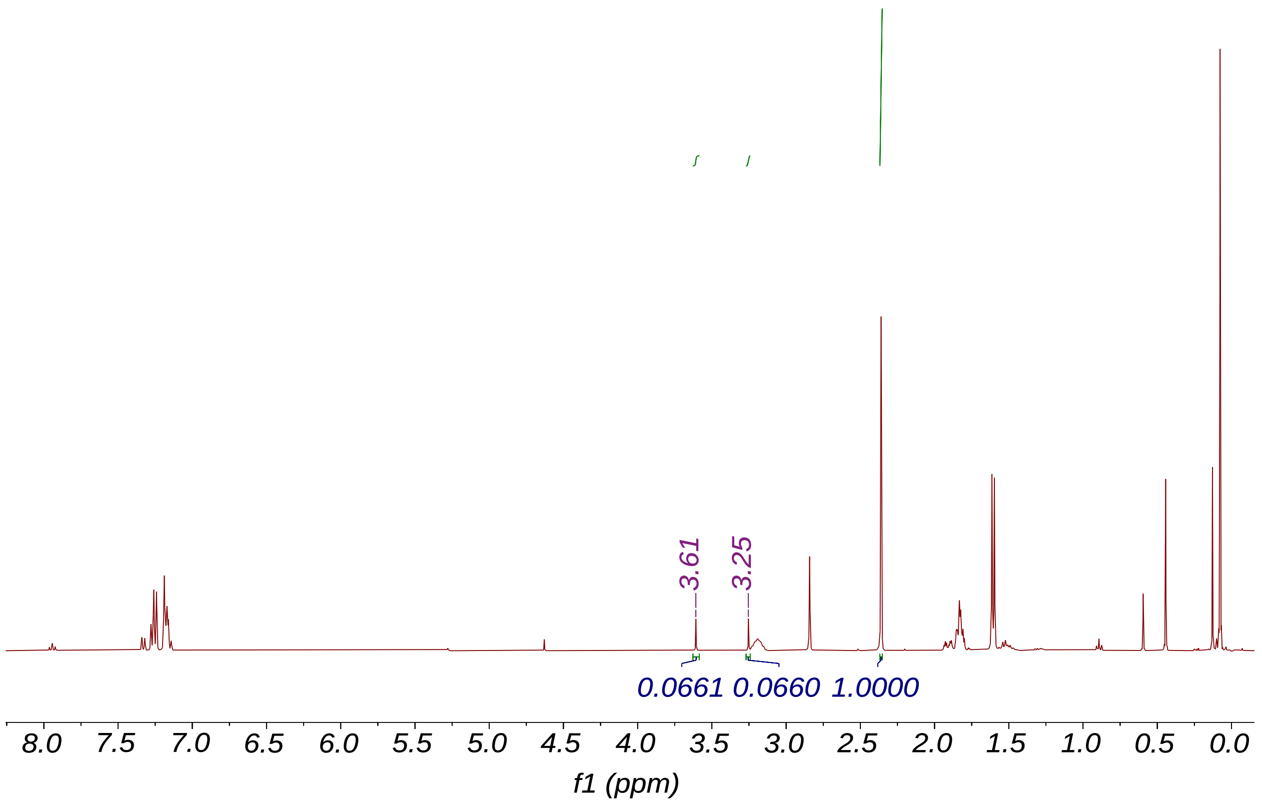

Figure S 17. ^{1}H NMR of $B(C_6F_5)_3$/2,6-Lutidine/Me_3Si-Br, 60 hours at 100°C, $CDCl_3$

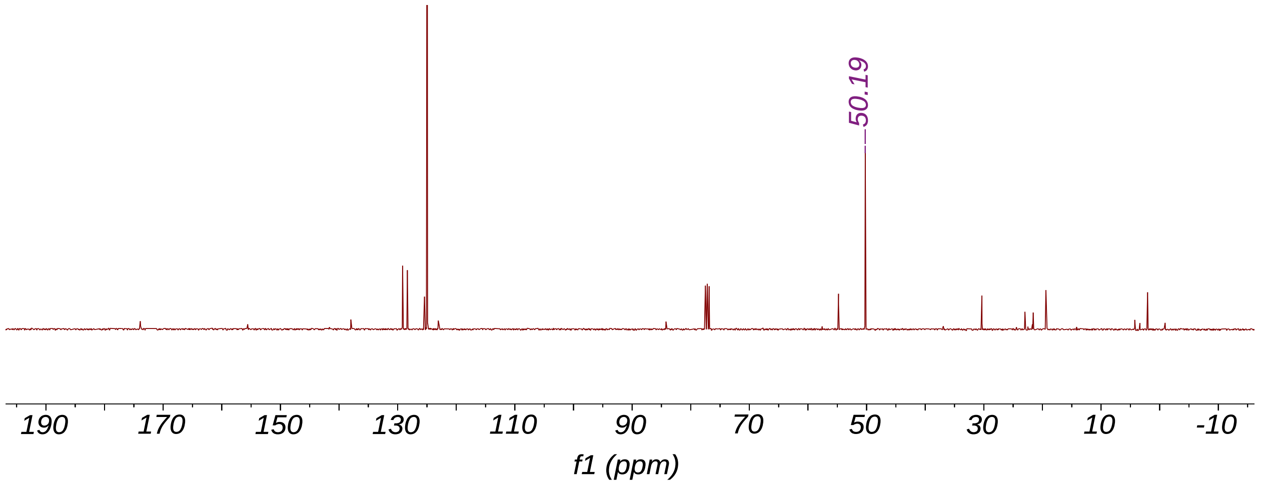

Figure S 18. $^{13}C\{^{1}H\}$ NMR of $B(C_6F_5)_3$/2,6-Lutidine/Me_3Si-Br, 60 hours at 100°C, $CDCl_3$

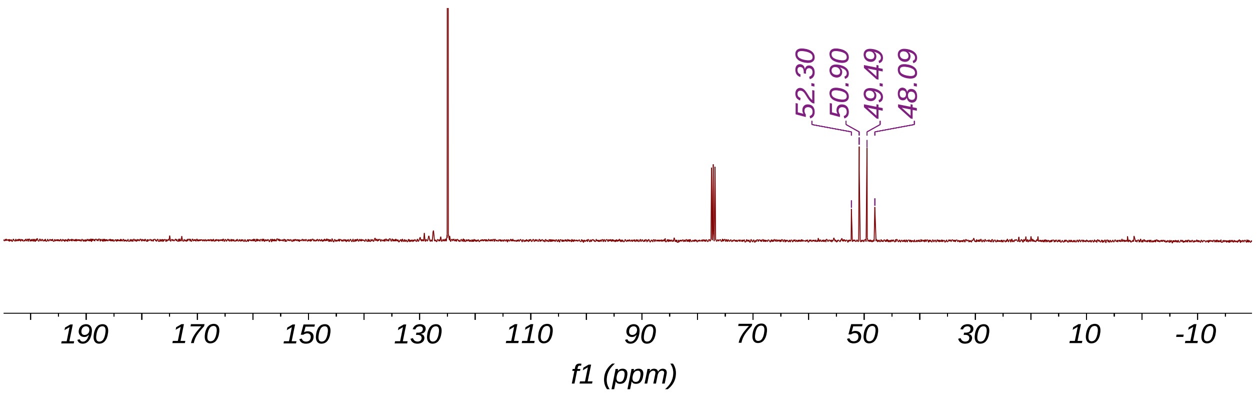

Figure S 19. ^{13}C NMR of $B(C_6F_5)_3$/2,6-Lutidine/Me_3Si-Br, 60 hours at 100°C, $CDCl_3$

Entry 5: $B(C_6F_5)_3$/2,6-Lutidine/Me_3Si-I in C_6D_6

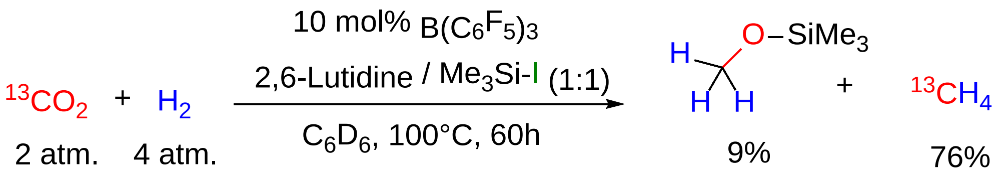

^1H NMR (400 MHz, C_6D_6) δ 3.25 (d, J = 141.0 Hz, $Me_3SiO^{13}CH_3$);

$^{13}C\{^1H\}$ NMR (101 MHz, C_6D_6) δ 49.8 ($Me_3SiO^{13}CH_3$), -4.3 ($^{13}CH_4$);

^{13}C NMR (101 MHz, C_6D_6) δ 49.8 (q, J = 141.0 Hz, $Me_3SiO^{13}CH_3$), -4.3 (p, J = 125.6 Hz, $^{13}CH_4$).

Overall yield = 85% at 60 hours. $^{13}CH_4$: $Me_3SiO^{13}CH_3$= 8.4:1

❖ *The overall yield at 60 hours = yield of methoxy species + yield of $^{13}CH_4$.*

(The yield of $^{13}CH_4$ was determined based on the method reported by Chen et al[2], the solubility of methane is estimated to be 0.021 M/atm according to the reported value and Henry's law[5])

1. Number of methoxy species is determined from ^{1}H NMR spectrum, using 0.01 mL toluene as internal standard
2. Number of protons in the methyl groups of 0.01 mL toluene: 0.01 mL * 0.867 (g/mL) / 92.14 (g/mol) * 3 * 1000 (mmol/mol) = 0.2823 mmol
3. Number of methoxy species = number of protons in the methyl groups of 0.01 mL toluene * (⅓ * integration of acetal species / integration of methyl group of toluene) = ⅓ * 0.2823 mmol * 0.017 = 0.0016 mmol
4. Yield of methoxy species = number of methoxy species / theoretical number of methoxy species = 0.0016 mmol / 0.017 mmol * 100% = 9 %
5. The total number of methane = number of methane in solution ($n_{solution}$) + number of methane in gas phase(n_{gas})
6. Number of methane in solution ($n_{solution}$) is derived from the integration of methoxy species in ^{13}C NMR spectrum
7. Number of methane in solution ($n_{solution}$) = integration of methane / integration of methoxy species * number of methoxy species = (0.5261 / 1) * 0.0016 mmol = 0.00084 mmol
8. Concentration of methane in solution = number of methane in solution / volume of solution = 0.00084 mmol / 0.4 mL = 0.0021 mmol/mL = 0.0021 M
9. Pressure of methane in gas phase (P) = concentration of methane in solution / K_H = 0.0021 M / 0.021 M/atm = 0.1 atm
10. Number of methane in gas phase(n_{gas}) is determined using ideal gas law: n_{gas} = PV/RT = 0.1 atm * 2.2 mL / 82.057 mL atm K^{-1} mol^{-1} / 298.15 K * 1000 mmol/mol = 0.0090 mmol
11. The total number of methane = number of methane in solution ($n_{solution}$) + number of methane in gas phase(n_{gas}) = 0.00084 mmol + 0.0090 mmol = 0.0098 mmol
12. Yield of methane = total number of methane / theoretical number of methane = 0.0098 mmol / 0.01285 mmol * 100% = 76%
13. The overall yield = yield of methane + yield of methoxy species = 76% + 9% = 85%
14. The ratio of methane: methoxy species = 76% : 9% = 8.4 : 1

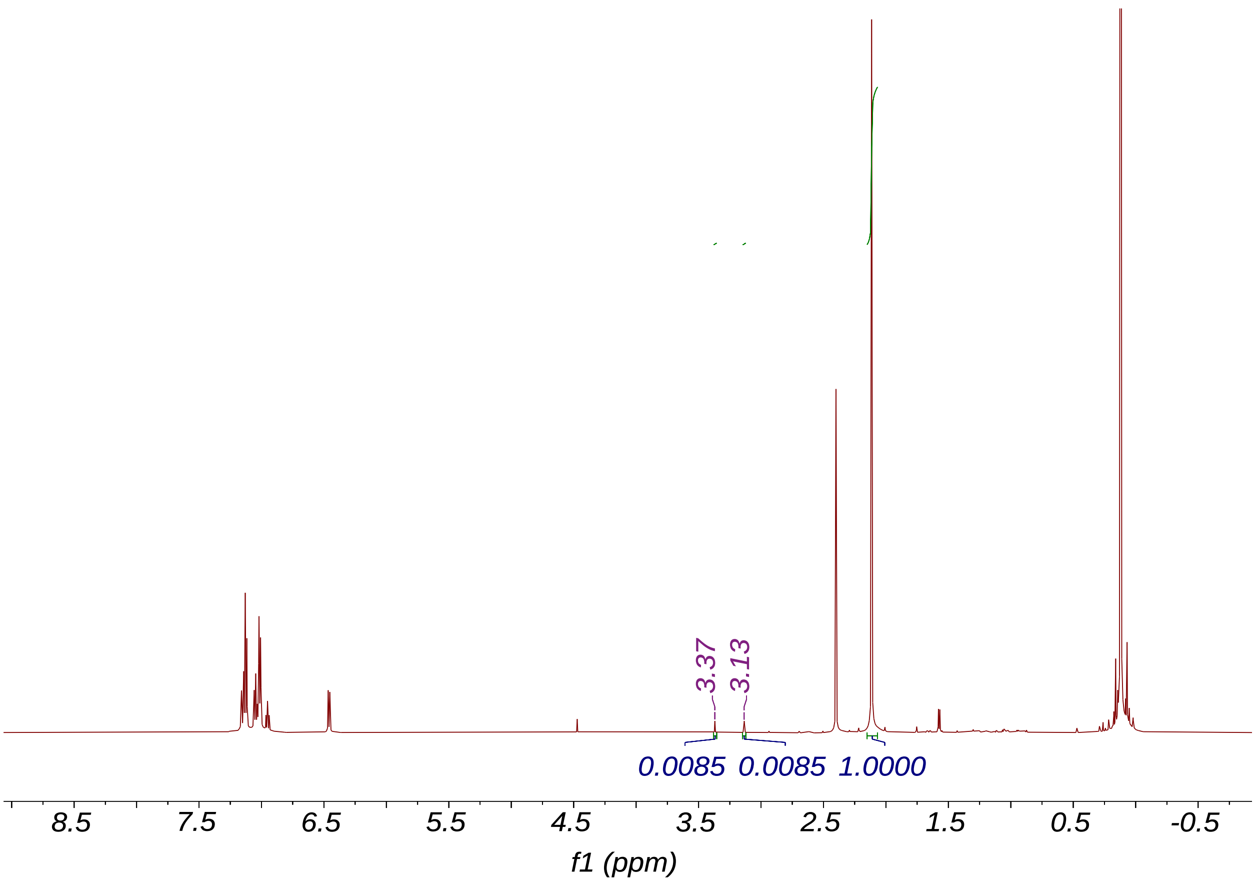

Figure S 20. ^{1}H NMR of $B(C_6F_5)_3$/2,6-Lutidine/Me_3Si-I, 60 hours at 100°C, C_6D_6

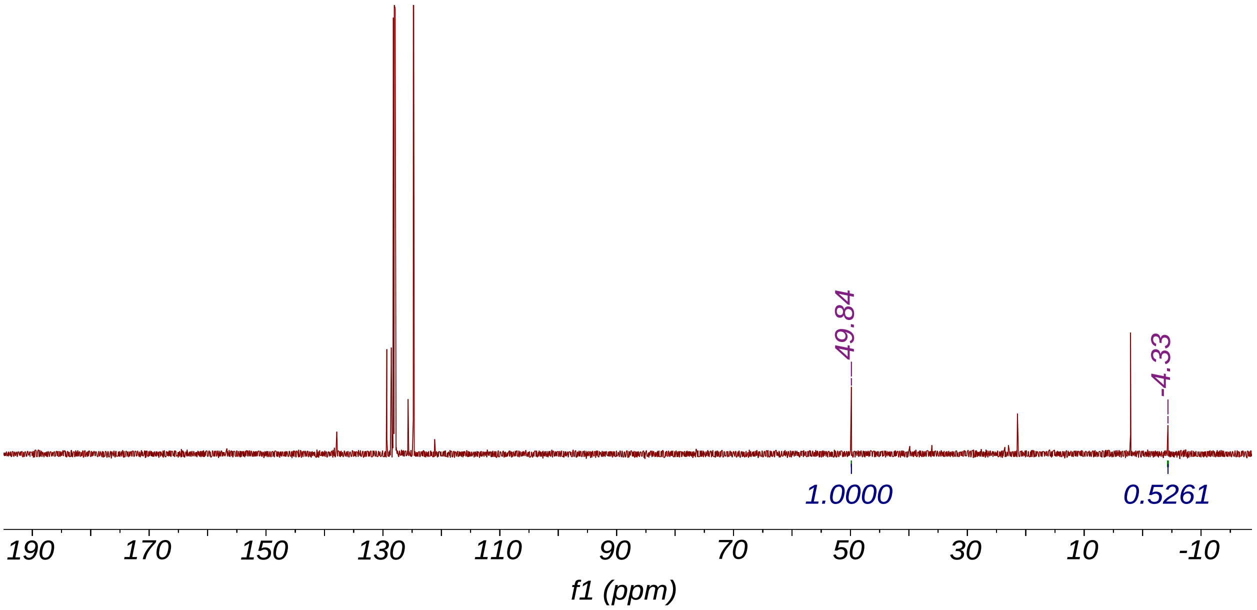

Figure S 21. $^{13}C\{^{1}H\}$ NMR of $B(C_6F_5)_3$/2,6-Lutidine/Me_3Si-I, 60 hours at 100°C, C_6D_6

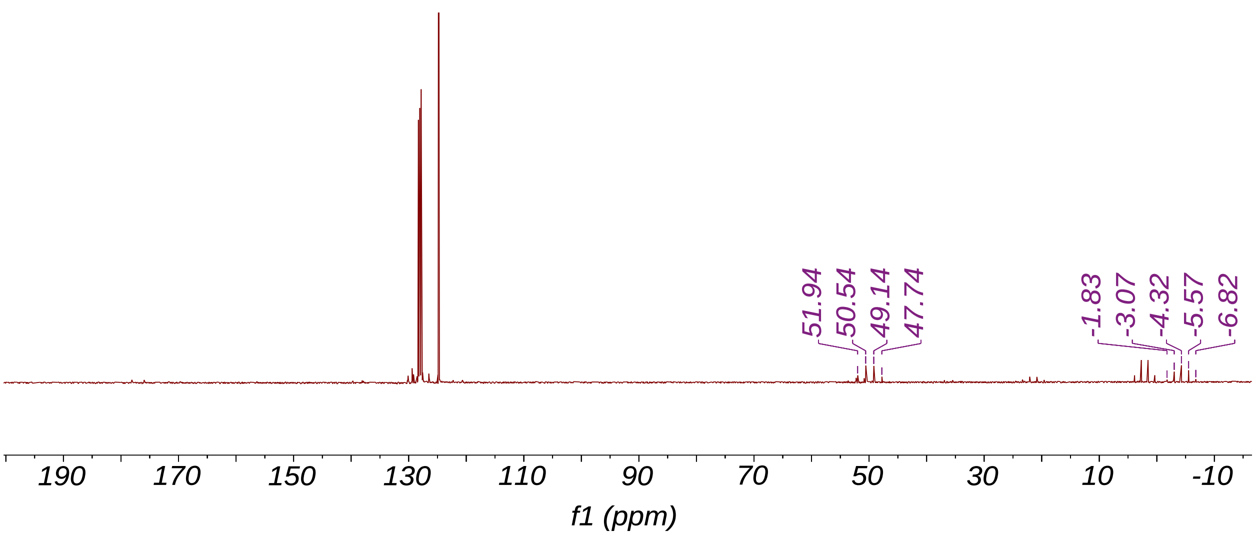

Figure S 22. ^{13}C NMR of $B(C_6F_5)_3$/2,6-Lutidine/Me_3Si-I, 60 hours at 100°C, C_6D_6

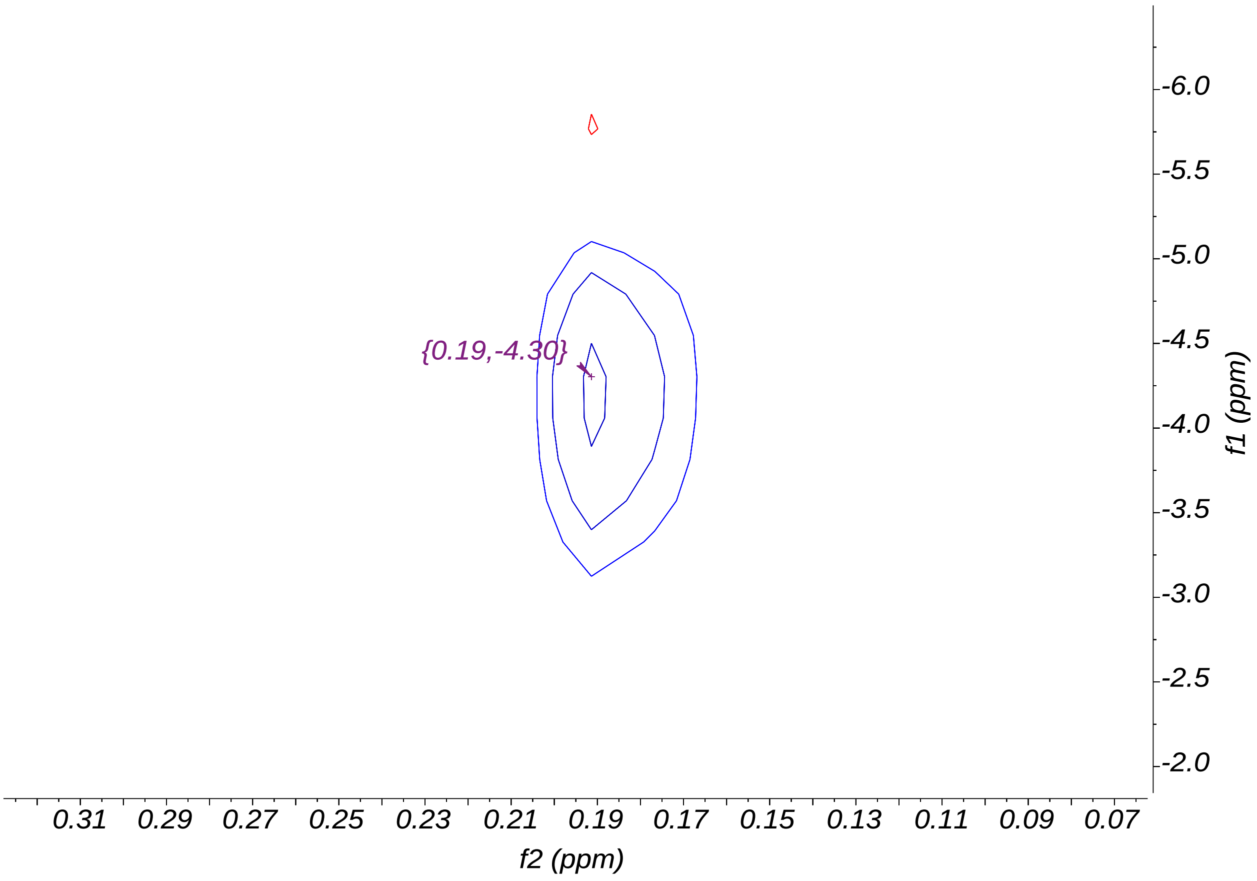

Figure S 23. HSQC spectrum of $B(C_6F_5)_3$/2,6-Lutidine/Me_3Si-I, 60 hours at 100°C, C_6D_6

Entry 6: $B(C_6F_5)_3$/2,6-Lutidine/Me_3Si-I in $CDCl_3$

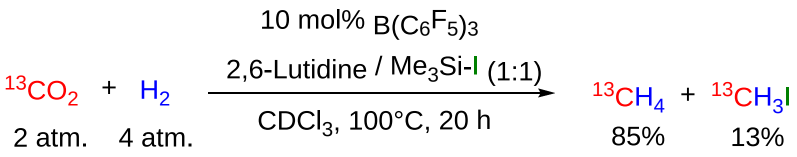

^{1}H NMR (400 MHz, $CDCl_3$) δ 2.15 (d, *J* = 151.2 Hz, $^{13}C\underline{H}_3I$), 0.23 (d, *J* = 125.6 Hz, $^{13}C\underline{H}_4$).

$^{13}C\{^{1}H\}$ NMR (101 MHz, $CDCl_3$) δ -4.2 ($\underline{^{13}C}H_4$), -23.5 ($\underline{^{13}C}H_3I$); ^{13}C NMR (101 MHz, $CDCl_3$) δ -4.2 (p, *J* = 125.6 Hz, $\underline{^{13}C}H_4$, observed as a triplet due to low intensity), -23.5 (q, *J* = 151.3 Hz, $\underline{^{13}C}H_3I$).

Overall yield = 98% at 20 hours, $^{13}CH_4$: $^{13}CH_3I$ = 6.5:1

❖ *The yield of $^{13}CH_3I$ at 20 hours:*

1. The total number of $^{13}CH_3I$ is calculated from the integration in ^1H NMR spectrum using 0.01 mL toluene as internal standard.
2. Number of protons in the methyl groups of 0.01 mL toluene: 0.01 mL * 0.867 (g/mL) / 92.14 (g/mol) * 3 * 1000 (mmol/mol) = 0.2823 mmol
3. Number of $^{13}CH_3I$ = number of protons in the methyl groups of 0.01 mL toluene * (⅓* integration of $^{13}C\text{-}H_3I$ / integration of methyl group of toluene) = ⅓* 0.2823 mmol * 0.0184 = 0.0017 mmol
4. Yield of $^{13}CH_3I$ = number of $^{13}CH_3I$ / theoretical number of $^{13}CH_3I$ = 0.0017 mmol / 0.01285 mmol * 100% = 13%

❖ *The yield of $^{13}CH_4$ at 20 hours:*

1. The total number of methane = number of methane in solution ($n_{solution}$) + number of methane in gas phase(n_{gas}).
2. Number of methane in solution ($n_{solution}$) is derived from the integration in ^1H NMR spectrum using 0.01 mL toluene as internal standard.
3. Number of protons in the methyl groups of 0.01 mL toluene: 0.01 mL * 0.867 (g/mL) / 92.14 (g/mol) * 3 * 1000 (mmol/mol) = 0.2823 mmol
4. Number of methane in solution = number of protons in the methyl groups of 0.01 mL toluene * (¼* integration of $^{13}C\text{-}H_4$ / integration of methyl group of toluene) = ¼* 0.2823 mmol * 0.0132 = 0.00093 mmol
5. Concentration of methane in solution = number of methane in solution / volume of solution = 0. 00093 mmol / 0.4 mL = 0.0023 mmol/mL = 0. 0023 M
6. Pressure of methane in gas phase (P) = concentration of methane in solution / K_H = 0. 0023 M / 0.021 M/atm = 0.11 atm
7. Number of methane in gas phase(n_{gas}) is determined using ideal gas law: n_{gas} = PV/RT = 0.11 atm * 2.2 mL / 82.057 mL atm K^{-1} mol^{-1} / 298.15 K * 1000 mmol/mol = 0.010 mmol
8. The total number of methane = number of methane in solution ($n_{solution}$) + number of methane in gas phase(n_{gas}) = 0.00093 mmol + 0.010 mmol = 0.0109 mmol
9. Yield of methane = total number of methane / theoretical number of methane = 0.0109 mmol / 0.01285 mmol * 100% = 85%
10. The overall yield = yield of $^{13}CH_4$+ yield of $^{13}CH_3I$ = 85% + 13% = 98%
11. The ratio of $^{13}CH_4$: $^{13}CH_3I$ = 85%: 13% = 6.5:1

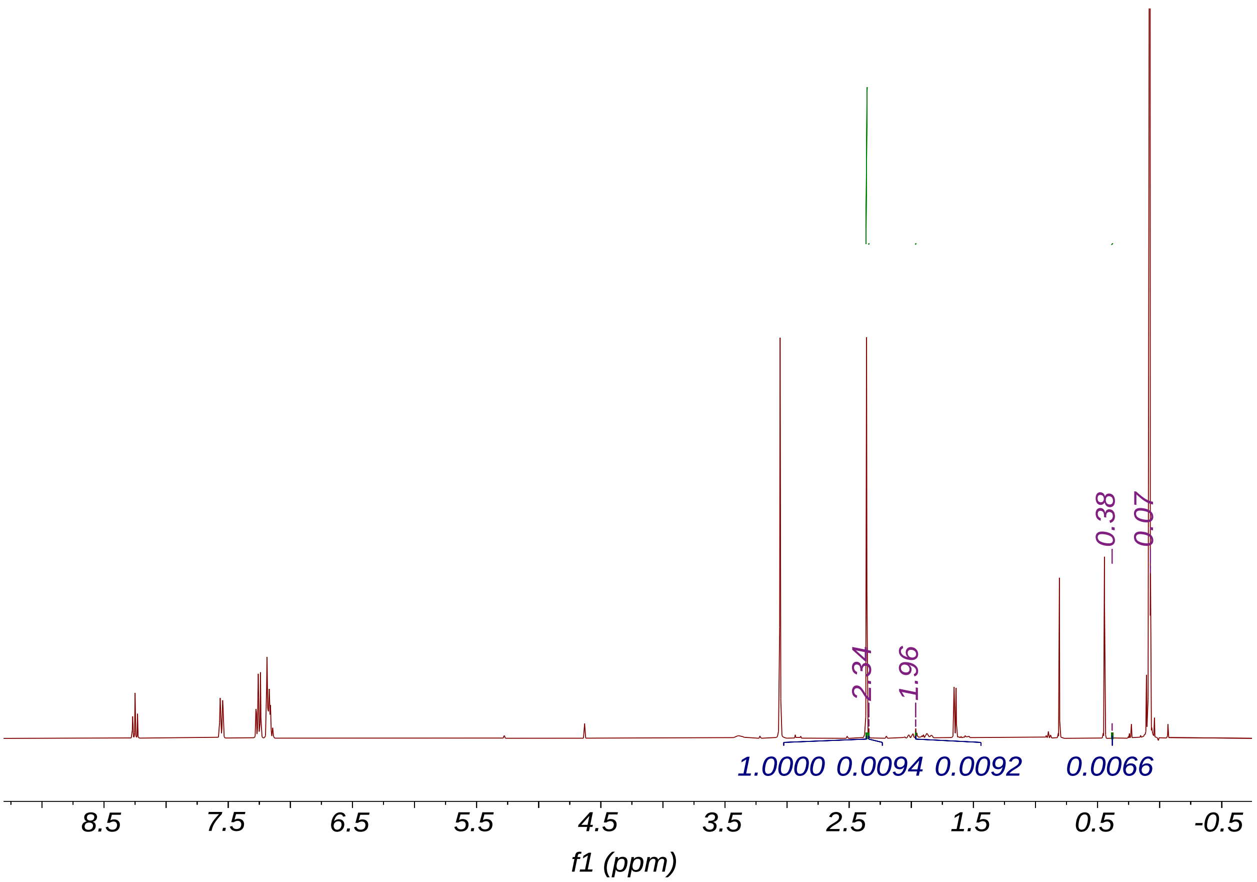

Figure S 24. ^{1}H NMR of $B(C_6F_5)_3$/2,6-Lutidine/Me_3Si-I, 20 hours at 100°C, $CDCl_3$

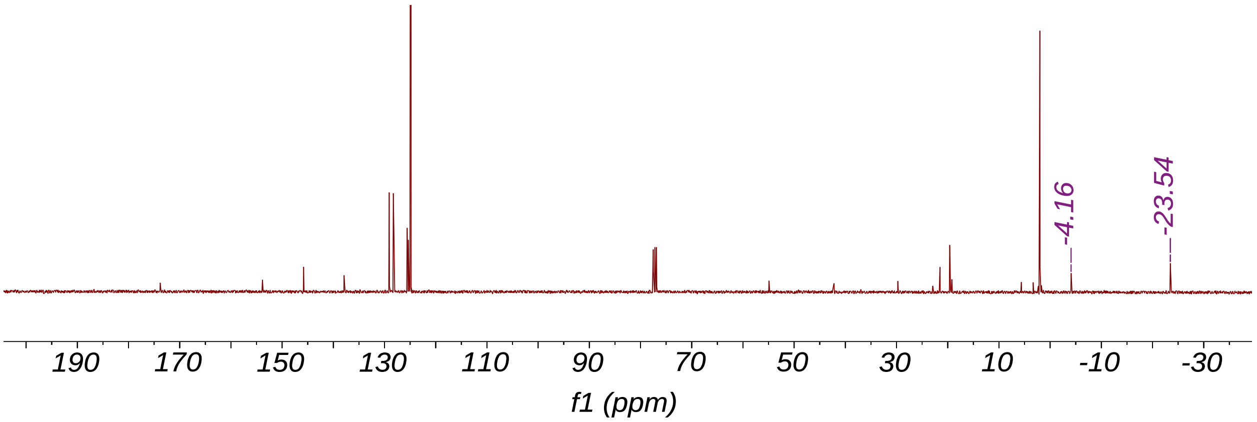

Figure S 25. $^{13}C\{^{1}H\}$ NMR of $B(C_6F_5)_3$/2,6-Lutidine/Me_3Si-I, 20 hours at 100°C, $CDCl_3$

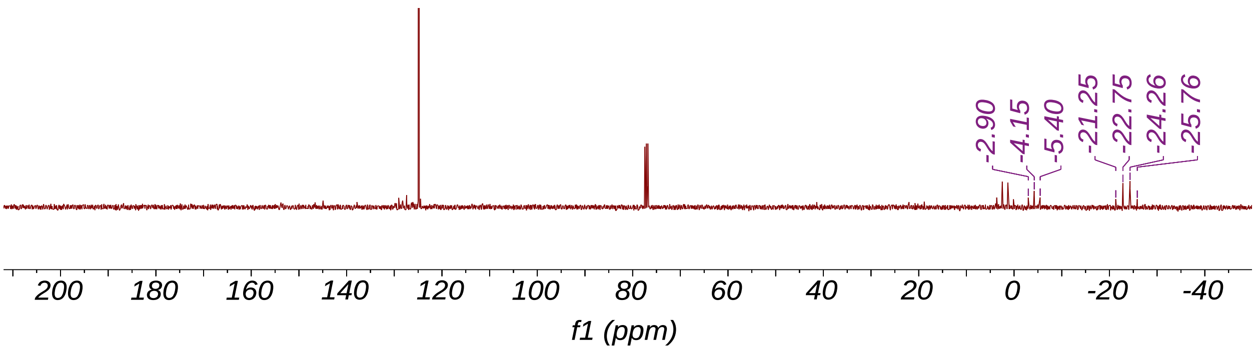

Figure S 26. ^{13}C NMR of $B(C_6F_5)_3$/2,6-Lutidine/Me_3Si-I, 20 hours at 100°C, $CDCl_3$

Entry 7: $B(C_6F_5)_3$/2,6-Lutidine/Et_3Si-I in C_6D_6

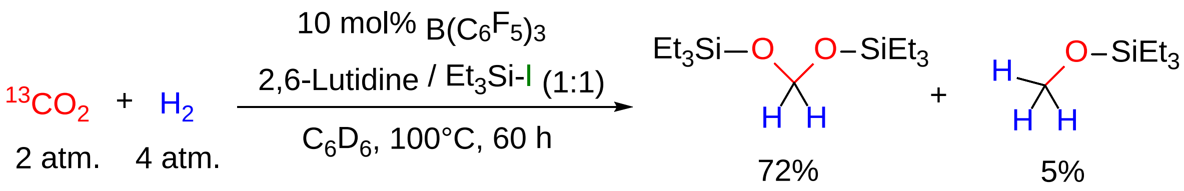

^1H NMR (600 MHz, C_6D_6) δ 5.06 (d, *J* = 161.7 Hz, $(Et_3SiO)_2{}^{13}C\underline{H}_2$), 3.31 (d, *J* = 141.0 Hz, $Et_3SiO^{13}C\underline{H}_3$).

$^{13}C\{^1H\}$ NMR (101 MHz, C_6D_6) δ 84.5 ($(Et_3SiO)_2\underline{^{13}C}H_2$), 50.5 ($Et_3SiO\underline{^{13}C}H_3$).

Overall yield = 77% at 60 hours, $(Et_3SiO)_2{}^{13}CH_2$: $Et_3SiO^{13}CH_3$= 19:1

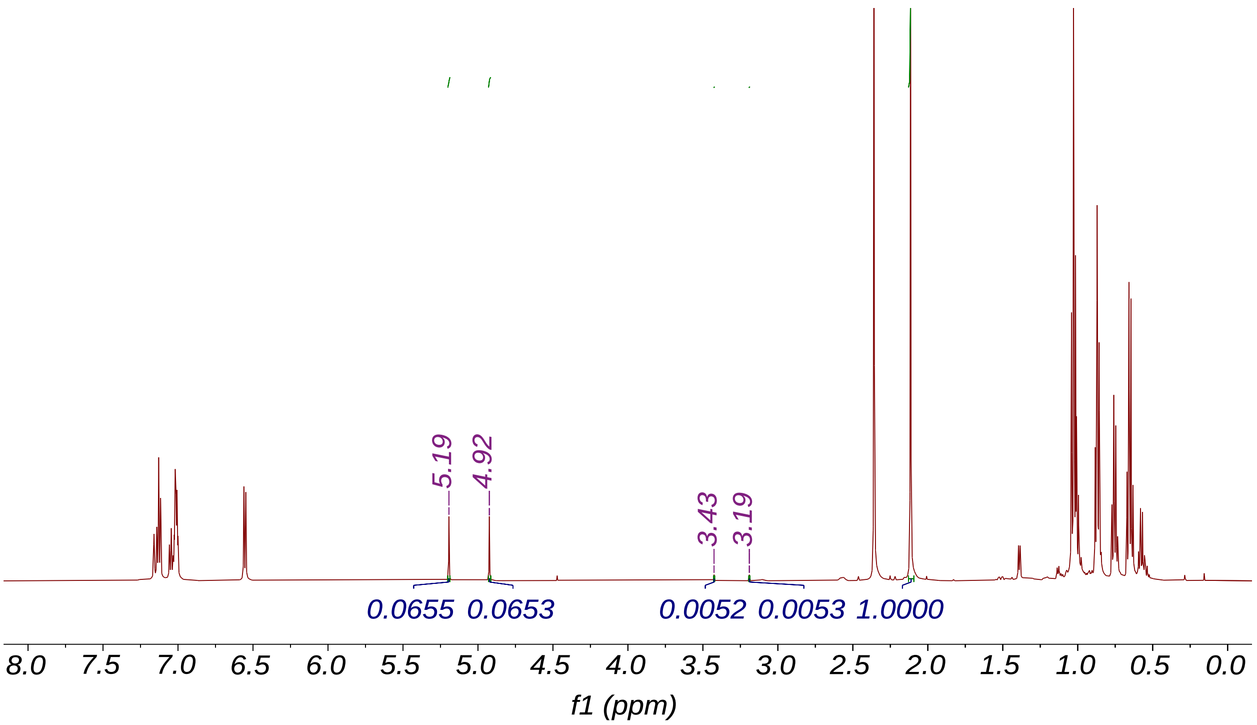

Figure S 27. ^{1}H NMR of B(C_6F_5)$_3$/2,6-Lutidine/Et$_3$Si-I, 60 hours at 100°C, C_6D_6

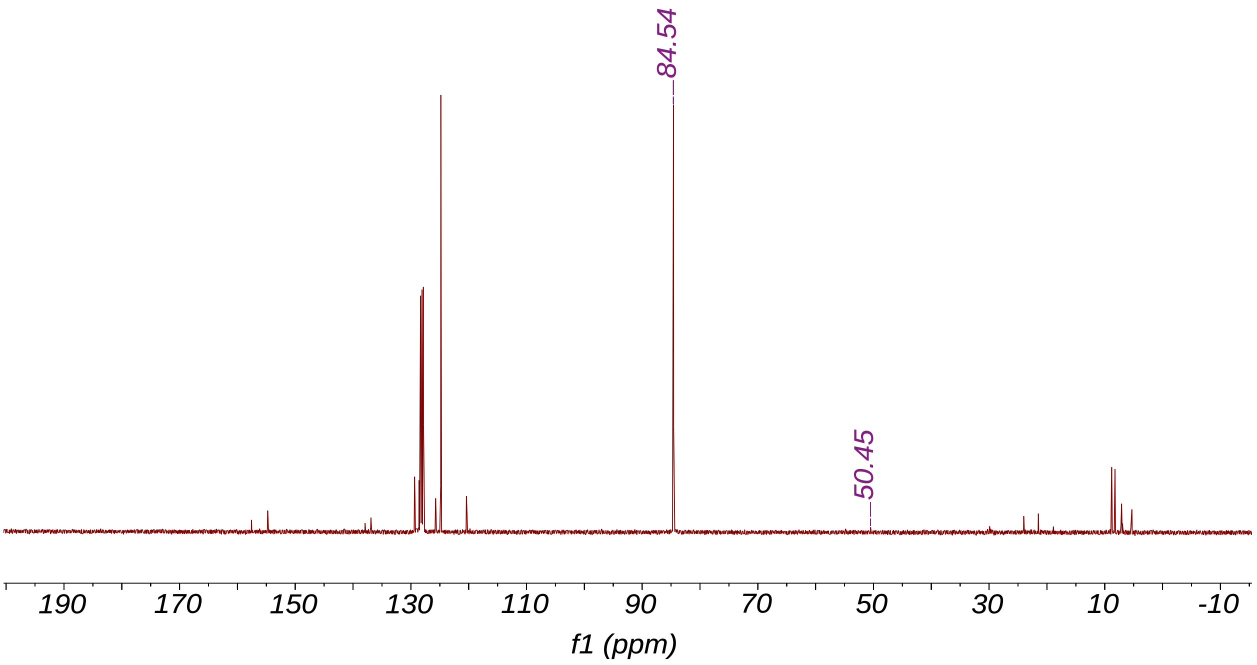

Figure S 28. ^{13}C{^{1}H} NMR of B(C_6F_5)$_3$/2,6-Lutidine/Et$_3$Si-I, 60 hours at 100°C, C_6D_6

Entry 8: B(C_6F_5)$_3$/2,6-Lutidine/Et$_3$Si-I in $CDCl_3$

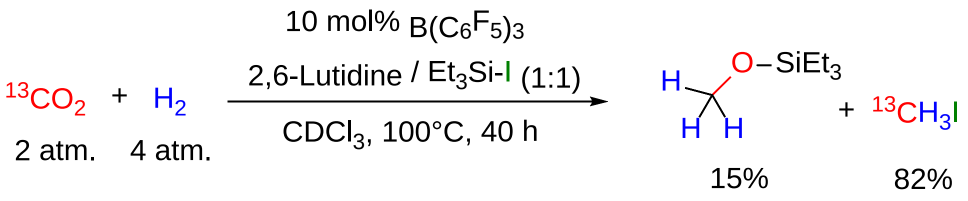

^{1}H NMR (400 MHz, $CDCl_3$) δ 3.47 (d, *J* = 141.5 Hz, $Et_3SiO^{13}C\underline{H}_3$), 2.14 (d, *J* = 151.2 Hz, $^{13}C\underline{H}_3I$).

^{13}C NMR (101 MHz, $CDCl_3$) δ 50.8 (q, *J* = 141.4 Hz, $Et_3SiO^{1}\,\underline{^{13}C}H_3$), -23.5 (q, *J* = 151.2 Hz, $\underline{^{13}C}H_3I$).

Overall yield = 97% at 40 hours, $^{13}CH_3I$: $Et_3SiO^{13}CH_3$= 5.5:1

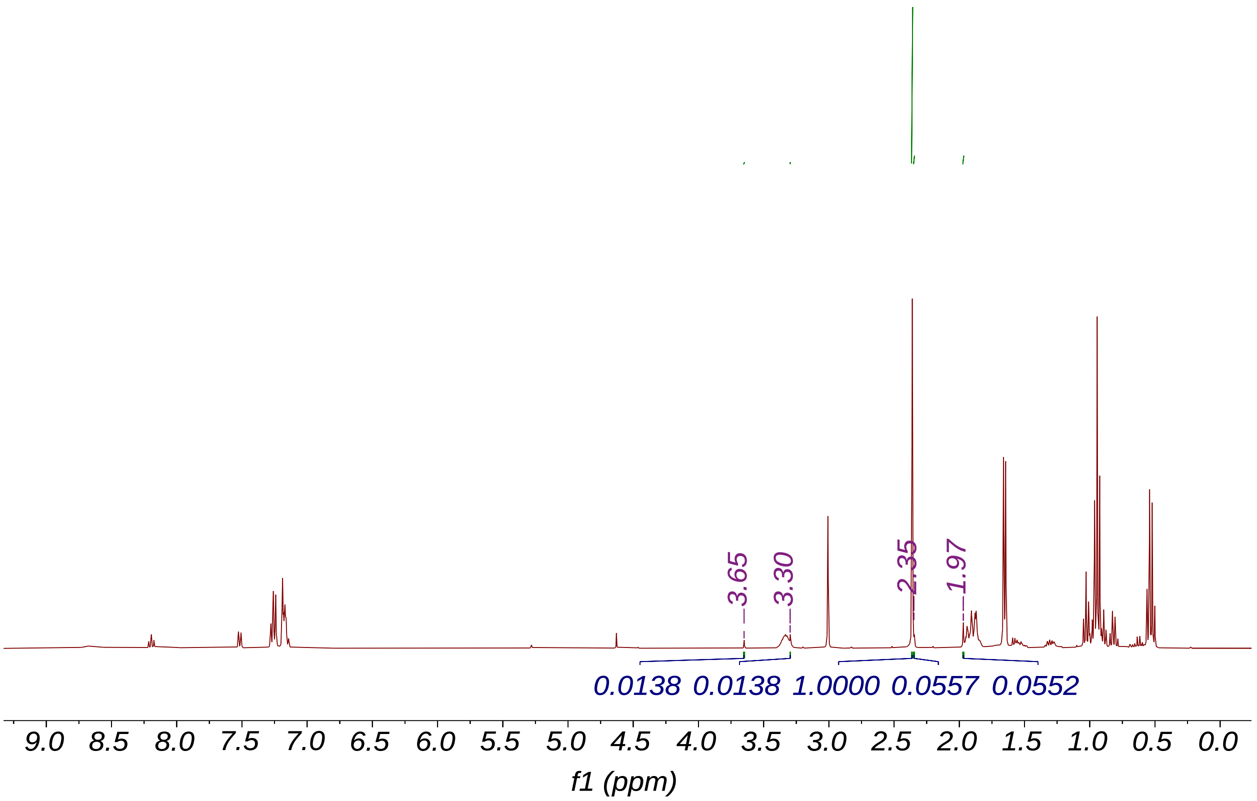

Figure S 29. ^{1}H NMR of $B(C_6F_5)_3$/2,6-Lutidine/Et_3Si-I, 40 hours at 100°C, $CDCl_3$

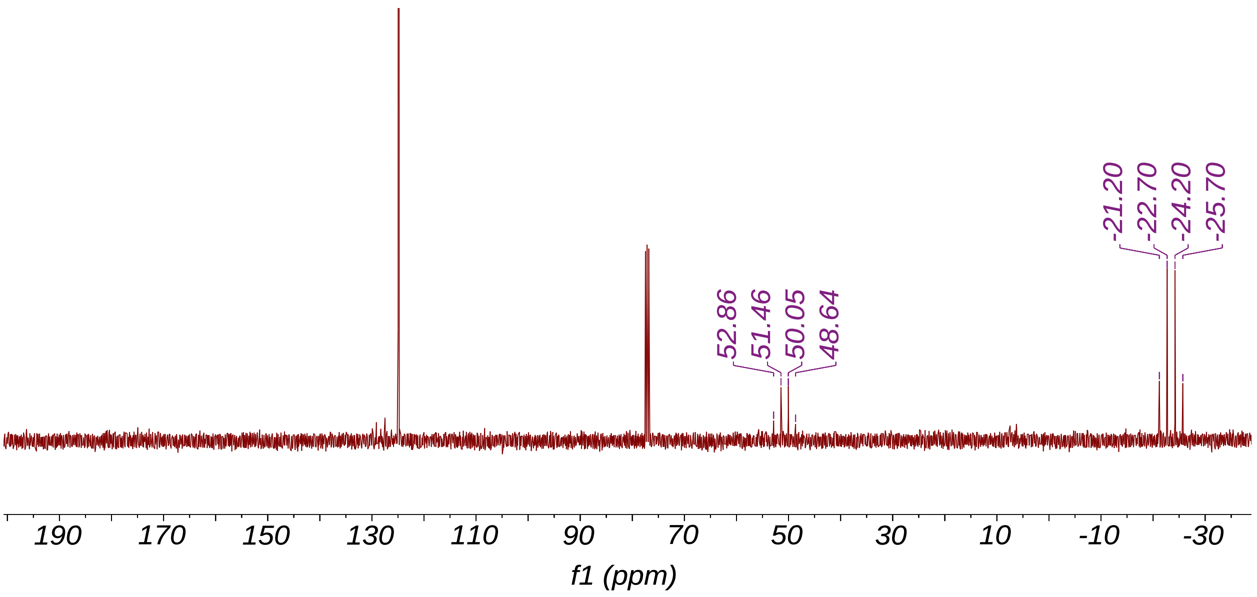

Figure S 30. ^{13}C NMR of $B(C_6F_5)_3$/2,6-Lutidine/Et_3Si-I, 40 hours at 100°C, $CDCl_3$

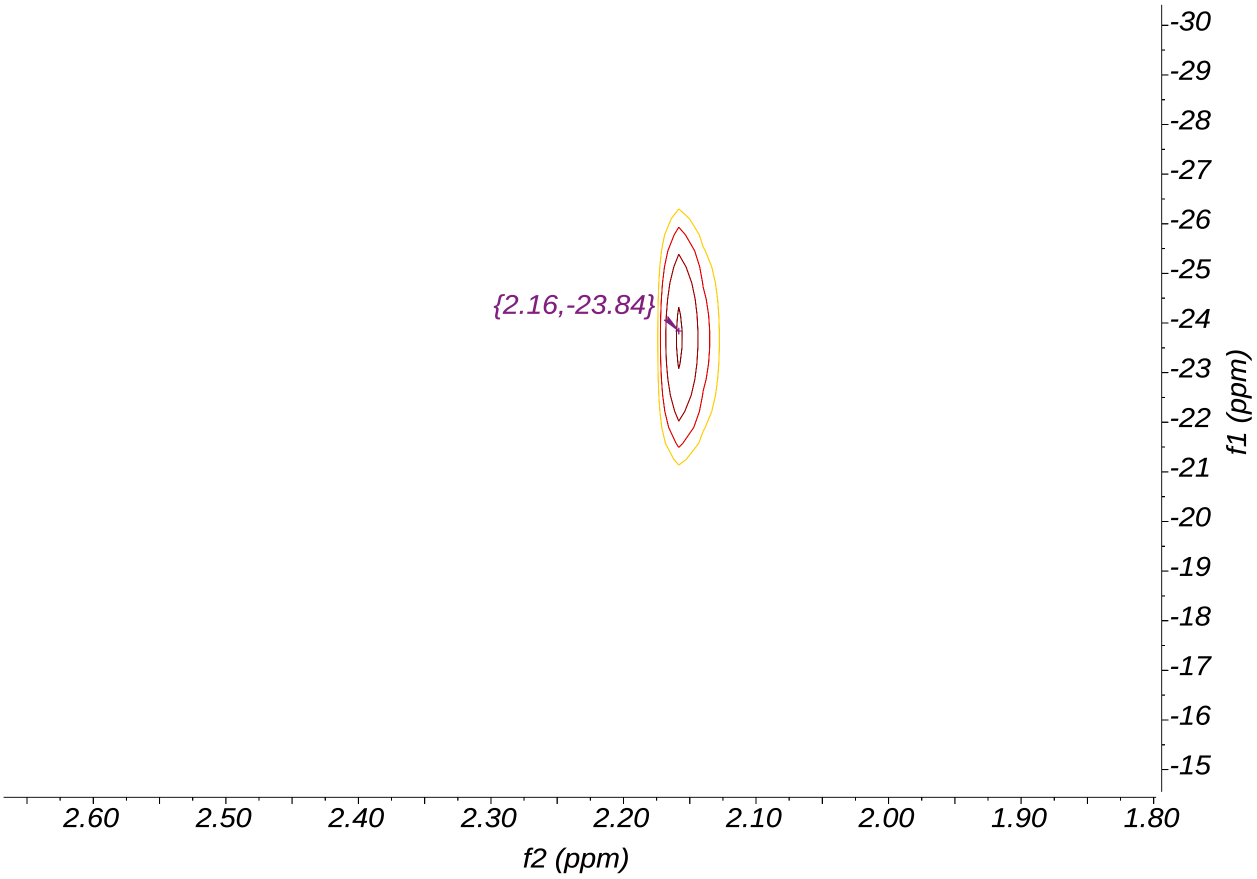

Figure S 31. HSQC spectrum of $B(C_6F_5)_3$/2,6-Lutidine/Et_3Si-I, 40 hours at 100°C, $CDCl_3$

6. $B(C_6F_5)_3$/2,4,6-Collidine FLP and halosilanes with H_2/CO_2

Entry 9: B(C_6F_5)$_3$/2,4,6-Collidine/Et_3Si-I in C_6D_6

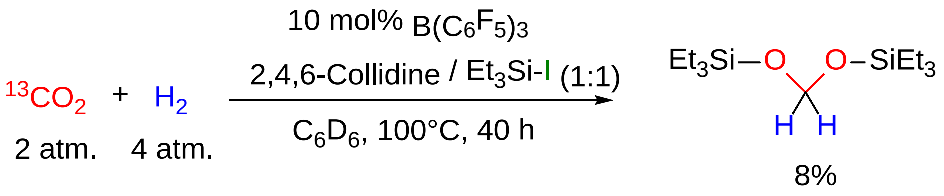

^{1}H NMR (500 MHz, C_6D_6) δ 5.06 (d, *J* = 161.7 Hz, (Et_3SiO)$_2$^{13}C*H*$_2$),

^{13}C{^{1}H} NMR (101 MHz, C_6D_6) δ 84.5 ((Et_3SiO)$_2$^{13}*C*H_2).

Overall yield = 8% at 40 hours; (Et_3SiO)$_2$^{13}CH_2

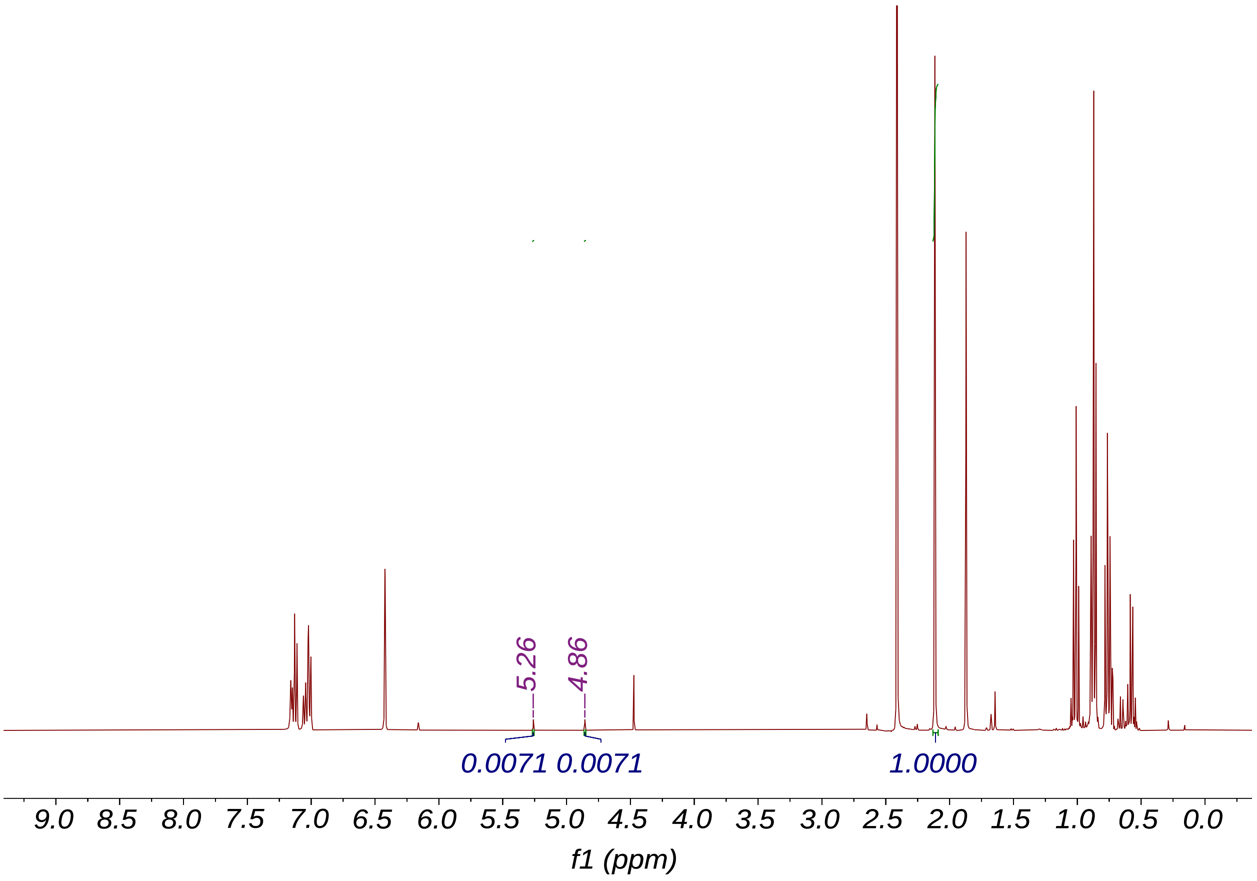

Figure S 32. ^{1}H NMR of B(C_6F_5)$_3$/2,4,6-Collidine/Et_3Si-I, 40 hours at 100°C, C_6D_6

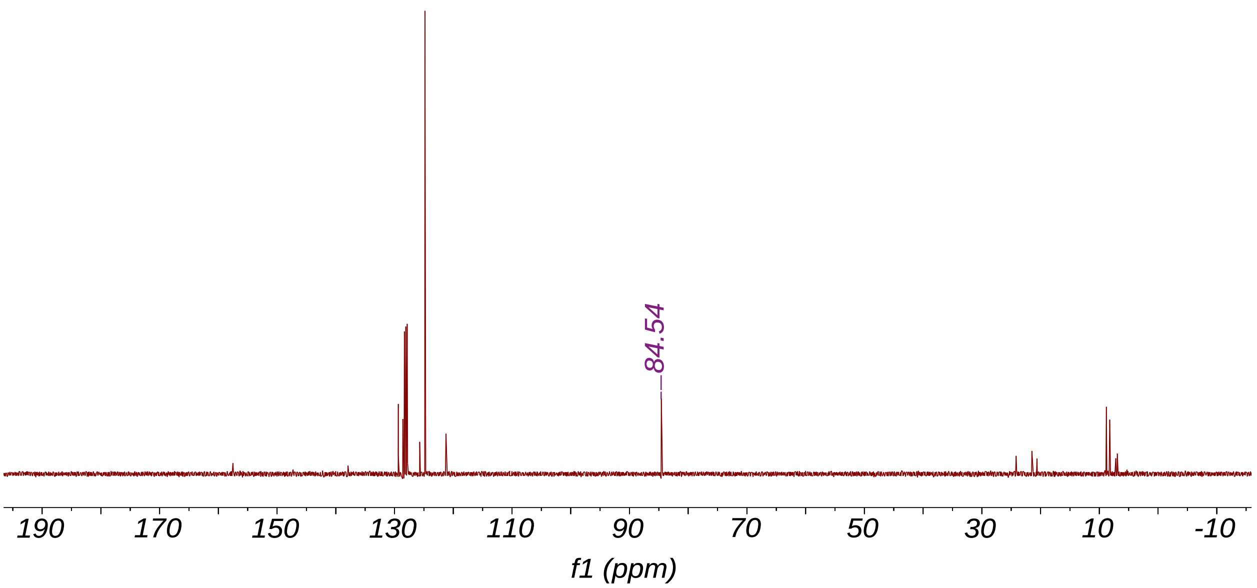

Figure S 33. $^{13}C\{^{1}H\}$ *NMR of* $B(C_6F_5)_3$*/2,4,6-Collidine/* Et_3Si*-I, 40 hours at 100°C,* C_6D_6

Entry 10: $B(C_6F_5)_3$/2,4,6-Collidine/Et_3Si-I in $CDCl_3$

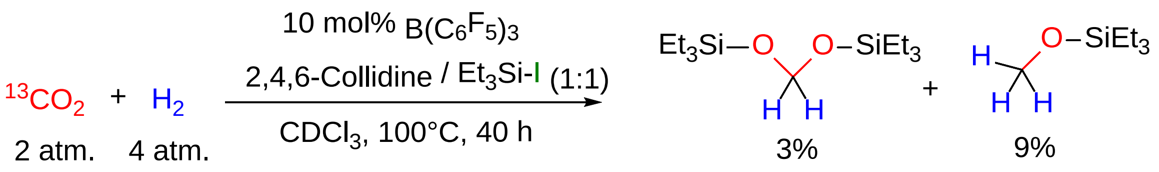

^{1}H NMR (400 MHz, $CDCl_3$) δ 5.06 (d, *J* = 161.6 Hz, $(Et_3SiO)_2{}^{13}C\underline{H}_2$), 3.47 (d, *J* = 141.4 Hz, $Et_3SiO^{13}C^{H}{}_3$).

$^{13}C\{^{1}H\}$ NMR (101 MHz, $CDCl_3$) δ 84.2 ($(Et_3SiO)_2\underline{{}^{13}C}H_2$), 50.6 ($Et_3SiO\underline{{}^{13}C}H_3$).

Overall yield = 12% at 40 hours; $Et_3SiO^{13}CH_3$: $(Et_3SiO)_2{}^{13}CH_2$= 3.4:1.

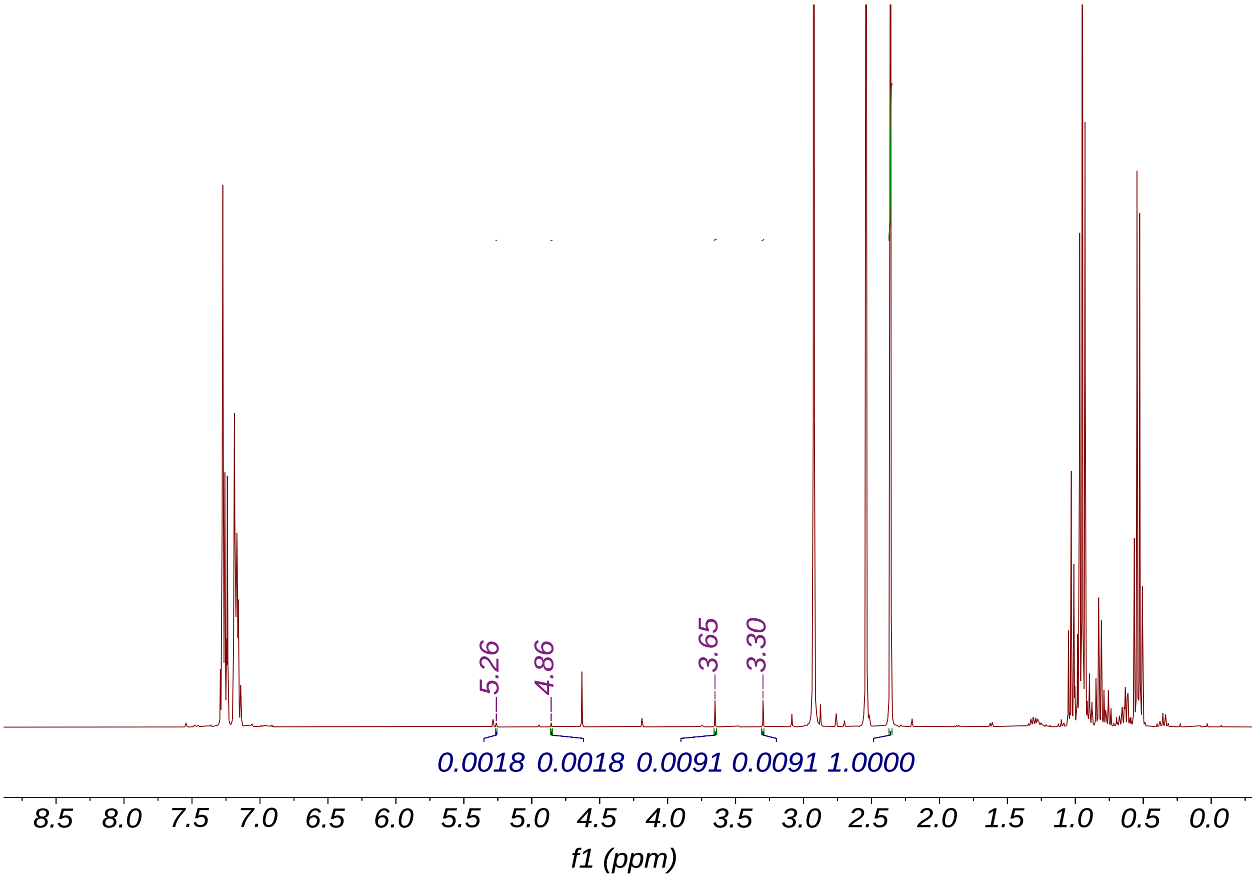

Figure S 34. ^{1}H NMR of $B(C_6F_5)_3$/2,4,6-Collidine/Et_3Si-I, 40 hours at 100°C, $CDCl_3$

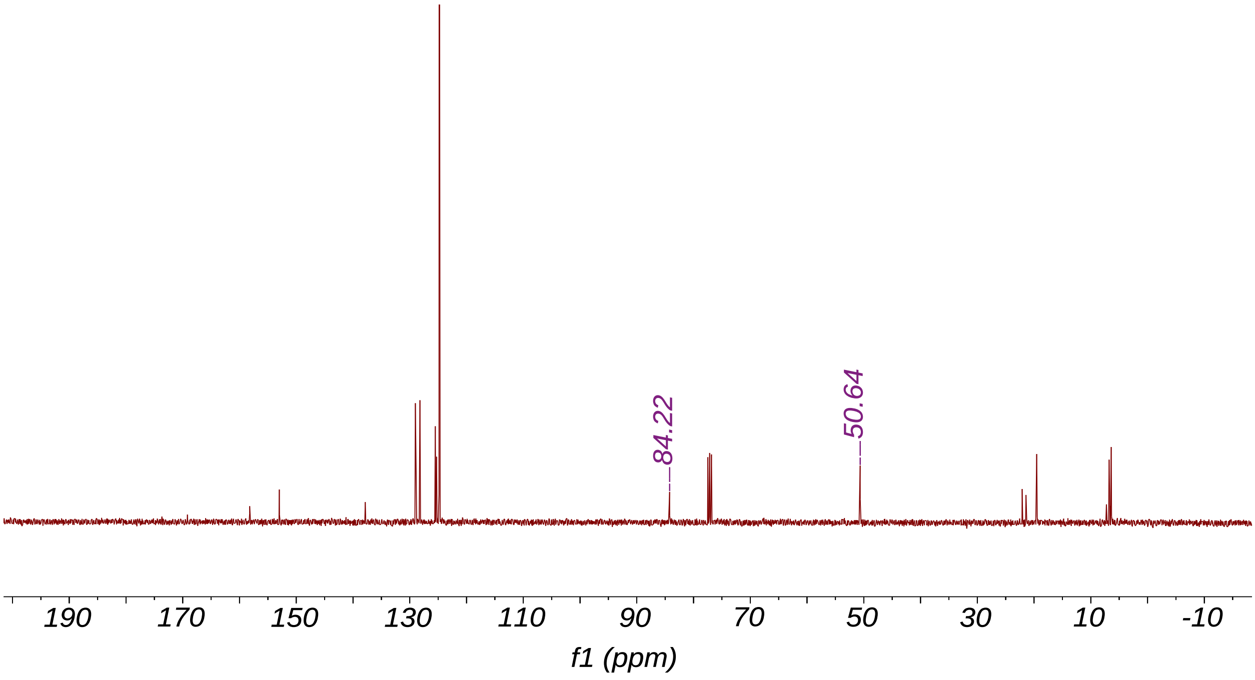

Figure S 35. $^{13}C\{^{1}H\}$ NMR of $B(C_6F_5)_3$/2,4,6-Collidine/Et_3Si-I, 40 hours at 100°C, $CDCl_3$

7. $B(C_6F_5)_3$/2,6-Lutidine FLP and halosilanes with D_2/CO_2

Reaction for 24 hours

10 mol% $B(C_6F_5)_3$ (2.6 mg, 0.0051 mmol) in 0.4 mL $CDCl_3$ was transferred to a J-young tube, followed by the addition of Et_3Si-I (8.9 µL, 0.051 mmol, 10 eq), 2,6-lutidine (6.0 µL, 0.051 mmol, 10 eq) and 10 µL toluene (internal standard). After freeze-pump-thaw degassing, the solution was kept frozen in a liquid nitrogen bath, 2 atm. $^{13}CO_2$ and 2 atm. D_2 was added to the J-young tube. After warming up back to room temperature, the J-young tube was heated in a 100°C oil bath for 24 hours. 0.8 mg [13C]Dipp-urea (^{13}C enriched 1,3-bis(2,6-diisopropylphenyl)urea (MW = 381.28 g/mol)) was added from stock solution to the crude reaction mixture as internal standard for yield determination.

Note: H/D scramble at the methyl groups of 2,6-lutidine was observed.[6]

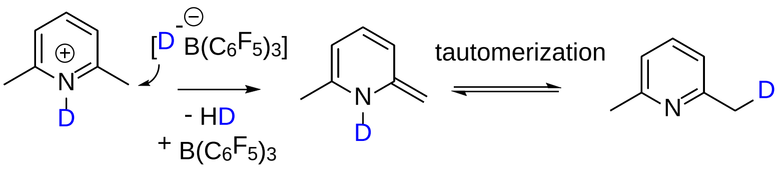

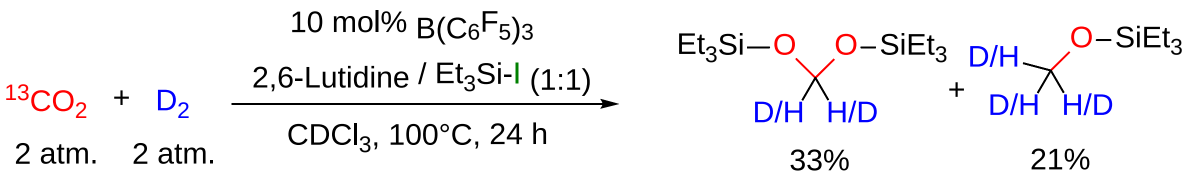

$^{13}C\{^1H\}$ NMR (126 MHz, $CDCl_3$) δ 84.3 (s, 0.68C, $(Et_3SiO)_2{}^{13}CH_2$), 84.0 (t, 2.64C, J = 24.8 Hz, $(Et_3SiO)_2{}^{13}CHD$), 83.6 (p, 0.72C, J = 24.7 Hz, $(Et_3SiO)_2{}^{13}CD_2$), 50.8 (s, 0.14C, $Et_3SiO^{13}CH_3$), 50.5 (t, 0.75C, J = 21.7 Hz, $Et_3SiO^{13}CH_2D$), 50.2 (p, 1.11C, J = 21.7 Hz, $Et_3SiO^{13}CHD_2$), 49.7 (sept, 0.54C, J = 21.6 Hz, $Et_3SiO^{13}CD_3$).

Overall yield = 54% at 24 hours, acetal: methoxy = 1.6 : 1

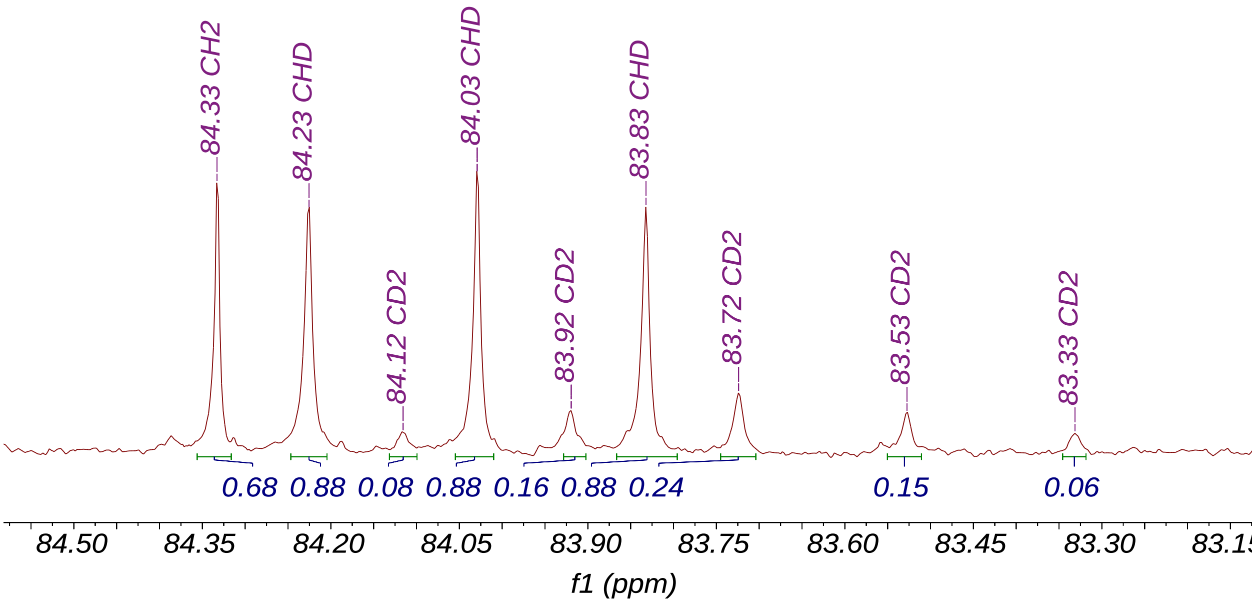

Figure S 36. $^{13}C\{^{1}H\}$ NMR spectrum, acetal species region, 24 hours at 100°C, $CDCl_3$

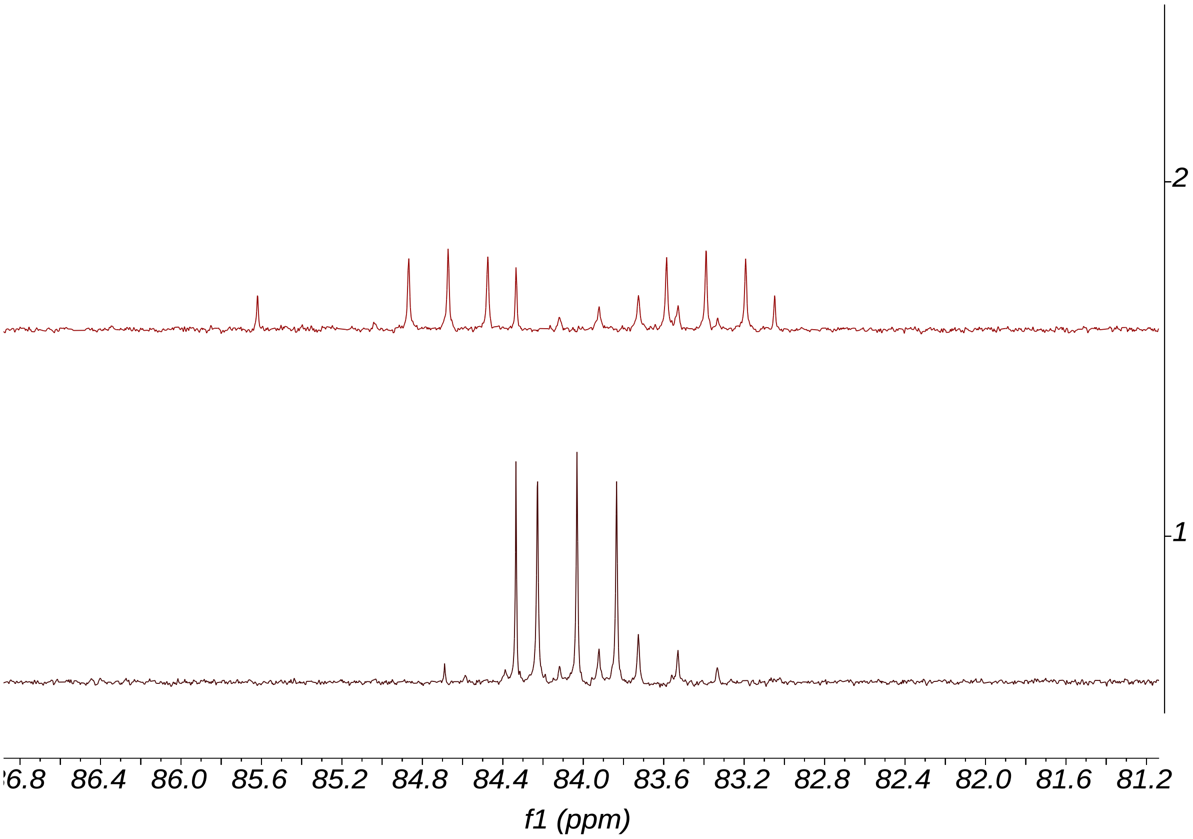

Figure S 37. $^{13}C\{^{1}H\}$ NMR (bottom) and ^{13}C NMR (top) spectra, acetal species region, 24 hours at 100°C, $CDCl_3$

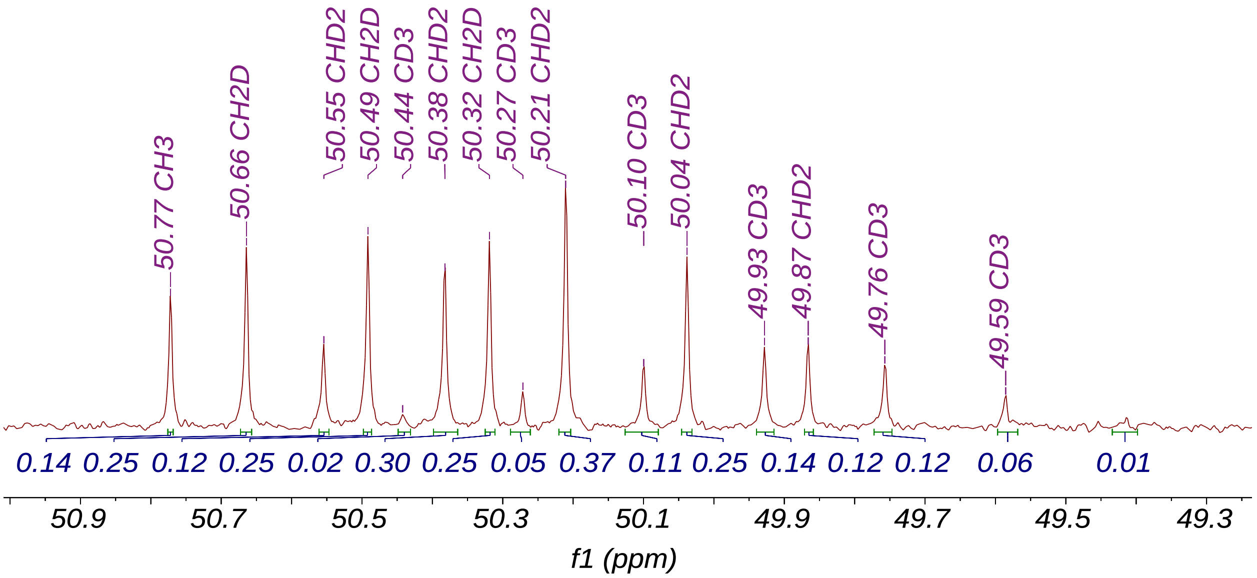

Figure S 38. $^{13}C\{^1H\}$ NMR spectrum, methoxy species region, 24 hours at 100°C, $CDCl_3$

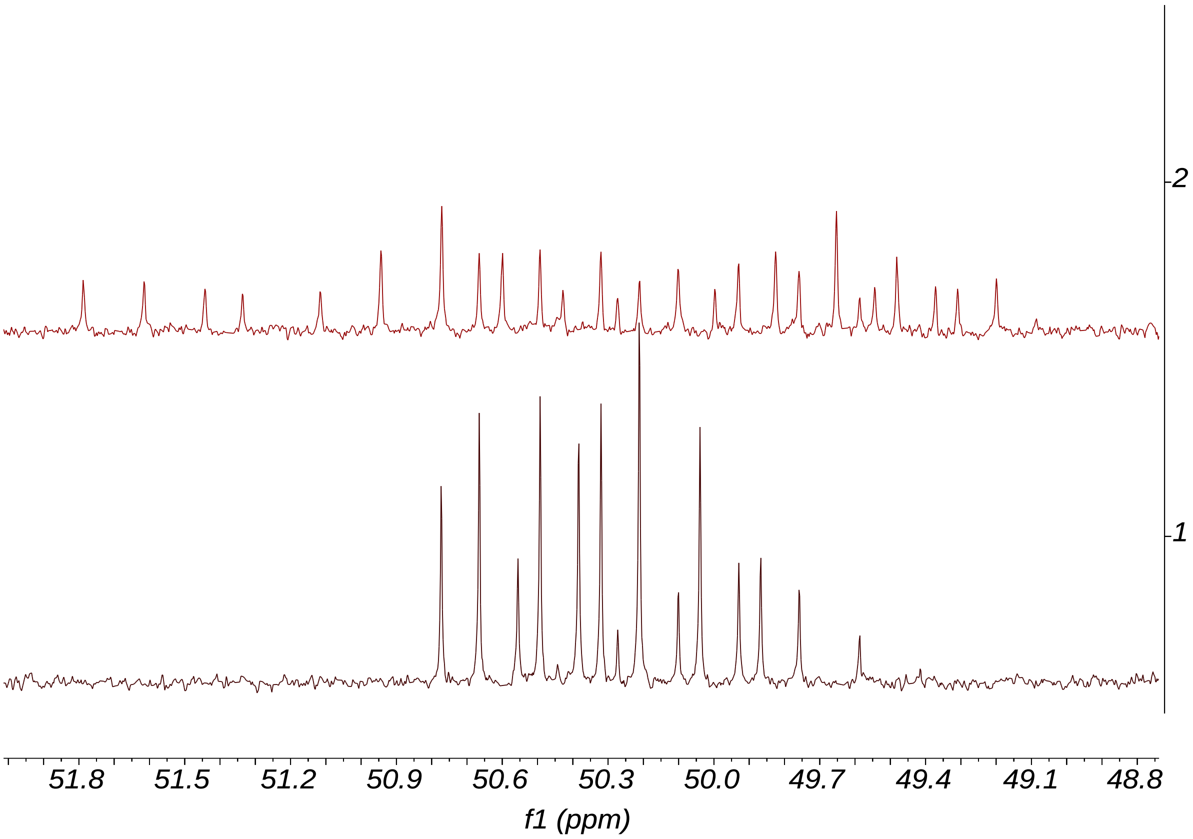

Figure S 39. $^{13}C\{^1H\}$ NMR (bottom) and ^{13}C NMR (top) spectra, methoxy species region, 24 hours at 100°C, $CDCl_3$

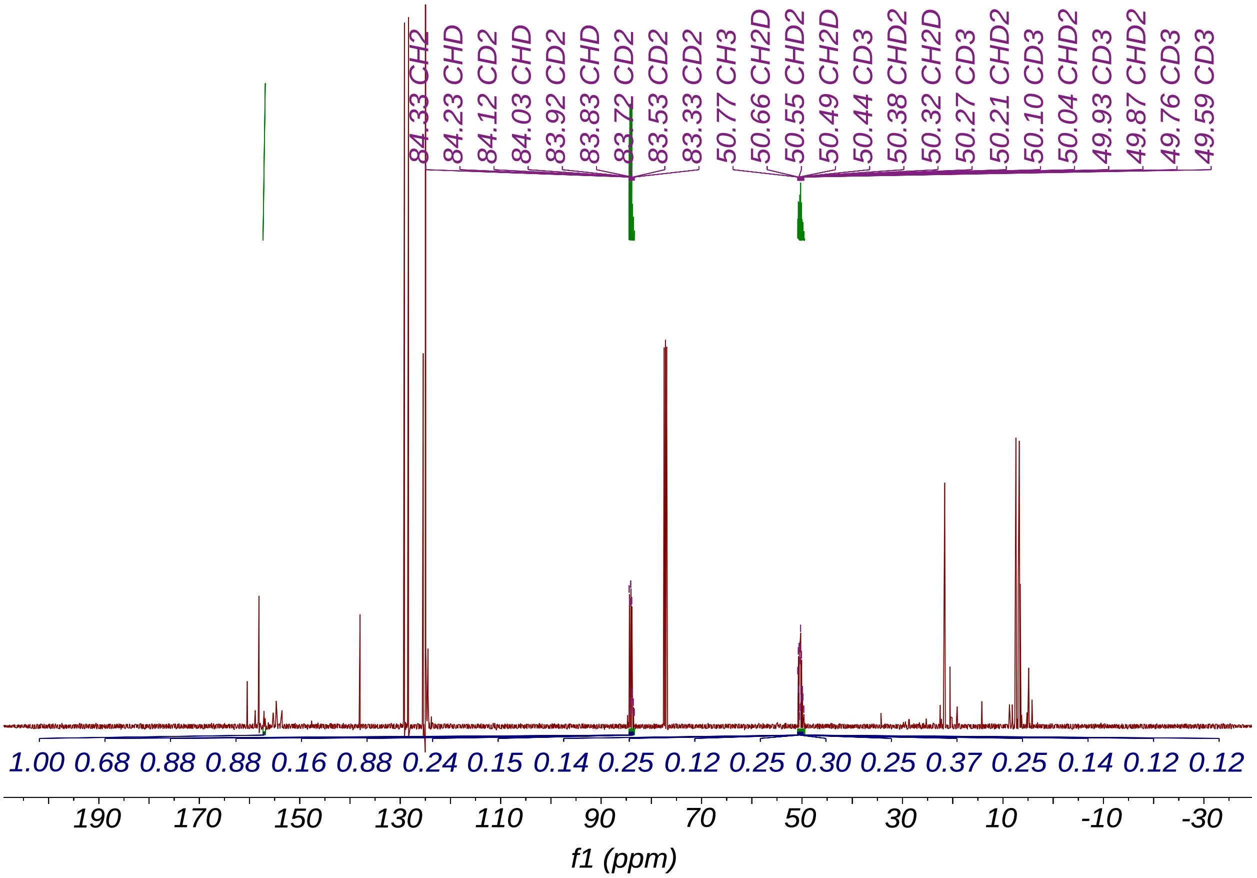

Figure S 40. $^{13}C\{^{1}H\}$ NMR spectrum of $B(C_6F_5)_3$/2,6-Lutidine/Et_3Si-I, 20 hours at 100°C, internal standard added, $CDCl_3$

Reaction for 70 hours

10 mol% $B(C_6F_5)_3$ (2.6 mg, 0.0051 mmol) in 0.4 mL $CDCl_3$ was transferred to a J-young tube, followed by the addition of Et_3Si-I (8.9 µL, 0.051 mmol, 10 eq), 2,6-lutidine (6.0 µL, 0.051 mmol, 10 eq) and 10 µL toluene (internal standard). After freeze-pump-thaw degassing, the solution was kept frozen in a liquid nitrogen bath, 2 atm. $^{13}CO_2$ and 2 atm. D_2 was added to the J-young tube. After warming up back to room temperature, the J-young tube was heated in a 100°C oil bath for 70 hours. 5.2 mg [13C]Dipp-urea (^{13}C enriched 1,3-bis(2,6-diisopropylphenyl)urea (MW = 381.28 g/mol)) was added to the crude reaction mixture as internal standard for yield determination.

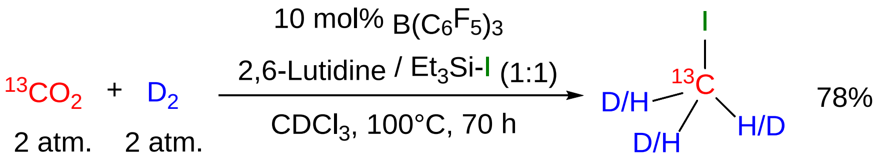

$^{13}C\{^{1}H\}$ NMR (126 MHz, $CDCl_3$) δ -23.39 (s, 1C, $^{13}CH_3I$), -23.41 (t, 4.74C, J = 23.4 Hz, $^{13}CDH_2I$), -23.44 (p, 4.65C, J = 23.2 Hz, $^{13}CD_2HI$), -23.47 (septet, 3.62C, J = 23.1 Hz, $^{13}CD_3I$).

Overall yield = 78% at 70 hours, iodomethane only

The total integration of each isotopologues is derived from the integration of their central resonance based on the 1:1:1 ratio of the triplet resonance of $^{13}CDH_2I$; 1:2:3:2:1 ratio of the pentet resonance of $^{13}CD_2HI$ and the 1:3:6:7:6:3:1 ratio of the septet resonance of $^{13}CD_3I$.

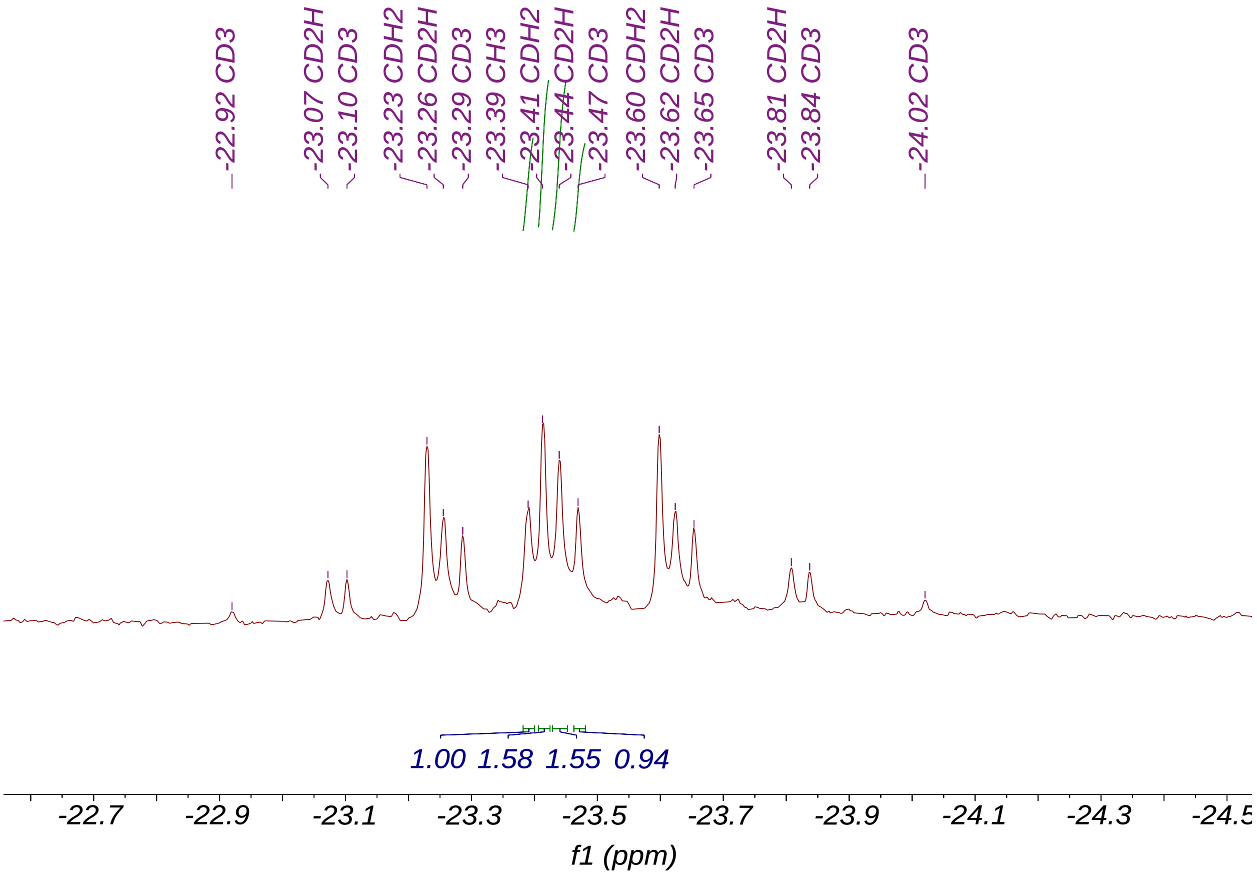

Figure S 41. $^{13}C\{^1H\}$ NMR spectrum, iodomethane region, 70 hours at 100°C, $CDCl_3$

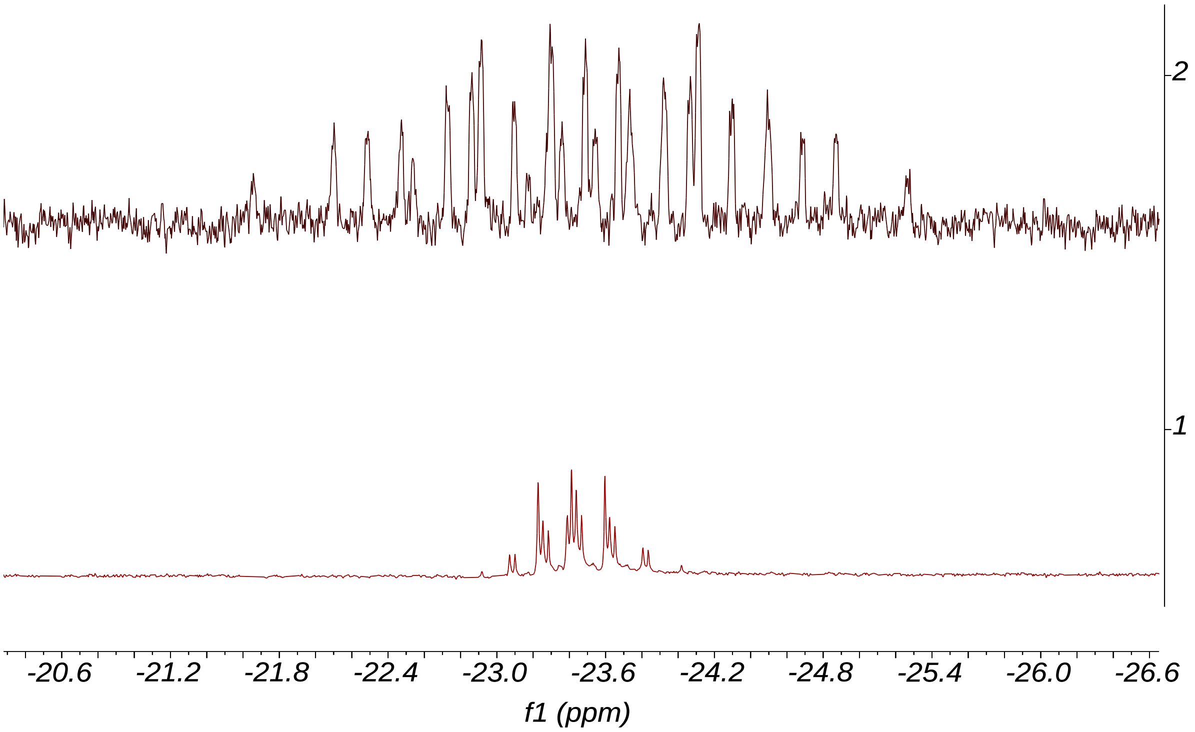

Figure S 42. $^{13}C\{^1H\}$ NMR (bottom) and ^{13}C NMR (top) spectra, iodomethane region, 70 hours at 100°C, $CDCl_3$

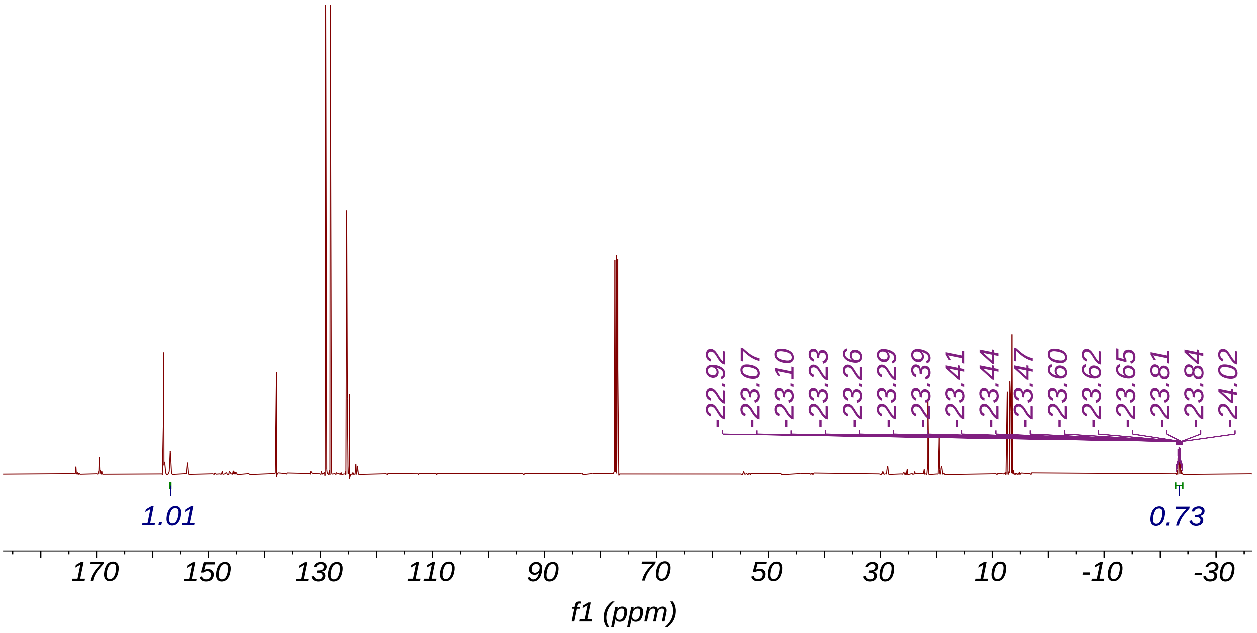

Figure S 43. $^{13}C\{^1H\}$ NMR spectrum of $B(C_6F_5)_3$/2,6-Lutidine/Et_3Si-I, 70 hours at 100°C, internal standard added, $CDCl_3$

8. Experimental References

[1] S. D. Tran, T. A. Tronic, W. Kaminsky, D. Michael Heinekey, J. M. Mayer, *Inorganica Chimica Acta* **2011**, *369*, 126-132.

[2] J. Chen, L. Falivene, L. Caporaso, L. Cavallo, E. Y. X. Chen, *Journal of the American Chemical Society* **2016**, *138*, 5321-5333.

[3] aC. Altona, J. H. Ippel, A. J. A. W. Hoekzema, C. Erkelens, M. Groesbeek, L. A. Donders, *Magnetic Resonance in Chemistry* **1989**, *27*, 564-576; bJ. John, E. Gravel, A. Hagège, H. Li, T. Gacoin, E. Doris, *Angewandte Chemie International Edition* **2011**, *50*, 7533-7536; cM. Ahrens, G. Scholz, T. Braun, E. Kemnitz, *Angewandte Chemie International Edition* **2013**, *52*, 5328-5332.

[4] T. Mahdi, J. N. del Castillo, D. W. Stephan, *Organometallics* **2013**, *32*, 1971-1978.

[5] N. A. Darwish, K. A. M. Gasem, R. L. Robinson, Jr., *Journal of Chemical & Engineering Data* **1994**, *39*, 781-784.

[6] X. Zhang, L. Wang, Q. Wang, F. Ge, X. Wang, *ChemistrySelect* **2019**, *4*, 8655-8660.

9. DFT computational Details:

The quantum chemical DFT calculations have been performed with the TURBOMOLE 7.4 suite of programs[1] The structures are fully optimized at the TPSS-D3/def2-TZVP + COSMO level of theory, which combines the TPSS meta-GGA density functional[2] with the BJ-damped DFT-D3 dispersion correction[3, 4] and the def2-TZVP basis set,[5, 6] using the Conductor-like Screening Model (COSMO) continuum solvation model[7] for $CHCl_3$ solvent (dielectric constant $\varepsilon = 4.8$ and solvent diameter $R_{solv} = 3.17$ Å). The density-fitting RI-J approach[5, 8, 9] is used to accelerate the geometry optimization and numerical harmonic frequency calculations[10] in solution. The optimized structures are characterized by frequency analysis to identify the nature of located stationary points (no imaginary frequency for true minima and only one imaginary frequency for transition state) and to provide thermal corrections (at 298.15 K and 1 atm) according to the modified ideal gas–rigid rotor–harmonic oscillator model.[11] This choice of dispersion-corrected meta-GGA functional makes the efficient exploration of all potential reaction paths possible.

The final solvation free energies in $CHCl_3$ are computed with the COSMO-RS solvation model[12] (parameter file: BP_TZVP_C30_1601.ctd) using the COSMOtherm program package[13 on the above TPSS-D3 optimized structures, and corrected by +1.89 kcal·mol^{-1} to account for higher reference solute concentration of 1 mol·L^{-1} usually used in solution. To check the effects of the chosen DFT functional on the reaction energies and barriers, single-point calculations at the meta-GGA TPSS-D3[2] and hybrid-meta-GGA PW6B95-D3[14] levels are performed using a larger def2-QZVP basis set.[6, 15] The final reaction Gibbs free energies (ΔG) are determined from the electronic single-point energies plus TPSS-D3 thermal corrections and COSMO-RS solvation free energies. The computed relative free energies from both DFT functionals are mostly in very good overall agreement of 0.1 ± 2.2 kcal/mol (average ± standard deviations), while as can be expected, about 2.8 ± 2.2 kcal/mol (average ± standard deviations) higher reaction barriers are observed at the PW6B95-D3 level. In our discussion, higher-level PW6B95-D3 Gibbs free energies (in kcal/mol, at 298.15 K and 1 mol/L concentration) will be used in our discussion unless specified otherwise. The applied DFT methods in combination with the large AO basis set provide usually accurate electronic energies leading to errors for chemical energies (including barriers) on the order of typically 1-2 kcal/mol. This has been tested thoroughly for the huge data base GMTKN55[16] which is the common standard in the field of DFT benchmarking.

Table S1. TPSS-D3/def2-TZVP + COSMO computed imaginary frequency (ImF), zero-point energies (ZPE), gas-phase enthalpic (Hc) and Gibbs free-energy (Gc) corrections; the COSMO-RS computed solvation enthalpic (Hsol) and Gibbs free-energy (Gsol) corrections in THF solution; TPSS-D3/def2-QZVP and PW6B95-D3/def2-QZVP single-point energies (TPSS-D3 and PW6B95-D3); the total PW6B95-D3 free energies GP; the relative electronic energies (ΔET and ΔEP) and Gibbs free-energies (ΔGT and ΔGP) at the TPSS-D3 and PW6B95-D3 levels.

Reactions	Im	ZPE	Hc	Gc	Hsol	Gsol	TPSS-D3	PW6B95-D3	G_P	ΔE_T	ΔE_P	ΔG_P	ΔG_T
in $CHCl_3$ solution	cm^{-1}	kcal	kcal	kcal	kcal	kcal	E_h	E_h	E_h	kcal	kcal	kcal	kcal
Reaction of CO_2 with separated FLP of Lutidine (Lut) and $B(C_6F_5)_3$ is 11.5 kcal/mol endergonic, thus thermdynamically not favorable.													
Lut + $B(C_6F_5)_3$ + CO_2	0	191.46	217.36	126.20	-31.12	-19.87	-2725.46662	-2728.25847	-2728.07999	0.00	0.00	0.00	0.00
$LutCOOB(C_6F_5)_3$	0	194.14	219.60	154.11	-28.95	-22.89	-2725.47922	-2728.27381	-2728.06170	-7.91	-9.63	11.47	13.19
Nucleophilic replacement of Lutidine with SiMe3I is 5.1 kcal/mol endergonic to release iodide anion.													
$SiMe_3I$ + Lut	0	159.64	170.43	120.40	-23.53	-13.58	-1034.18866	-1035.19635	-1035.02010	0.00	0.00	0.00	0.00
$LutSiMe_3^+$ + I^-	0	162.28	172.91	128.18	-140.66	-111.92	-1034.03690	-1035.04390	-1035.01197	95.23	95.66	5.11	4.68
*H_2 activation with the Lut.$B(C_6F_5)_3$ adduct is facile to form reductive hydroborate salt [LutH][$HB(C_6F_5)_3$] (**A**)*													
H_2 + Lut.$B(C_6F_5)_3$	0	192.29	217.85	146.91	-23.51	-17.21	-2537.96701	-2540.56719	-2540.35448	0.00	0.00	0.00	0.00
Lut + H_2 + $B(C_6F_5)_3$	0	190.63	216.37	131.15	-28.74	-18.09	-2537.94178	-2540.54054	-2540.35132	15.83	16.73	1.99	1.09
**TS1**	47i	193.52	218.62	152.58	-21.10	-16.52	-2537.94828	-2540.54577	-2540.32593	11.75	13.44	17.91	16.23
$HB(C_6F_5)_3^-$ + $LutH^+$	0	198.18	222.26	144.72	-95.92	-87.47	-2537.86104	-2540.46277	-2540.36550	-9.30	-9.65	-8.01	-7.65
**A**	0	198.08	222.91	157.79	-29.89	-24.55	-2537.98183	-2540.58258	-2540.36724	66.49	65.53	-6.91	-5.95
*..forming salt [LutH][$B(C_6F_5)_3OCHO$] (**B**) after CO_2 addition*													
**A** + CO_2	0	205.24	232.31	151.96	-32.93	-25.26	-2726.68726	-2729.47646	-2729.26852	0.00	0.00	0.00	0.00
**A**.CO_2	0	204.92	232.92	160.75	-33.94	-28.28	-2726.68327	-2729.47488	-2729.26076	2.50	0.99	4.87	6.38
**TS2**	254i	202.93	230.40	159.14	-31.80	-25.64	-2726.67019	-2729.45413	-2729.23836	10.71	14.01	18.92	15.62
$\mathbf{B}^-$ + $LutH^+$	0	207.35	233.35	151.74	-106.97	-92.42	-2726.57938	-2729.37392	-2729.27336	67.70	64.34	-3.04	0.31
**B**	0	206.94	233.96	164.54	-31.69	-24.32	-2726.70895	-2729.50336	-2729.27688	-13.61	-16.88	-5.25	-1.98
*With more basic 2,4,6-collidine (Col), H_2 activation with the Col.$B(C_6F_5)_3$ adduct is 1.0 kcal/mol more favorable to form [ColH][$HB(C_6F_5)_3$] (**cA**)*													
Col.$B(C_6F_5)_3$ + H_2	0	208.82	235.76	161.97	-24.59	-18.02	-2577.31441	-2579.95651	-2579.72109	0.00	0.00	0.00	0.00
$B(C_6F_5)_3$ + Col + H_2	0	208.13	234.67	147.34	-30.12	-18.99	-2577.28672	-2579.92716	-2579.71358	17.37	18.42	4.71	3.66
**cTS1**	80i	208.89	235.34	166.83	-21.90	-17.02	-2577.29670	-2579.93590	-2579.69414	11.11	12.93	16.91	15.08
**cA**	0	215.15	241.02	173.79	-30.77	-25.19	-2577.32989	-2579.97247	-2579.73265	-9.71	-10.01	-7.25	-6.95
$ColH^+$ + $\mathbf{A}^-$	0	215.65	240.55	160.87	-95.09	-86.41	-2577.21183	-2579.85540	-2579.73072	64.37	63.45	-6.04	-5.12

*..followed by kinetically less favorable CO_2 reduction with hydroborate **cA**.*													
**cA** + CO_2	0	222.31	250.42	167.97	-33.81	-25.90	-2766.03531	-2768.86634	-2768.63392	0.00	0.00	0.00	0.00
**cTS2**	0	220.74	249.45	176.02	-32.92	-26.03	-2766.01840	-2768.84434	-2768.60231	10.62	13.81	19.84	16.65
**cB**	0	224.19	252.12	181.07	-32.44	-24.81	-2766.05679	-2768.89293	-2768.64090	-13.48	-16.68	-4.38	-1.17
$ColH^+$ + $\mathbf{B}^-$	0	224.82	251.63	167.89	-106.14	-91.37	-2765.93016	-2768.76655	-2768.63858	65.98	62.62	-2.92	0.44
Silane SiMe3H formation via the reduction of $SiMe_3I$ with [LutH][HB($C_6F5)_3$] salt is 10.1 kcal/mol endergonic thus unlikely.													
**A** + $SiMe_3I$	0	267.96	298.63	207.26	-40.06	-30.45	-3245.03164	-3248.28837	-3248.00057	0.00	0.00	0.00	0.00
$LutH^+$.$ISiMe_3$.$\mathbf{A}^-$	0	269.57	300.64	223.33	-42.20	-34.40	-3245.02633	-3248.28414	-3247.98005	3.33	2.65	12.87	13.55
LutHI.$SiMe_3H$.B(C_6F_5)$_3$	0	268.32	299.51	221.14	-45.67	-32.71	-3245.02178	-3248.27615	-3247.97286	6.18	7.67	17.39	15.91
LutHI + $SiMe_3H$ + B(C_6F_5)$_3$	0	267.92	297.67	195.00	-59.89	-40.43	-3244.98693	-3248.23989	-3247.98453	28.05	30.42	10.07	7.70
*Facile nucleophilic replacement of Bf_3OCHO- anion with $SiMe_3I$ to form adduct **C** and salt LutHI*													
**B** + $SiMe_3I$	0	276.82	309.68	214.02	-41.86	-30.22	-3433.75876	-3437.20915	-3436.91021	0.00	0.00	0.00	0.00
$\mathbf{B}^-$.$SiMe_3I$ + $LutH^+$	0	277.78	310.54	214.59	-107.33	-92.82	-3433.64372	-3437.09493	-3436.89486	72.19	71.67	9.64	10.15
$\mathbf{TS3}^-$	46i	277.57	310.01	214.57	-104.32	-91.32	-3433.64093	-3437.08991	-3436.88748	73.94	74.82	14.27	13.39
**C** + LutHI	0	279.41	311.07	216.88	-61.16	-42.85	-3433.74778	-3437.19619	-3436.91283	6.89	8.13	-1.64	-2.89
*..with $SiMe_3$OCHO (**D**) being 3.9 kcal/mol bound to B(C_6F_5)$_3$ within adduct **C***													
**D** + B(C_6F_5)$_3$	0	178.96	204.39	124.48	-25.33	-16.76	-2808.35525	-2811.15657	-2810.97889	0.00	0.00	0.00	0.00
**C**	0	180.26	205.82	139.46	-20.58	-15.84	-2808.38096	-2811.18512	-2810.98511	-16.13	-17.91	-3.90	-2.12
*D / B(C_6F_5)$_3$ as FLP for H_2-activation encounter a sizeable barrier of 23.6 kcal/mol (via **TS4a**)*													
**C** + H_2	0	186.60	214.23	138.59	-21.24	-14.77	-2809.56155	-2812.36106	-2812.15772	0.00	0.00	0.00	0.00
**TS4a**	89i	187.41	214.35	144.41	-20.61	-15.66	-2809.53359	-2812.32827	-2812.12008	17.55	20.58	23.62	20.59
$\mathbf{D}H^+\mathbf{A}^-$	0	190.55	217.50	147.61	-26.94	-21.45	-2809.53212	-2812.33005	-2812.12599	18.47	19.46	19.91	18.92
*Further reaction of **D** with $SiMe_3I$ and then HB(C_6F_5)$_3^-$ is kinectially very facile and -13.3 kcal/mol exergonic*													
**D** + $SiMe_3I$	0	154.31	166.86	112.86	-20.78	-11.18	-1305.78272	-1306.98833	-1306.82028	0.00	0.00	0.00	0.00
**D**.$SiMe_3I$	0	154.76	168.14	125.20	-20.33	-12.23	-1305.78429	-1306.98947	-1306.80643	-0.99	-0.72	8.69	8.42
**TS4**	23i	153.90	167.46	123.74	-23.95	-15.81	-1305.77246	-1306.97635	-1306.80136	6.44	7.52	11.88	10.80
$\mathbf{E}^+$ + I^-	0	156.36	168.90	120.15	-139.36	-110.34	-1305.62266	-1306.82965	-1306.80801	100.44	99.57	7.70	8.57
**D** + $SiMe_3I$ + **A** - LutHI	0	253.24	284.53	193.22	-10.09	-8.72	-3218.39773	-3221.55984	-3221.25980	0.00	0.00	0.00	0.00
**TS5**	48i	253.34	284.76	206.86	-22.95	-17.47	-3218.38988	-3221.54910	-3221.24428	4.92	6.74	9.74	7.92
**E** + B(C_6F_5)$_3$	0	256.37	286.82	196.95	-27.53	-17.69	-3218.39468	-3221.55668	-3221.26498	1.91	1.98	-3.25	-3.32
**E** + **A** - H_2 - Lut	0	263.82	293.36	223.59	-28.68	-24.15	-3218.43473	-3221.59873	-3221.28091	-23.22	-24.40	-13.25	-12.06

*Once formed, acetal $SimOCH_2OSim$ (**E**) can be slowly destroyed by $SiMe_3^+$ transfer from $SiMe_3I$ and subsequent H^- transfer from **A**.*													
**E** + $SiMe_3I$	0	231.72	249.29	185.33	-22.98	-12.12	-1715.82216	-1717.38844	-1717.10638	0.00	0.00	0.00	0.00
**TS6**	72i	232.36	250.40	198.56	-29.96	-18.92	-1715.79873	-1717.36337	-1717.07409	14.70	15.73	20.26	19.22
$\mathbf{F}^+$ + I^-	0	234.38	251.66	193.94	-137.35	-107.76	-1715.66580	-1717.23203	-1717.08868	98.12	98.15	11.10	11.07
**E** + A - LutHI	0	330.65	366.96	265.70	-12.29	-9.66	-3628.43716	-3631.95995	-3631.54589	0.00	0.00	0.00	0.00
$\mathbf{F}^+$ + A^-	0	333.38	368.39	268.79	-88.78	-78.68	-3628.31569	-3631.83639	-3631.52741	76.22	77.53	11.59	10.29
**TS7**	207i	330.66	367.50	279.25	-29.19	-22.40	-3628.41973	-3631.93806	-3631.52574	10.94	13.73	12.64	9.85
**F** + $B(C_6F_5)_3$ + $O(SiMe_3)_2$	0	331.86	367.31	253.66	-32.90	-20.26	-3628.45194	-3631.97465	-3631.59366	-9.27	-9.23	-29.98	-30.02
*Further $SiMe_3^+$ transfer to CH_3OSiMe_3 (**F**) followed by hydride transfer from **A** is still possible but over sizeable barrier of 24.4 kcal/mol (via **TS9**$^+$)*													
**F** + $SiMe_3I$	0	165.29	177.86	124.08	-20.03	-10.34	-1231.69790	-1232.82780	-1232.64052	0.00	0.00	0.00	0.00
**F**.$SiMe_3I$	0	166.16	179.34	137.05	-15.73	-9.47	-1231.70529	-1232.83528	-1232.62895	-4.64	-4.69	7.26	7.32
**TS8**	0	166.70	179.79	137.87	-32.11	-20.33	-1231.67620	-1232.80456	-1232.61423	13.61	14.59	16.50	15.52
$\mathbf{G}^+$ + I^-	0	168.14	180.40	132.66	-138.89	-110.15	-1231.53487	-1232.66565	-1232.62375	102.30	101.75	10.52	11.07
$\mathbf{G}^+$ + LutHI	0	267.29	284.16	220.64	-86.80	-68.28	-1559.21547	-1560.69326	-1560.44443	0.00	0.00	10.52	11.07
**TS9**$^+$	399i	262.63	281.88	226.69	-56.25	-46.36	-1559.24352	-1560.71272	-1560.42234	-17.60	-12.21	24.39	19.55
CH_3I + $O(SiMe_3)_2$ + $LutH^+$	0	263.93	281.38	205.72	-68.16	-56.67	-1559.24822	-1560.72884	-1560.48228	-20.55	-22.33	-13.23	-10.90
*..competitive hydride transfer from **A** to $\mathbf{G}^+$*													
**F** + **A** + $SiMe_3I$ - LutHI	0	264.22	295.53	204.45	-9.34	-7.88	-3144.31290	-3147.39931	-3147.08004	0.00	0.00	0.00	0.00
$\mathbf{G}^+$ + $\mathbf{A}^-$	0	267.13	297.13	207.51	-90.32	-81.07	-3144.18477	-3147.27001	-3147.06249	80.41	81.14	11.01	10.28
**TS10**	534i	264.50	295.87	217.62	-27.62	-21.35	-3144.28036	-3147.35634	-3147.04055	20.42	26.97	24.78	18.23
CH_4 + $O(SiMe_3)_2$ + $B(C_6F_5)_3$	0	264.27	295.37	196.01	-24.43	-15.90	-3144.34975	-3147.43743	-3147.14136	-23.12	-23.92	-38.48	-37.69
2,4,6-Collidine (Col) is 1.9 kcal/mol more basic than 2,6-lutidine (Lut)													
Col + $LutH^+$	0	206.44	217.06	167.56	-66.56	-56.27	-694.00872	-694.75213	-694.56875	0.00	0.00	0.00	0.00
Lut + $ColH^+$	0	206.41	217.05	167.51	-64.34	-54.31	-694.01457	-694.75814	-694.57171	-3.67	-3.77	-1.86	-1.75
Potential trapping of $B(C_6F_5)_3$ with Lewis bases in $CHCl_3$ solution													
Lut + $B(C_6F_5)_3$	0	184.29	207.96	132.02	-28.08	-19.16	-2536.76119	-2539.36459	-2539.17871	0.00	0.00	0.00	0.00
Lut.$B(C_6F_5)_3$	0	185.95	209.44	147.77	-22.85	-18.28	-2536.78641	-2539.39125	-2539.18187	-15.83	-16.73	-1.99	-1.09
..with 2.7 kcal/mol higher affinity of Col than Lut that may reduce the H_2-activation reactivity.													
Col + $B(C_6F_5)_3$	0	201.79	226.26	148.21	-29.46	-20.06	-2576.10613	-2578.75122	-2578.54097	0.00	0.00	0.00	0.00
Col.$B(C_6F_5)_3$	0	202.48	227.34	162.84	-23.94	-19.09	-2576.13381	-2578.78056	-2578.54848	-17.37	-18.42	-4.71	-3.66
..Halide anion F^-, Cl^- and Br^- is bound to $B(C_6F_5)_3$													

$B(C_6F_5)_3 + F^-$	0	94.53	114.73	52.22	-136.01	-102.49	-2309.51513	-2311.86513	-2311.93922	0.00	0.00	0.00	0.00
$FB(C_6F_5)_3^-$	0	94.66	114.46	59.44	-48.73	-41.85	-2309.68320	-2312.03662	-2312.00557	-105.47	-107.61	-41.64	-39.49
$B(C_6F_5)_3 + Cl^-$	0	94.53	114.73	51.67	-122.03	-90.10	-2669.93294	-2672.52066	-2672.57589	0.00	0.00	0.00	0.00
$ClB(C_6F_5)_3^-$	0	94.16	114.07	59.13	-48.17	-42.15	-2670.02438	-2672.61507	-2672.58500	-57.38	-59.24	-5.71	-3.85
$B(C_6F_5)_3 + Br^-$	0	94.53	114.73	50.94	-115.78	-85.67	-4783.81610	-4787.29162	-4787.34094	0.00	0.00	0.00	0.00
$BrB(C_6F_5)_3Br^-$	0	93.78	113.94	58.17	-48.60	-42.55	-4783.89261	-4787.37090	-4787.34299	-48.01	-49.74	-1.29	0.44
$B(C_6F_5)_3 + I^-$	0	94.53	114.73	50.53	-107.40	-80.35	-2507.30856	-2509.85749	-2509.89898	0.00	0.00	0.00	0.00
$IB(C_6F_5)_3^-$	0	93.75	113.97	58.05	-49.86	-43.55	-2507.37094	-2509.92203	-2509.89592	-39.14	-40.50	1.92	3.28
Halide anions are bound to $LutH^+$ cation													
$LutH^+ + Cl^-$	0	99.19	105.53	71.01	-159.13	-126.31	-787.83554	-788.52158	-788.60368	0.00	0.00	0.00	0.00
LutHCl	0	98.62	104.74	77.82	-40.18	-24.40	-788.01333	-788.69628	-788.60813	-111.57	-109.63	-2.80	-4.74
$LutH^+ + I^-$	0	99.19	105.53	69.88	-144.50	-116.55	-625.21115	-625.85841	-625.92677	0.00	0.00	0.00	0.00
LutHI	0	99.15	105.25	77.42	-40.58	-27.01	-625.36682	-626.01107	-625.92773	-97.69	-95.80	-0.60	-2.49
Salt LutHCl is also bound to $B(C_6F_5)_3$													
$LutHCl + B(C_6F_5)_3$	0	193.16	217.99	138.92	-54.90	-35.87	-2997.63566	-3000.57031	-3000.40007	0.00	0.00	0.00	0.00
$LutH^+ + ClB(C_6F_5)_3^-$	0	193.35	218.12	139.57	-99.99	-89.83	-2997.54931	-3000.49001	-3000.40472	54.19	50.39	-2.92	0.88
$LutHClB(C_6F_5)_3$	0	193.06	218.65	152.37	-30.55	-24.90	-2997.67479	-3000.61507	-3000.40892	-24.55	-28.09	-5.56	-2.02

Table S2. TPSS-D3/def2-TZVP + COSMO optimized Cartesian coordinates (in Å) in CHCl3 solution. Each structure is labeled by the specific name (See Table S1), followed by the number of atoms, the total energy (in hartrees), and the detailed atomic coordinates (in double-column text list).

A.CO_2 : complex of [LutH][HB(C_6F_5)$_3$] and CO_2
56
Energy = -2726.566212141

B	-0.3013620	0.3551583	0.6199155
C	-1.2843824	-0.1293539	1.8401267
C	-1.1985964	0.4568739	-0.7439572
C	1.0699925	-0.5357771	0.5176905
C	-1.3025272	-1.3824459	2.4505417
C	-2.2342683	0.7666785	2.3357193
C	-1.3371623	1.6410945	-1.4655006
C	-1.9703055	-0.6028090	-1.2239451
C	1.9513660	-0.5006079	1.6037615
C	1.5217012	-1.3132588	-0.5475855
C	-2.1717693	-1.7276745	3.4835186
F	-0.4485884	-2.3615238	2.0479186
C	-3.1253714	0.4685085	3.3618231
F	-2.3280485	2.0106799	1.7994056
C	-2.1554146	1.7782503	-2.5853727
F	-0.6442858	2.7588219	-1.1057141
C	-2.8048406	-0.5176537	-2.3327664
F	-1.9149741	-1.8051295	-0.5988641
C	3.1639648	-1.1780200	1.6561215
F	1.6340649	0.2405595	2.6981037
C	2.7368246	-1.9984669	-0.5520227
F	0.7869412	-1.4383214	-1.6859736
C	-3.0922426	-0.7941353	3.9450791
F	-2.1366434	-2.9603352	4.0370076
F	-4.0198013	1.3829589	3.7980975
C	-2.8974858	0.6879799	-3.0232879
F	-2.2333826	2.9527430	-3.2495222
F	-3.5198246	-1.5825706	-2.7534053
C	3.5664761	-1.9348980	0.5599309
F	3.9689726	-1.0894754	2.7374693
F	3.1250170	-2.7122760	-1.6308405
F	-3.9443908	-1.1088462	4.9406783
F	-3.6953928	0.7949570	-4.1028296
F	4.7527362	-2.5729573	0.5665637
H	0.0665428	1.4740800	0.8985507
C	4.5414017	1.2900935	-0.2118468
C	5.5153568	0.5138221	-0.8254909
C	5.3218023	0.0650423	-2.1300824
C	4.1560782	0.3914036	-2.8199368
C	3.1818133	1.1611404	-2.1999028
N	3.4206188	1.5711678	-0.9259123
H	6.0824071	-0.5409615	-2.6107651
H	6.4159278	0.2695981	-0.2753775
H	3.9899373	0.0520651	-3.8349800
C	4.6634520	1.8657727	1.1616016
H	5.4512271	1.3518402	1.7128974
H	4.9221016	2.9290628	1.0966312
H	3.7254635	1.7835558	1.7162831
C	1.8849510	1.5516523	-2.8252182
H	1.6441894	2.5971685	-2.6152972
H	1.9308096	1.4002098	-3.9039684
H	1.0756205	0.9319913	-2.4230163
H	2.7022453	2.1393436	-0.4686222
O	1.8421990	3.6065295	0.6064344
C	0.8946472	3.8669551	1.2484692
O	-0.0232070	4.1660186	1.8958050

A⁻ : anion HB(C_6F_5)$_3^-$
35
Energy = -2210.260699305

B	-0.0149056	-0.0067681	0.6432442
C	0.9195088	-1.2744247	0.1936152
C	-1.5783824	-0.1699714	0.1857561
C	0.6302561	1.4314031	0.1992463
C	1.8189725	-1.2977193	-0.8711092
C	0.8737712	-2.4598237	0.9293312
C	-2.5703849	0.5153108	0.8897350
C	-2.0612972	-0.9662340	-0.8513465
C	0.1923303	2.2520499	-0.8387498
C	1.7158048	1.9420963	0.9130643
C	2.6278045	-2.3885075	-1.1813904
F	1.9352663	-0.2255741	-1.7002665
C	1.6561544	-3.5781214	0.6571793
F	0.0173634	-2.5726208	1.9792921
C	-3.9306402	0.4233152	0.6121210
F	-2.2236029	1.3439093	1.9105532
C	-3.4124062	-1.0961840	-1.1659114
F	-1.2042687	-1.6607348	-1.6478165
C	0.7616683	3.4859094	-1.1459133
F	-0.8317559	1.8612193	-1.6446116
C	2.3214218	3.1654881	0.6438790
F	2.2512749	1.2225832	1.9350541
C	2.5471861	-3.5415585	-0.4097422
F	3.4828252	-2.3463246	-2.2302731
F	1.5647856	-4.6975258	1.4130473
C	-4.3581681	-0.3952909	-0.4275703
F	-4.8423849	1.1144085	1.3358993
F	-3.8170558	-1.8870563	-2.1874913
C	1.8362420	3.9497122	-0.3968660
F	0.2908060	4.2350951	-2.1705235
F	3.3709460	3.6044347	1.3776040

F	3.3194089	-4.6121291	-0.6944029
F	-5.6724777	-0.5033383	-0.7181542
F	2.4045317	5.1418106	-0.6784725
H	-0.0209564	-0.0090533	1.8535777

A : contact ion pair [LutH][HB$(C_6F_5)_3$]
53
Energy = -2537.867428391

B	-0.2444229	-0.2130921	0.2018416
C	-1.6302008	-0.7899428	-0.4432109
C	-0.4167026	0.0590939	1.7992857
C	0.4124813	1.0091098	-0.6632618
C	-2.5174328	-0.0778959	-1.2491349
C	-2.0019171	-2.1129425	-0.2009450
C	0.4959055	-0.4266452	2.7321794
C	-1.5057404	0.7324331	2.3528167
C	0.8382122	0.7556702	-1.9693190
C	0.6598543	2.3110099	-0.2273186
C	-3.6753719	-0.6281380	-1.7939484
F	-2.2865901	1.2279878	-1.5390328
C	-3.1465903	-2.7080990	-0.7216074
F	-1.2206146	-2.8984793	0.5899041
C	0.3577583	-0.2760352	4.1092981
F	1.6166778	-1.0918020	2.3173382
C	-1.6927497	0.9094455	3.7197618
F	-2.4434541	1.2764175	1.5377867
C	1.4668552	1.6858107	-2.7893047
F	0.6449011	-0.4828545	-2.5064865
C	1.2830323	3.2829368	-1.0102019
F	0.3012022	2.7062944	1.0188983
C	-3.9931326	-1.9559826	-1.5297354
F	-4.4959387	0.1118977	-2.5694563
F	-3.4450377	-3.9980776	-0.4551286
C	-0.7511037	0.3979876	4.6085053
F	1.2802309	-0.7732363	4.9610196
F	-2.7653535	1.5754185	4.1955541
C	1.6919805	2.9703509	-2.3022918
F	1.8586107	1.3622615	-4.0391094
F	1.4956371	4.5240803	-0.5283873
F	-5.1080901	-2.5061738	-2.0465912
F	-0.9096306	0.5586145	5.9354647
F	2.2932659	3.8954303	-3.0714679
H	0.5339591	-1.1486182	0.1171919
C	2.8776009	-2.7401894	-1.3145521
C	4.0553040	-3.1503518	-1.9243944
C	5.1920080	-2.3481207	-1.8387811
C	5.1500470	-1.1417365	-1.1436298
C	3.9662321	-0.7364786	-0.5397203
N	2.8900698	-1.5539431	-0.6531701
H	6.1149080	-2.6663679	-2.3127233
H	4.0718284	-4.0935539	-2.4572345
H	6.0263630	-0.5097937	-1.0621147
C	1.5991909	-3.5143877	-1.3295961
H	1.7331671	-4.4414446	-1.8874739
H	1.2813584	-3.7524066	-0.3095173
H	0.8012026	-2.9260640	-1.7927287
C	3.8018836	0.5493139	0.2037835
H	3.1362982	0.4278944	1.0607720
H	4.7730181	0.9106226	0.5448321
H	3.3669466	1.3079357	-0.4574611
H	1.9892720	-1.2567310	-0.2214255

BrB$(C_6F_5)_3^-$: bromide binding to B$(C_6F_5)_3$
35
Energy = -4783.767073362

B	-0.0032902	0.0028545	0.5783313
C	0.9355715	-1.2714125	0.1842105
C	-1.5752095	-0.1745727	0.1807270
C	0.6333985	1.4520676	0.1848990
C	2.0328990	-1.2191873	-0.6786008
C	0.6883184	-2.5482070	0.7070279
C	-2.5586184	0.6781717	0.7007065
C	-2.0773304	-1.1544903	-0.6791634
C	0.0390287	2.3820825	-0.6712854
C	1.8680593	1.8685740	0.7017175
C	2.8571636	-2.3087317	-0.9580885
F	2.3577632	-0.0760490	-1.3373118
C	1.4825110	-3.6603554	0.4566257
F	-0.4026166	-2.7696882	1.4760984
C	-3.9186025	0.5443402	0.4502955
F	-2.2061795	1.7350511	1.4684416
C	-3.4329163	-1.3259300	-0.9583582
F	-1.2492012	-2.0095777	-1.3342287
C	0.5770916	3.6376101	-0.9526637
F	-1.1203699	2.1011395	-1.3214539
C	2.4408074	3.1088025	0.4490385
F	2.6044230	1.0295232	1.4663913
C	2.5869643	-3.5406702	-0.3803124
F	3.9108122	-2.1779157	-1.7944360
F	1.1835122	-4.8616211	0.9985146
C	-4.3658166	-0.4752593	-0.3834295
F	-4.8103452	1.4048373	0.9890005
F	-3.8453911	-2.3067590	-1.7918758
C	1.7856143	4.0106364	-0.3828042
F	-0.0623787	4.4901116	-1.7840300
F	3.6365930	3.4417259	0.9831892
F	3.3692148	-4.6070626	-0.6384802
F	-5.6802489	-0.6222828	-0.6410818
F	2.3247211	5.2177484	-0.6435011
Br	-0.0062340	0.0043191	2.7520456

ClB$(C_6F_5)_3^-$: chloride binding to B$(C_6F_5)_3$

35
Energy = -2669.938586442

B	-0.0029853	0.0031902	0.6303640
C	0.9349870	-1.2710914	0.2046361
C	-1.5741921	-0.1737526	0.2005454
C	0.6340814	1.4514232	0.2044687
C	2.0095418	-1.2209233	-0.6847065
C	0.7044704	-2.5434322	0.7426884
C	-2.5622267	0.6638228	0.7332197
C	-2.0664002	-1.1355302	-0.6832883
C	0.0513990	2.3638388	-0.6764506
C	1.8576915	1.8791591	0.7350808
C	2.8256180	-2.3116018	-0.9832040
F	2.3189722	-0.0781714	-1.3527420
C	1.4909723	-3.6575144	0.4747220
F	-0.3656718	-2.7616853	1.5431996
C	-3.9200525	0.5360098	0.4660257
F	-2.2175378	1.7045969	1.5279745
C	-3.4188962	-1.3011534	-0.9804300
F	-1.2304723	-1.9778186	-1.3463255
C	0.5929729	3.6140837	-0.9741227
F	-1.0998750	2.0698317	-1.3365060
C	2.4339048	3.1153289	0.4683514
F	2.5833712	1.0542000	1.5267184
C	2.5715198	-3.5411857	-0.3929414
F	3.8573348	-2.1846294	-1.8477433
F	1.2073592	-4.8569816	1.0297832
C	-4.3579998	-0.4641957	-0.3952954
F	-4.8183318	1.3834042	1.0156986
F	-3.8233956	-2.2644398	-1.8386317
C	1.7918785	3.9993840	-0.3919591
F	-0.0344436	4.4514519	-1.8303367
F	3.6199857	3.4612406	1.0168223
F	3.3481363	-4.6083989	-0.6668955
F	-5.6703317	-0.6070918	-0.6681744
F	2.3335044	5.2028490	-0.6665061
Cl	-0.0051709	0.0056059	2.5921157

$IB(C_6F_5)_3^-$: iodide binding to $B(C_6F_5)_3$
35
Energy = -2507.297119770

B	-0.0029089	0.0024247	0.5144872
C	0.9355998	-1.2725348	0.1591478
C	-1.5752295	-0.1757844	0.1563383
C	0.6336776	1.4519861	0.1591005
C	2.0514405	-1.2228677	-0.6822782
C	0.6745611	-2.5509508	0.6739752
C	-2.5537971	0.6906633	0.6657606
C	-2.0878579	-1.1716977	-0.6813287
C	0.0277740	2.4022941	-0.6690135
C	1.8793834	1.8559356	0.6622743
C	2.8820200	-2.3126065	-0.9399643
F	2.3863639	-0.0834156	-1.3408788
C	1.4753654	-3.6623329	0.4452008
F	-0.4320137	-2.7700554	1.4197172
C	-3.9159714	0.5519454	0.4334361
F	-2.1925632	1.7599705	1.4103228
C	-3.4462102	-1.3463196	-0.9432230
F	-1.2672286	-2.0359302	-1.3322803
C	0.5624020	3.6635593	-0.9290426
F	-1.1369621	2.1344063	-1.3135821
C	2.4474196	3.1019204	0.4310907
F	2.6242899	1.0007565	1.3988221
C	2.5995148	-3.5435088	-0.3656024
F	3.9515480	-2.1836716	-1.7553861
F	1.1643732	-4.8619028	0.9825733
C	-4.3725735	-0.4836202	-0.3755125
F	-4.8008255	1.4226122	0.9654670
F	-3.8672912	-2.3405754	-1.7553933
C	1.7794489	4.0234445	-0.3688068
F	-0.0879584	4.5333563	-1.7328107
F	3.6502639	3.4222237	0.9554334
F	3.3873146	-4.6097056	-0.6037575
F	-5.6888623	-0.6326719	-0.6187176
F	2.3151064	5.2354108	-0.6102015
I	0.0001047	0.0070651	2.9807874

$B(C_6F_5)_3$: Lewis acidic borane
34
Energy = -2209.515840507

B	-0.0004361	0.0008220	0.0000543
C	-0.0001375	1.5634454	-0.0002793
C	1.3521791	-0.7825738	-0.0031074
C	-1.3528031	-0.7827121	0.0035198
C	-0.9390036	2.3140885	0.7245587
C	0.9392734	2.3133266	-0.7252105
C	2.4652097	-0.3612640	0.7399196
C	1.5356251	-1.9552537	-0.7512925
C	-1.5353823	-1.9564497	0.7503967
C	-2.4665976	-0.3609467	-0.7382299
C	-0.9469478	3.7025439	0.7462974
C	0.9481473	3.7017790	-0.7471988
C	3.6706593	-1.0503174	0.7571007
C	2.7338610	-2.6567057	-0.7805290
C	-2.7333768	-2.6583050	0.7796963
C	-3.6717743	-1.0504731	-0.7554297
C	0.0008195	4.3999087	-0.0005284
C	3.8062528	-2.2026396	-0.0150480
C	-3.8064447	-2.2038069	0.0153923
F	-1.8694110	1.6894036	1.4759324
F	1.8693611	1.6879369	-1.4764017
F	2.3824433	0.7414539	1.5134575

F 0.5329502 -2.4301995 -1.5195149
F -0.5321104 -2.4321007 1.5173460
F -2.3849326 0.7427168 -1.5104101
F -1.8509490 4.3779748 1.4728263
F 1.8525908 4.3764718 -1.4738463
F 4.7016982 -0.6229578 1.5024375
F 2.8715403 -3.7599952 -1.5322918
F -2.8701370 -3.7624867 1.5302886
F -4.7034421 -0.6226112 -1.4996020
F 0.0012505 5.7359161 -0.0006620
F 4.9631586 -2.8711082 -0.0210587
F -4.9630654 -2.8726708 0.0213904

Br^- : bromide
1
Energy = -2574.218173369
Br 0.0000000 0.0000000 0.0000000

B$^-$.$SiMe_3I$: loose complex of **B**$^-$ and $SiMe_3I$
52
Energy = -3106.012474801
O 0.3866301 0.1398805 -1.9317045
C -0.9208464 0.2689675 -1.9639326
H -1.3838904 0.4914587 -0.9842973
O -1.5953721 0.1540515 -2.9763636
Si -4.8723767 -0.4316370 -1.3943360
C -3.7724965 -1.7036347 -0.5705158
C -4.3659635 1.3336396 -1.0354424
C -5.0916848 -0.7543311 -3.2222323
H -2.7717121 -1.6709756 -1.0155350
H -3.6833819 -1.5111845 0.5031796
H -4.1739091 -2.7131771 -0.7051455
H -3.4423359 1.5585019 -1.5815367
H -5.1390631 2.0339608 -1.3680950
H -4.1948714 1.4932053 0.0341752
H -4.1189268 -0.6256386 -3.7127008
H -5.4479838 -1.7730434 -3.4067207
H -5.8034975 -0.0513608 -3.6671912
I -7.1391381 -0.7247056 -0.3289535
B 1.1065012 0.1807516 -0.5874572
C 1.1741897 1.6999939 0.0559441
C 0.3312361 -0.9208170 0.3643837
C 2.6704964 -0.2049829 -0.9361656
C 1.9059267 1.9014271 1.2289715
C 0.6528450 2.8689575 -0.4975485
C 0.2953056 -2.2576815 -0.0469998
C -0.4212190 -0.6571514 1.5085366
C 3.3223895 0.4851033 -1.9633372
C 3.4758290 -1.1218671 -0.2602694
C 2.1115098 3.1390105 1.8245553
C 0.8303049 4.1341727 0.0633532
C -0.3898698 -3.2637363 0.6236211
C -1.1237808 -1.6326537 2.2156764
C 4.6486349 0.2688111 -2.3260553
C 4.8078367 -1.3691177 -0.5868249
C 1.5656592 4.2736422 1.2326514
C -1.1055891 -2.9485516 1.7739845
C 5.4003404 -0.6723660 -1.6321656
F 2.4524591 0.8313701 1.8630613
F -0.0829005 2.8445193 -1.6393052
F 0.9647125 -2.6354079 -1.1637565
F -0.5413632 0.6047528 1.9966663
F 2.6709223 1.4515520 -2.6543383
F 2.9958269 -1.8383659 0.7875221
F 2.8236167 3.2544015 2.9666201
F 0.2959618 5.2280153 -0.5221901
F -0.3726065 -4.5368264 0.1748069
F -1.8442281 -1.3055090 3.3104105
F 5.2172428 0.9686324 -3.3324513
F 5.5309879 -2.2781926 0.1039112
F 1.7472033 5.4883239 1.7866222
F -1.7891571 -3.9012822 2.4349287
F 6.6872199 -0.8983952 -1.9622833

B$^-$: anion $OCHOB(C_6F_5)_3^-$
38
Energy = -2398.973786046
B -1.5961085 -0.7316217 -0.4069755
C -0.8440161 -1.4654977 0.8672074
C -1.8081015 0.8336505 0.0773639
C -2.9821204 -1.4794659 -0.8964134
C -1.4018497 -2.3931123 1.7473831
C 0.4674402 -1.0935401 1.1810124
C -1.2122516 1.9668658 -0.4749152
C -2.5777521 1.0908359 1.2149036
C -2.9261744 -2.8335491 -1.2448747
C -4.2248952 -0.8863393 -1.1161371
C -0.7174193 -2.9403228 2.8312361
F -2.6817284 -2.8196101 1.5995497
C 1.1900580 -1.6151746 2.2502795
F 1.1029613 -0.1496834 0.4444585
C -1.3659952 3.2518284 0.0473022
F -0.4331792 1.8865782 -1.5840026
C -2.7608036 2.3497315 1.7718845
F -3.2170728 0.0629939 1.8322619
C -4.0045293 -3.5595896 -1.7359245
F -1.7653126 -3.5187714 -1.0971267
C -5.3352781 -1.5724280 -1.6082987
F -4.4258858 0.4361369 -0.8785517
C 0.5923075 -2.5531468 3.0841185
F -1.3148351 -3.8420440 3.6419196
F 2.4568946 -1.2137709 2.4953843

C -2.1445500 3.4478426 1.1798646
F -0.7624780 4.3098691 -0.5378119
F -3.5295364 2.5230451 2.8695190
C -5.2271655 -2.9211950 -1.9187500
F -3.8843280 -4.8709973 -2.0348545
F -6.5104643 -0.9345212 -1.8019858
F 1.2698854 -3.0716307 4.1275118
F -2.3036984 4.6821859 1.6970214
F -6.2874303 -3.6007569 -2.3964141
O -0.6132664 -0.8620775 -1.5601882
C -0.9662848 -0.5324698 -2.7864814
O -0.2341811 -0.6585226 -3.7551114
H -1.9918175 -0.1292771 -2.8828956

B : contact ion pair [LutH][OCHOB(C_6F_5)$_3$]
56
Energy = -2726.585036755
B -1.6947811 -0.7904473 -0.3996197
C -0.9699978 -1.6297726 0.8235790
C -1.7933539 0.7697989 0.1018775
C -3.1189051 -1.4148540 -0.9272955
C -1.5168220 -2.6690490 1.5772610
C 0.3343015 -1.2852910 1.1898032
C -1.2608551 1.8890663 -0.5329544
C -2.4108988 1.0442504 1.3246980
C -3.1681373 -2.7345739 -1.3892712
C -4.3304488 -0.7292856 -1.0432731
C -0.8148117 -3.3575249 2.5661670
F -2.7980210 -3.0644196 1.3945883
C 1.0787517 -1.9469772 2.1607686
F 0.9543423 -0.2330746 0.5876766
C -1.3090022 3.1767006 -0.0012073
F -0.6555943 1.7867605 -1.7493322
C -2.4890045 2.3083444 1.8954216
F -3.0054632 0.0335282 2.0105197
C -4.3039091 -3.3481387 -1.9009519
F -2.0490916 -3.5048520 -1.3424564
C -5.4985570 -1.3023661 -1.5468469
F -4.4427165 0.5707140 -0.6763075
C 0.4983841 -3.0033827 2.8547521
F -1.3977714 -4.3616106 3.2495563
F 2.3464946 -1.5751670 2.4358563
C -1.9261241 3.3904637 1.2248524
F -0.7649992 4.2164444 -0.6668939
F -3.1059469 2.5008702 3.0785025
C -5.4887490 -2.6222324 -1.9780875
F -4.2741295 -4.6299700 -2.3154276
F -6.6343796 -0.5820869 -1.6297084
F 1.1910917 -3.6599260 3.8009515
F -1.9860917 4.6257002 1.7529460
F -6.6029890 -3.1881886 -2.4701441
O -0.6755489 -0.9492777 -1.5549137
C -0.9816108 -0.7362943 -2.8283834
O -0.1610319 -0.8971624 -3.7159217
H -2.0169320 -0.4130580 -3.0152986
C 2.3708965 -3.0442441 -1.2513076
C 3.6959205 -3.4119971 -1.0561727
C 4.7034409 -2.4618081 -1.2066267
C 4.3853616 -1.1503481 -1.5506856
C 3.0566043 -0.7914809 -1.7404277
N 2.1153519 -1.7550439 -1.5852230
H 5.7399578 -2.7442817 -1.0531964
H 3.9259819 -4.4368454 -0.7892837
H 5.1569191 -0.3985850 -1.6695649
C 1.2105662 -3.9773867 -1.1155023
H 1.3263079 -4.8188621 -1.8054276
H 1.1754163 -4.3862068 -0.1005517
H 0.2677254 -3.4724041 -1.3222451
C 2.6028724 0.5885495 -2.0925498
H 1.5201792 0.6380918 -2.1993201
H 2.9169218 1.2896556 -1.3128490
H 3.0677868 0.9043911 -3.0315177
H 1.1231170 -1.4740499 -1.6980455

CH_3I : product methyl iodide
5
Energy = -337.5394900037
C -0.0000070 -0.0000004 -1.8384228
H 0.0000065 1.0409015 -2.1511480
H -0.9014352 -0.5204604 -2.1511799
H 0.9014304 -0.5204502 -2.1511679
I 0.0000053 0.0000106 0.3257044

CH_4 : product methane
5
Energy = -40.54212270591
C -0.0000043 0.0000020 -0.0003787
H 0.0000019 1.0306080 -0.3647528
H -0.8925246 -0.5153126 -0.3647740
H 0.8925212 -0.5153084 -0.3647666
H 0.0000056 0.0000119 1.0927443

Cl^- : chloride anion
1
Energy = -460.3767818432
Cl 0.0000000 0.0000000 0.0000000

CO_2 : carbon dioxide
3
Energy = -188.6978139777
C -0.0000054 0.0000001 0.0000003
O 1.1686606 0.0000398 0.0000686

O -1.1686447 -0.0000365 -0.0000659

Col.B(C_6F_5)$_3$: adduct of 2,4,6-collidine
54
Energy = -2576.011449777

B	-0.1333268	-0.1062963	0.4295074
C	0.4826585	1.3960283	0.1230354
C	1.0523988	-1.0277223	-0.2794518
C	-1.6513944	-0.3394630	-0.1770572
C	-0.0770407	2.3649813	-0.7135639
C	1.7874890	1.6943180	0.5361160
C	2.0116755	-1.8290782	0.3434026
C	1.2663694	-0.8633590	-1.6544921
C	-2.7137535	0.4014086	0.3512761
C	-2.0498289	-1.2552335	-1.1558167
C	0.5815606	3.5367363	-1.0884465
F	-1.3159224	2.2179470	-1.2379081
C	2.4812673	2.8462769	0.1928236
F	2.4382219	0.8353581	1.3659326
C	3.0602454	-2.4548975	-0.3284999
F	1.9818911	-2.0444836	1.6841578
C	2.2943181	-1.4671873	-2.3678311
F	0.4414671	-0.0610157	-2.3720434
C	-4.0396822	0.2870872	-0.0467604
F	-2.4700032	1.3313507	1.3109145
C	-3.3648939	-1.4046097	-1.5944385
F	-1.1692320	-2.1173691	-1.7219494
C	1.8691953	3.7851944	-0.6326757
F	-0.0205902	4.4258203	-1.9008683
F	3.7259458	3.0692753	0.6576517
C	3.2046850	-2.2783400	-1.6980250
F	3.9415099	-3.2212080	0.3448608
F	2.4205647	-1.2710737	-3.6946322
C	-4.3726722	-0.6270570	-1.0399644
F	-4.9961151	1.0570906	0.5073539
F	-3.6687515	-2.3183691	-2.5364459
F	2.5140188	4.9095736	-0.9814898
F	4.2078846	-2.8742860	-2.3632708
F	-5.6443418	-0.7610578	-1.4481763
C	-0.1863906	0.4476854	3.0748779
C	-0.2583669	0.0240221	4.3976630
C	-0.4861451	-1.3029703	4.7474640
C	-0.6906190	-2.1783535	3.6883912
C	-0.6150107	-1.7596346	2.3662691
N	-0.3055420	-0.4568876	2.0409402
H	-0.1433880	0.7784778	5.1691231
H	-0.9146266	-3.2234991	3.8743934
C	-0.0207437	1.9345563	2.8950876
H	-0.4125230	2.4147040	3.7944165
H	1.0335685	2.2112776	2.8146039
H	-0.5512626	2.3271312	2.0342885
C	-0.9323593	-2.7978399	1.3284955
H	-0.3724492	-2.6949409	0.4032540
H	-0.7313149	-3.7838836	1.7514270
H	-1.9990954	-2.7503511	1.0827134
C	-0.5504788	-1.7458176	6.1798025
H	0.3241009	-1.3888658	6.7332143
H	-1.4372941	-1.3221160	6.6658536
H	-0.6012100	-2.8340570	6.2569596

[ColH][HB(C_6F_5)$_3$] : contact ion pair **cA**
56
Energy = -2577.212382218

B	-0.2553583	-0.2162724	0.1968714
C	-1.6474975	-0.7806619	-0.4465814
C	-0.4181444	0.0521295	1.7967537
C	0.4041393	1.0068680	-0.6646829
C	-2.5316347	-0.0604967	-1.2484415
C	-2.0270477	-2.1023160	-0.2084950
C	0.5043068	-0.4316427	2.7208406
C	-1.5045723	0.7206305	2.3614017
C	0.8363732	0.7538175	-1.9687882
C	0.6481062	2.3089823	-0.2273212
C	-3.6938597	-0.6014415	-1.7935200
F	-2.2934279	1.2452520	-1.5338597
C	-3.1766071	-2.6881434	-0.7289012
F	-1.2495886	-2.8958247	0.5780149
C	0.3781899	-0.2840913	4.0994078
F	1.6236705	-1.0923167	2.2950391
C	-1.6801219	0.8940530	3.7304245
F	-2.4509738	1.2647441	1.5564795
C	1.4707164	1.6840578	-2.7843653
F	0.6448921	-0.4836770	-2.5080903
C	1.2763695	3.2809830	-1.0061432
F	0.2795008	2.7047519	1.0158351
C	-4.0195308	-1.9281073	-1.5334358
F	-4.5110158	0.1466335	-2.5651275
F	-3.4837164	-3.9768745	-0.4654850
C	-0.7285969	0.3848428	4.6098263
F	1.3100100	-0.7799341	4.9419107
F	-2.7508921	1.5552334	4.2172801
C	1.6942727	2.9680932	-2.2952955
F	1.8696985	1.3610275	-4.0321028
F	1.4847702	4.5225867	-0.5233313
F	-5.1386052	-2.4698275	-2.0507282
F	-0.8757804	0.5426385	5.9385742
F	2.3029003	3.8924825	-3.0597329
H	0.5149250	-1.1564477	0.1044556
C	2.8826161	-2.7335398	-1.3125104
C	4.0547441	-3.1424253	-1.9260291
C	5.2123605	-2.3554848	-1.8574272
C	5.1509696	-1.1457195	-1.1548431

C	3.9754814	-0.7383843	-0.5436932
N	2.8930736	-1.5493823	-0.6466528
H	4.0602015	-4.0855947	-2.4614003
H	6.0244277	-0.5072659	-1.0800976
C	1.6038038	-3.5080683	-1.3310101
H	1.7493441	-4.4540935	-1.8531775
H	1.2585798	-3.7083464	-0.3120556
H	0.8192956	-2.9355327	-1.8357602
C	3.8239324	0.5480331	0.2024412
H	3.2499363	0.4029838	1.1207346
H	4.8049470	0.9560014	0.4479986
H	3.2884218	1.2784209	-0.4146182
H	1.9963993	-1.2513481	-0.2128981
C	6.4905113	-2.8116060	-2.4974778
H	7.0288305	-3.4723624	-1.8060780
H	6.2927974	-3.3799732	-3.4099566
H	7.1424984	-1.9660172	-2.7283975

ColH+ : *N*-protonated 2,4,6-collidine
21
Energy = -366.9096052395

N	0.0000261	-0.8542024	0.0055878
H	0.0000611	-1.8721910	0.0131510
C	1.2044519	-0.2251694	-0.0017745
C	1.2036099	1.1594377	-0.0145891
C	2.4384823	-1.0700841	0.0018177
C	-0.0000779	1.8773546	-0.0183620
H	2.1548465	1.6805174	-0.0248055
C	-1.2044420	-0.2252442	-0.0014636
H	3.0443167	-0.8516342	-0.8828701
H	3.0445568	-0.8437837	0.8844123
H	2.1959706	-2.1353561	0.0066732
C	-1.2037080	1.1593375	-0.0147779
C	-0.0001625	3.3767489	-0.0033631
C	-2.4383957	-1.0702549	0.0030755
H	-2.1549824	1.6803302	-0.0249430
H	-0.8923344	3.7776518	-0.4901012
H	-0.0012937	3.7290314	1.0362135
H	0.8929337	3.7777639	-0.4882965
H	-2.1958146	-2.1354214	0.0175358
H	-3.0487214	-0.8369432	0.8808349
H	-3.0399868	-0.8590265	-0.8863076

Col : Lewis base 2,4,6-collidine
20
Energy = -366.4654118029

N	0.0000534	-0.9367324	0.0075364
C	1.1582162	-0.2474601	0.0003620
C	1.1928562	1.1496777	-0.0119146
C	2.4302367	-1.0573894	0.0024117
C	-0.0000744	1.8770029	-0.0152018
H	2.1493715	1.6674158	-0.0229072
C	-1.1581944	-0.2475680	0.0008348
H	3.0392996	-0.8336760	-0.8811625
H	3.0401618	-0.8277778	0.8839240
H	2.1894134	-2.1222843	0.0062207
C	-1.1929502	1.1495567	-0.0119452
C	-0.0001564	3.3834798	-0.0000071
C	-2.4301578	-1.0576012	0.0037498
H	-2.1495270	1.6671939	-0.0227800
H	-0.8893883	3.7848219	-0.4949797
H	-0.0019345	3.7530862	1.0332785
H	0.8906739	3.7848881	-0.4920222
H	-2.1893182	-2.1224163	0.0171865
H	-3.0442546	-0.8207576	0.8803945
H	-3.0350605	-0.8412248	-0.8845498

[ColH][OCHOB(C_6F_5)$_3$] : contact ion pair **cB**
59
Energy = -2765.929766110

B	-1.6983446	-0.7962836	-0.4130804
C	-0.9631871	-1.6458879	0.7976613
C	-1.7792250	0.7665964	0.0841190
C	-3.1338876	-1.4105169	-0.9225449
C	-1.4921315	-2.7092705	1.5298935
C	0.3385403	-1.2926759	1.1649979
C	-1.2913438	1.8823891	-0.5917845
C	-2.3317870	1.0514301	1.3353091
C	-3.1902128	-2.7150588	-1.4255079
C	-4.3538026	-0.7320026	-0.9737878
C	-0.7760354	-3.4105579	2.4995782
F	-2.7693509	-3.1173549	1.3450409
C	1.0967294	-1.9662812	2.1170120
F	0.9416184	-0.2197583	0.5833080
C	-1.3179638	3.1755251	-0.0723114
F	-0.7515415	1.7698157	-1.8376620
C	-2.3856336	2.3215368	1.8957901
F	-2.8838980	0.0467941	2.0643332
C	-4.3366141	-3.3183064	-1.9251553
F	-2.0667015	-3.4811259	-1.4339941
C	-5.5330149	-1.2957685	-1.4622330
F	-4.4652711	0.5514766	-0.5529395
C	0.5336222	-3.0452062	2.7901981
F	-1.3424562	-4.4368166	3.1638250
F	2.3611255	-1.5845604	2.3938629
C	-1.8674927	3.3993696	1.1837516
F	-0.8204826	4.2112042	-0.7794110
F	-2.9403028	2.5240387	3.1078107
C	-5.5276232	-2.5986547	-1.9421301
F	-4.3114337	-4.5851548	-2.3838431
F	-6.6759861	-0.5824591	-1.4827950
F	1.2402144	-3.7146608	3.7170628

F	-1.9046719	4.6398608	1.7016548
F	-6.6524606	-3.1550408	-2.4206367
O	-0.6903562	-0.9584709	-1.5767554
C	-1.0051262	-0.7660983	-2.8513789
O	-0.1859595	-0.9242015	-3.7405674
H	-2.0467890	-0.4634924	-3.0376688
C	2.3629882	-3.0372143	-1.2510039
C	3.6837824	-3.3920180	-1.0274675
C	4.7083813	-2.4448237	-1.1442672
C	4.3633853	-1.1357253	-1.5006283
C	3.0404418	-0.7856912	-1.7209107
N	2.0979534	-1.7511433	-1.5883515
H	3.9104844	-4.4189876	-0.7614812
H	5.1288652	-0.3743541	-1.6070650
C	1.2103437	-3.9849457	-1.1431862
H	1.3483000	-4.8212550	-1.8351201
H	1.1580981	-4.3990349	-0.1312518
H	0.2666311	-3.4887419	-1.3668367
C	2.5888055	0.5927515	-2.0868264
H	1.5098766	0.6329816	-2.2328748
H	2.8671125	1.2951793	-1.2949269
H	3.0839988	0.9147095	-3.0079132
H	1.1071754	-1.4755133	-1.7173157
C	6.1364763	-2.8174984	-0.8696113
H	6.8252202	-2.1955504	-1.4466433
H	6.3576139	-2.6608306	0.1938707
H	6.3206638	-3.8707237	-1.0953446

cTS1 : TS for H_2-cleavage with Col.B$(C_6F_5)_3$

56

Energy = -2577.168759118

B	-0.3191856	0.7691643	-0.1023412
C	-0.2462922	1.1204138	1.4382533
C	0.0376129	1.9150591	-1.1323746
C	-1.0604009	-0.5435414	-0.5666138
C	-1.3033013	0.8726990	2.3202782
C	0.8770571	1.7299217	2.0085736
C	0.6621844	1.6686673	-2.3619137
C	-0.2506223	3.2615334	-0.8730401
C	-0.9904503	-1.7279370	0.1836505
C	-1.8077201	-0.6377801	-1.7488834
C	-1.2560076	1.1880919	3.6733870
F	-2.4522091	0.3241712	1.8650890
C	0.9740924	2.0391948	3.3601053
F	1.9478267	2.0259923	1.2379863
C	1.0021264	2.6696025	-3.2622578
F	0.9804009	0.4045794	-2.7110738
C	0.0559311	4.2910789	-1.7570001
F	-0.8885922	3.6193181	0.2638292
C	-1.5692757	-2.9253236	-0.2160505
F	-0.3241137	-1.7485429	1.3556569
C	-2.4137634	-1.8147585	-2.1769858
F	-1.9940323	0.4454634	-2.5321126
C	-0.1047314	1.7693317	4.1984764
F	-2.3041170	0.9393632	4.4771672
F	2.0875008	2.6005829	3.8622656
C	0.6927085	3.9933052	-2.9578617
F	1.6156624	2.3769013	-4.4215631
F	-0.2590750	5.5647711	-1.4657826
C	-2.2851503	-2.9691763	-1.4098513
F	-1.4505794	-4.0335931	0.5327610
F	-3.1209072	-1.8483916	-3.3191018
F	-0.0378990	2.0737903	5.5010075
F	1.0006078	4.9713462	-3.8186203
F	-2.8522623	-4.1126743	-1.8126732
H	1.4445110	0.1484437	-0.1904038
H	1.6495910	-0.6084160	-0.2034677
N	2.2309676	-2.4432710	-0.1899748
C	1.8562713	-3.3671424	-1.0957647
C	2.6200484	-2.8386732	1.0367575
C	1.8526444	-4.7287246	-0.7901686
C	1.4441922	-2.8640000	-2.4526607
C	2.6467804	-4.1872133	1.3958436
C	3.0035604	-1.7552597	2.0098414
C	2.2526302	-5.1631517	0.4768627
H	1.5328268	-5.4463040	-1.5412366
H	2.2808592	-2.3499691	-2.9377731
H	1.1167253	-3.6853090	-3.0943382
H	0.6321612	-2.1368561	-2.3621757
H	2.9645623	-4.4706490	2.3957733
H	2.1180544	-1.1770274	2.2977266
H	3.4479261	-2.1776750	2.9139730
H	3.7144407	-1.0611535	1.5514497
C	2.2787995	-6.6266487	0.8283717
H	1.4962770	-7.1746005	0.2955211
H	3.2427715	-7.0667750	0.5434412
H	2.1502169	-6.7777980	1.9037929

cTS2 : CO_2-reduction with [ColH][HB$(C_6F_5)_3$]

59

Energy = -2765.892615632

B	-0.9362211	-0.0666883	-0.1698801
C	-1.5318352	0.0339779	1.3206082
C	-1.9653793	-0.0649151	-1.3884921
C	0.3998092	-0.9608281	-0.2541852
C	-1.6382823	-1.1037249	2.1241525
C	-1.9800710	1.2142763	1.9132085
C	-1.5208170	0.1641067	-2.6956666
C	-3.3523409	-0.1890317	-1.2596043
C	1.4385622	-0.7501885	0.6596623
C	0.6200910	-2.0142271	-1.1480567
C	-2.1307775	-1.0852460	3.4246975

F	-1.2764483	-2.3162645	1.6320042
C	-2.4699679	1.2823108	3.2142678
F	-1.9534695	2.3761090	1.2186701
C	-2.3598341	0.2655088	-3.7955419
F	-0.1933761	0.3086953	-2.9334171
C	-4.2316066	-0.1021737	-2.3363229
F	-3.9194045	-0.4312829	-0.0543014
C	2.6186397	-1.4839499	0.6776640
F	1.3293439	0.2296000	1.5919837
C	1.7933484	-2.7657141	-1.1747446
F	-0.3333074	-2.3816513	-2.0314649
C	-2.5457809	0.1221585	3.9778043
F	-2.2154814	-2.2178882	4.1495145
F	-2.8765391	2.4549957	3.7381927
C	-3.7333086	0.1304531	-3.6132751
F	-1.8661642	0.4950476	-5.0274634
F	-5.5585466	-0.2461296	-2.1541939
C	2.8052944	-2.4948557	-0.2596765
F	3.5847500	-1.2196698	1.5796660
F	1.9539124	-3.7587450	-2.0686154
F	-3.0218793	0.1656554	5.2334918
F	-4.5674086	0.2196445	-4.6612034
F	3.9475897	-3.2002071	-0.2764791
H	-0.3679651	1.2445302	-0.4074927
C	4.1907606	2.0352191	1.0239592
C	5.4859020	1.5660166	1.1723778
C	6.0748553	0.7670111	0.1841751
C	5.3234275	0.4651465	-0.9583232
C	4.0321425	0.9471143	-1.1031586
N	3.5149902	1.7065291	-0.1057621
H	6.0315528	1.8170992	2.0754268
H	5.7405183	-0.1550380	-1.7443509
C	3.4773045	2.8749542	2.0350884
H	4.1415939	3.1039009	2.8688121
H	3.1302176	3.8093217	1.5826218
H	2.5970387	2.3457000	2.4137400
C	3.1691663	0.6882356	-2.2969737
H	3.0967774	1.5915728	-2.9131374
H	3.5954435	-0.1114741	-2.9037065
H	2.1557569	0.4102266	-1.9965070
H	2.5275549	2.0526867	-0.2040053
O	1.0510406	2.7534485	-0.3426736
C	-0.0301077	2.4358827	-0.8430119
O	-0.8703265	2.8743757	-1.5882341
C	7.4794995	0.2617783	0.3382280
H	8.1835636	1.0152430	-0.0370921
H	7.7197222	0.0795264	1.3888105
H	7.6356068	-0.6551498	-0.2350149

C : adduct $SiMe_3OCHOB(C_6F_5)_3$
51
Energy = -2808.248859059

B	0.1293319	-0.1292221	-0.1417427
C	1.0676302	-1.2159297	-0.9091872
C	0.0214233	1.3242659	-0.8741860
C	0.3898539	-0.0276338	1.4644429
C	1.0155524	-2.5701407	-0.5632186
C	2.0060578	-0.9094033	-1.8972459
C	-0.6633987	1.4805304	-2.0811560
C	0.5627601	2.5066044	-0.3652623
C	-0.5361932	0.6219288	2.2870496
C	1.5013595	-0.5343493	2.1400337
C	1.8010781	-3.5579826	-1.1423608
F	0.1527092	-2.9748642	0.4092277
C	2.8221869	-1.8643475	-2.5023057
F	2.1933886	0.3654424	-2.3106649
C	-0.8527137	2.6977867	-2.7238213
F	-1.1744988	0.3877446	-2.7192018
C	0.4083924	3.7492984	-0.9762920
F	1.3038196	2.4989217	0.7684981
C	-0.4127064	0.7295459	3.6671942
F	-1.6229848	1.2273939	1.7408253
C	1.6710052	-0.4452849	3.5206196
F	2.5135381	-1.1367267	1.4689556
C	2.7183094	-3.1984320	-2.1261201
F	1.6939726	-4.8436507	-0.7605639
F	3.7107919	-1.5065407	-3.4468721
C	-0.3119342	3.8496048	-2.1612329
F	-1.5353924	2.7706123	-3.8822318
F	0.9516460	4.8535629	-0.4317858
C	0.7036136	0.1848808	4.2938672
F	-1.3504409	1.3637567	4.3974837
F	2.7642936	-0.9637910	4.1113391
F	3.4928265	-4.1289491	-2.7029079
F	-0.4714749	5.0388853	-2.7622620
F	0.8488311	0.2770618	5.6251871
O	-1.3954322	-0.6576012	-0.1900116
C	-1.8602223	-1.5061261	-0.9896140
H	-1.2211489	-2.0585914	-1.6801889
O	-3.1135284	-1.7733083	-1.0418622
Si	-4.3935752	-0.9321584	-0.1381949
C	-4.2675489	0.8646261	-0.6139654
H	-5.1451058	1.4022097	-0.2345766
H	-3.3736292	1.3348901	-0.1959004
H	-4.2520150	0.9818896	-1.7034680
C	-5.9049909	-1.7704793	-0.8238709
H	-6.8058809	-1.3533530	-0.3583388
H	-5.9848449	-1.6221934	-1.9064109
H	-5.8881546	-2.8471539	-0.6219462
C	-4.1041428	-1.2935697	1.6648196
H	-3.9864911	-2.3694619	1.8353741
H	-3.2188757	-0.7768429	2.0438062

H -4.9701560 -0.9560658 2.2471566

D.$SiMe_3I$: loose complex of **D** and $SiMe_3I$
31
Energy = -1305.740022902
O -2.8198050 0.9721130 -0.0846617
Si -3.9521574 -0.3139387 0.0576269
C -3.0693199 2.2811014 -0.2196922
C -4.1666163 -0.6295508 1.8863718
C -5.5619664 0.1812105 -0.7587044
C -3.1284251 -1.7403851 -0.8171495
H -4.1381024 2.5351649 -0.3172339
O -2.1921263 3.1160189 -0.2393976
H -4.8541251 -1.4678799 2.0528907
H -3.2111182 -0.8793986 2.3613070
H -4.5800804 0.2497365 2.3942496
H -6.2468786 -0.6760353 -0.7549987
H -6.0632634 1.0020437 -0.2333826
H -5.4105481 0.4805770 -1.8022980
H -2.9546193 -1.5130744 -1.8747661
H -2.1642281 -1.9831353 -0.3573729
H -3.7613687 -2.6344102 -0.7624984
Si 1.1219378 0.3591886 -0.0203943
C 0.1935306 -0.3685723 1.4344766
C 0.3771121 -0.1325300 -1.6664548
C 1.3218129 2.2111686 0.1297822
H -0.8141171 0.0628252 1.4621688
H 0.1061014 -1.4569104 1.3497471
H 0.6964603 -0.1359719 2.3787630
H -0.6315537 0.2918909 -1.7413842
H 0.9773706 0.2512218 -2.4979737
H 0.3044326 -1.2203260 -1.7662437
H 1.7702105 2.4875863 1.0897991
H 1.9528178 2.6065380 -0.6732498
H 0.3303921 2.6765359 0.0556517
I 3.4267775 -0.6431706 0.0444365

D : neutral $SiMe_3OCHO$
17
Energy = -598.7069922209
O -2.1145777 0.7413448 0.5518487
C -1.9584320 -0.3800070 0.1080530
H -2.7844392 -1.0836358 -0.0730816
O -0.7827761 -0.9232553 -0.2228867
Si 0.7215577 -0.0759198 -0.0586141
C 0.9945525 0.3309059 1.7450187
H 1.9948696 0.7595828 1.8846362
H 0.2578841 1.0555591 2.1051377
H 0.9270994 -0.5700081 2.3659618
C 1.9454792 -1.3456372 -0.6758721
H 2.9660620 -0.9465961 -0.6300660
H 1.9126124 -2.2563799 -0.0669215
H 1.7387070 -1.6225682 -1.7159868
C 0.6868034 1.4413938 -1.1493420
H 0.4425513 1.1764288 -2.1846560
H -0.0499202 2.1688545 -0.7951189
H 1.6725206 1.9233864 -1.1510373

eC : adduct $SiEt_3OCHOB(C_6F_5)_3$
60
Energy = -2926.249880280
B 0.1862214 -0.0467077 -0.1363734
C 1.0426466 -1.1616884 -0.9620984
C 0.2954706 1.4615994 -0.7418750
C 0.3580040 -0.1206096 1.4832820
C 0.8037226 -2.5292578 -0.7921171
C 2.0892908 -0.8693157 -1.8406220
C -0.2760977 1.7912905 -1.9714939
C 0.9467664 2.5223229 -0.1095204
C -0.5090665 0.6084866 2.3037145
C 1.3020638 -0.8796223 2.1760531
C 1.5042176 -3.5372322 -1.4409050
F -0.1710902 -2.9339585 0.0703617
C 2.8267147 -1.8454492 -2.5097729
F 2.4683798 0.4081627 -2.0759366
C -0.2648921 3.0652799 -2.5258333
F -0.8740688 0.8212316 -2.7224899
C 0.9948566 3.8143984 -0.6280470
F 1.6016663 2.3350672 1.0614459
C -0.4942641 0.5607111 3.6918999
F -1.4184682 1.4490947 1.7466237
C 1.3611734 -0.9518884 3.5671927
F 2.2484554 -1.5922311 1.5176410
C 2.5314130 -3.1891607 -2.3135676
F 1.2110311 -4.8331748 -1.2298346
F 3.8237100 -1.4976908 -3.3431863
C 0.3774665 4.0927198 -1.8424140
F -0.8516362 3.3096755 -3.7129744
F 1.6363941 4.7955412 0.0333485
C 0.4507146 -0.2344605 4.3332208
F -1.3699107 1.2817793 4.4188160
F 2.2947025 -1.7086448 4.1746925
F 3.2280862 -4.1392822 -2.9536917
F 0.4122380 5.3324799 -2.3555518
F 0.4907370 -0.2951198 5.6737190
O -1.3885451 -0.4008729 -0.2814910
C -1.8892210 -1.1378028 -1.1655689
H -1.2730457 -1.5860281 -1.9463550
O -3.1383058 -1.4160193 -1.2137606
Si -4.4903494 -0.9312747 -0.1691055
C -5.0466713 0.7301784 -0.8250185
H -5.8699738 1.0495652 -0.1684903

H	-5.4953317	0.5818834	-1.8158087
C	-5.6974042	-2.3147869	-0.5205143
H	-6.5917244	-2.1224771	0.0897222
H	-5.2697645	-3.2501859	-0.1344476
C	-3.9341101	-0.8921937	1.6147731
H	-3.3301337	0.0051814	1.7802740
H	-4.8575593	-0.7448788	2.1957624
C	-3.1932273	-2.1414730	2.1293908
H	-2.9719568	-2.0456698	3.1974801
H	-3.7906474	-3.0489772	1.9945484
H	-2.2419141	-2.2841954	1.6089401
C	-6.0944348	-2.4872878	-1.9997729
H	-6.5782405	-1.5862770	-2.3909102
H	-5.2183809	-2.6933906	-2.6229932
H	-6.7950624	-3.3200620	-2.1204237
C	-3.9732606	1.8339347	-0.8947155
H	-3.4818265	1.9854036	0.0703455
H	-3.2003059	1.5828842	-1.6268255
H	-4.4185519	2.7861145	-1.2005739

eE : $CH_2(OSiEt_3)_2$
49
Energy = -1244.724041849

O	0.9490115	0.5110186	0.9221001
Si	2.1727289	-0.1199181	-0.0407287
C	-0.0333927	-0.2227083	1.6207702
C	2.8594603	1.3982095	-0.9125323
C	3.4592487	-0.9290224	1.0768716
C	1.5599519	-1.3600362	-1.3242206
H	-0.4971966	0.4728296	2.3314279
H	0.4175983	-1.0643116	2.1615613
O	-0.9997372	-0.7731719	0.7526590
H	3.6876202	1.0834215	-1.5634552
H	2.0776229	1.7792663	-1.5851722
H	3.7759017	-0.1933945	1.8290624
H	2.9813167	-1.7446684	1.6383691
H	2.3610799	-1.4574226	-2.0731766
H	0.7096238	-0.9130205	-1.8570112
Si	-2.1999492	0.0666889	-0.0696202
C	-1.5385791	1.4823639	-1.1278004
C	-2.9530224	-1.2491934	-1.1819398
C	-3.4463104	0.7236700	1.1849063
H	-2.3238497	1.7206547	-1.8615802
H	-0.6902623	1.1044726	-1.7146561
H	-3.7665723	-0.7927441	-1.7637655
H	-2.1887742	-1.5439373	-1.9154476
H	-3.7846551	-0.1143258	1.8098112
H	-2.9347300	1.4185015	1.8663632
C	-3.4703762	-2.4960328	-0.4400063
H	-2.6657722	-2.9691409	0.1323116
H	-4.2687769	-2.2360809	0.2645149
H	-3.8728739	-3.2406812	-1.1366744
C	4.6901528	-1.4720159	0.3232093
H	4.4039842	-2.2304349	-0.4143260
H	5.2106915	-0.6716495	-0.2150022
H	5.4108069	-1.9328578	1.0085254
C	-1.1267696	2.7642621	-0.3794498
H	-1.9683723	3.1825354	0.1842875
H	-0.3148569	2.5586176	0.3241812
H	-0.7797716	3.5369648	-1.0757403
C	-4.6613795	1.4270583	0.5468241
H	-5.2188427	0.7434020	-0.1034991
H	-5.3561183	1.8000669	1.3080935
H	-4.3516403	2.2817190	-0.0652856
C	1.1724186	-2.7558431	-0.8005631
H	2.0118359	-3.2338955	-0.2830562
H	0.3345347	-2.6879238	-0.1005477
H	0.8702941	-3.4181069	-1.6206161
C	3.3276731	2.5234186	0.0296178
H	4.1406931	2.1833282	0.6813131
H	3.6938670	3.3912005	-0.5314311
H	2.5071455	2.8587339	0.6722658

eF : CH_3OSiEt_3
27
Energy = -642.6215821375

O	1.2261842	0.2079176	-0.1364848
Si	1.7319466	-0.1775965	-1.6823042
C	-0.1321778	0.2169528	0.3098084
H	-0.1671485	0.7531858	1.2626920
H	-0.5027563	-0.8039678	0.4650257
H	-0.7867657	0.7275426	-0.4076167
C	1.7371119	-2.0435835	-1.9700930
C	0.3538374	-2.7192913	-2.0475982
H	2.3352857	-2.5081415	-1.1729570
H	2.2892225	-2.2329429	-2.9030212
H	0.4419085	-3.7928668	-2.2520760
H	-0.1982444	-2.6079653	-1.1080754
H	-0.2598277	-2.2810249	-2.8429412
C	3.4941014	0.4738446	-1.7683659
C	3.6148092	2.0001056	-1.5963778
H	3.9361428	0.1672889	-2.7272838
H	4.0810665	-0.0369443	-0.9914741
H	4.6613855	2.3263828	-1.6138097
H	3.0888020	2.5322487	-2.3974222
H	3.1774017	2.3207549	-0.6447998
C	0.6190853	0.6680686	-2.9515319
C	1.0775453	0.4549359	-4.4087554
H	0.5826242	1.7431546	-2.7260053
H	-0.4107889	0.3019483	-2.8362151
H	0.4116667	0.9601123	-5.1178976
H	2.0891196	0.8453597	-4.5687003

H 1.0921168 -0.6095260 -4.6695855

eG⁺ : aa
49
Energy = -1169.853933546
O -0.0030976 -0.3329396 -0.7571960
Si -1.6124461 0.3459598 -0.2727975
Si 1.6225169 -0.4320552 0.0309257
C -0.0596334 -1.0026758 -2.0904991
C -1.7520988 1.9532194 -1.2135923
C -1.6379044 0.5520352 1.5820045
C -2.8659352 -0.9250244 -0.8297449
C 2.8526573 0.0615521 -1.2900143
C 1.7874847 -2.2181630 0.5464052
C 1.6344542 0.7761697 1.4540449
H 0.9616783 -1.1194920 -2.4437752
H -0.6185400 -0.3541478 -2.7633444
H -0.5439150 -1.9686488 -1.9660581
H -2.8062126 2.2576163 -1.1393552
H -1.5846980 1.7616871 -2.2821922
C -0.8429355 3.0972695 -0.7258074
H -0.9686595 1.3601263 1.8897723
H -1.2891209 -0.3588260 2.0824820
C -3.0790730 0.8829232 2.0433557
H -3.8387851 -0.4572327 -0.6164624
H -2.8436096 -1.0421681 -1.9197486
C -2.7932714 -2.2966526 -0.1311855
H 3.8218907 0.0740438 -0.7694836
H 2.9557308 -0.7244910 -2.0481140
C 2.6053835 1.4298154 -1.9533205
H 1.6419812 -2.8554001 -0.3367073
H 2.8388276 -2.3622716 0.8347555
C 0.8625031 -2.6680431 1.6934451
H 1.2478035 1.7534657 1.1423994
H 0.9861549 0.4196893 2.2599499
C 3.0765983 0.9367597 1.9951997
H -1.0148318 4.0049123 -1.3119402
H -1.0339002 3.3389957 0.3245654
H 0.2154162 2.8380801 -0.8246546
H -3.7699930 0.0620827 1.8296947
H -3.0952441 1.0622813 3.1221530
H -3.4626111 1.7834340 1.5517338
H -2.8634975 -2.1957036 0.9565680
H -3.6131959 -2.9421098 -0.4592601
H -1.8561291 -2.8180449 -0.3541895
H 3.4027220 1.6660856 -2.6639226
H 1.6578268 1.4465957 -2.5025010
H 2.5746866 2.2328011 -1.2098634
H 1.0306170 -3.7212386 1.9371791
H 1.0400162 -2.0835979 2.6015969
H -0.1930535 -2.5573945 1.4250062
H 3.7368652 1.3927432 1.2517761
H 3.0752390 1.5785601 2.8808458
H 3.5091122 -0.0269832 2.2852141

$O(SiEt_3)_2$: by-product
45
Energy = -1130.142339587
O 0.0003007 -0.0031113 -0.0876056
Si 1.6318633 0.1847553 -0.0839185
C 2.2494237 -0.0064855 1.6870867
C 2.3838663 -1.1410674 -1.1933431
C 2.0344603 1.9046443 -0.7428299
H 3.3489722 0.0236492 1.6771642
H 1.9310165 0.8715871 2.2668241
H 3.4660942 -0.9593789 -1.2691506
H 2.2776339 -2.1156907 -0.6961599
H 1.8147350 1.9279381 -1.8197352
H 3.1190138 2.0656365 -0.6546832
Si -1.6318935 -0.1853825 -0.0841669
C -2.2492327 0.0075036 1.6867300
C -2.3808172 1.1421509 -1.1936632
C -2.0383215 -1.9043135 -0.7432090
H -1.9331940 -0.8714146 2.2664735
H -3.3488537 -0.0197270 1.6766011
H -3.4633900 0.9624259 -1.2694025
H -2.2729019 2.1166224 -0.6965626
H -3.1231739 -2.0633226 -0.6552687
H -1.8183742 -1.9279054 -1.8200714
C -1.7590348 1.2104424 -2.6017542
H -0.6841461 1.4112091 -2.5444365
H -1.8867800 0.2647353 -3.1406064
H -2.2159262 2.0015414 -3.2077274
C 1.7620612 -1.2105915 -2.6013748
H 0.6874844 -1.4130162 -2.5439204
H 1.8882966 -0.2647802 -3.1404075
H 2.2201416 -2.0010727 -3.2072594
C -1.7610384 1.2896897 2.3879324
H -2.1335864 1.3548586 3.4168741
H -0.6671621 1.3216794 2.4252751
H -2.0964136 2.1876383 1.8565979
C -1.2800911 -3.0450451 -0.0376833
H -1.5121761 -3.0762985 1.0329025
H -1.5360127 -4.0233161 -0.4610919
H -0.1974383 -2.9115737 -0.1334389
C 1.2741107 3.0439555 -0.0373277
H 1.5056810 3.0752940 1.0333635
H 1.5286434 4.0227473 -0.4603619
H 0.1917148 2.9088912 -0.1335580
C 1.7644525 -1.2899813 2.3881401
H 2.1364909 -1.3539262 3.4173429
H 0.6706486 -1.3252010 2.4246866

H 2.1028527 -2.1869938 1.8571463

$SiEt_3I$: electrophilic silane
23
Energy = -825.0340619176

I	-0.0011121	-0.0014854	1.3179177
Si	-0.0005620	-0.0000963	-1.1938974
C	0.0513489	1.8007071	-1.7352649
C	1.5326727	-0.9452630	-1.7366546
C	-1.5856133	-0.8548280	-1.7375907
H	-0.0453412	1.7964975	-2.8321762
H	-0.8459331	2.3025570	-1.3513178
H	1.5734424	-0.8642421	-2.8340867
H	2.4164756	-0.4162484	-1.3579605
H	-1.5706913	-1.8844143	-1.3579762
H	-1.5343979	-0.9318080	-2.8348521
C	1.3121593	2.5780746	-1.3186519
H	1.2642271	3.6176131	-1.6611027
H	1.4229080	2.5864741	-0.2288617
H	2.2161747	2.1279669	-1.7422685
C	1.5790575	-2.4240333	-1.3138347
H	2.5015410	-2.9032133	-1.6597389
H	1.5375033	-2.5192700	-0.2233624
H	0.7351268	-2.9844702	-1.7299288
C	-2.8894110	-0.1542425	-1.3172093
H	-3.7656011	-0.7126388	-1.6647021
H	-2.9529755	-0.0704728	-0.2269186
H	-2.9509990	0.8568362	-1.7336468

E : $CH_2(OSiMe_3)_2$
31
Energy = -1008.723462219

O	0.7148240	0.3692961	0.8462215
Si	2.1515984	0.1041140	0.0120774
C	-0.0727421	-0.6872228	1.3695713
C	2.6152393	1.7865122	-0.6634808
C	3.4506440	-0.5261095	1.2148132
C	1.9017709	-1.1508950	-1.3606874
H	-0.5662799	-0.2977565	2.2678047
H	0.5465329	-1.5548864	1.6298234
O	-1.0344572	-1.1406452	0.4396825
H	3.5719149	1.7402941	-1.1980691
H	1.8564960	2.1540666	-1.3639693
H	2.7157153	2.5208644	0.1441564
H	4.4106337	-0.6764858	0.7051694
H	3.6099028	0.1861417	2.0329716
H	3.1589777	-1.4872491	1.6559030
H	1.5210299	-2.0997676	-0.9650519
H	1.1848156	-0.7890170	-2.1050622
H	2.8506987	-1.3565220	-1.8720286
Si	-2.2002351	-0.1283077	-0.2295844
C	-3.5842892	-1.2868014	-0.7327413
C	-2.7720854	1.1168041	1.0554394
C	-1.5315856	0.7692823	-1.7364287
H	-3.2207678	-2.0481983	-1.4332252
H	-4.0077045	-1.8019760	0.1372733
H	-4.3933319	-0.7358649	-1.2279897
H	-1.9528708	1.7801557	1.3568137
H	-3.5734084	1.7439832	0.6453604
H	-3.1613054	0.6227621	1.9536701
H	-2.3210366	1.3680473	-2.2085391
H	-0.7135364	1.4423780	-1.4594280
H	-1.1551576	0.0630034	-2.4854893

E$^+$: cation $HC(OSiMe_3)_2^+$
30
Energy = -1007.936208311

O	0.5722660	-0.1164383	0.0624155
Si	2.3316073	0.2143804	-0.0270354
C	-0.0675469	-0.9082342	0.8247857
C	2.4023032	2.0283404	0.3565900
C	3.1327268	-0.8804180	1.2434122
C	2.7126378	-0.2300603	-1.7872298
H	0.4529665	-1.5273215	1.5595730
O	-1.3276829	-1.0195117	0.7713131
H	3.4404546	2.3760687	0.2941635
H	1.8104917	2.6109370	-0.3578058
H	2.0384085	2.2379337	1.3683472
H	4.2159469	-0.7084950	1.2130223
H	2.7967347	-0.6549939	2.2617707
H	2.9669201	-1.9443680	1.0396959
H	2.5603798	-1.2986742	-1.9733163
H	2.0900587	0.3408394	-2.4847240
H	3.7615802	0.0037416	-2.0055680
Si	-2.4726025	-0.1386420	-0.3088094
C	-4.0900326	-0.7984910	0.3123679
C	-2.2027188	1.6688608	0.0365718
C	-2.0256912	-0.6841291	-2.0291277
H	-4.1435357	-1.8871324	0.2035153
H	-4.2457431	-0.5449973	1.3665283
H	-4.9126960	-0.3605707	-0.2655622
H	-1.2365589	2.0235140	-0.3344963
H	-2.9876224	2.2448668	-0.4692901
H	-2.2729380	1.8839713	1.1085243
H	-2.7617689	-0.2778625	-2.7338002
H	-1.0380367	-0.3225302	-2.3323177
H	-2.0468410	-1.7756974	-2.1183607

F.$SiMe_3I$: loose complex of **F** and $SiMe_3I$
32
Energy = -1231.659538256

O 2.7421157 0.5379029 0.7096414

C	3.1218373	0.2776518	2.0639947
H	3.1728204	-0.8012857	2.2583172
H	4.0935688	0.7282527	2.3035503
H	2.3633155	0.7199199	2.7158446
Si	3.7662643	0.2388713	-0.5814787
C	2.7734143	0.7037260	-2.0982144
C	4.2532917	-1.5764352	-0.6169635
C	5.3098298	1.3005593	-0.4327974
H	3.3742887	0.5673828	-3.0056807
H	1.8764366	0.0821871	-2.1932721
H	2.4570825	1.7521964	-2.0578915
H	4.7979776	-1.8661695	0.2898868
H	3.3756978	-2.2273881	-0.7065121
H	4.9088924	-1.7797880	-1.4730133
H	5.8889715	1.0539526	0.4652703
H	5.9662508	1.1468079	-1.2985756
H	5.0544428	2.3659703	-0.3893949
C	-0.9784461	-0.3133685	2.3688317
C	-0.5275555	1.3952999	-0.1842474
C	-0.0276267	-1.6807904	-0.2447325
H	0.0518604	-0.1645835	2.7159100
H	-1.6013354	0.4802909	2.7939336
H	-1.3333248	-1.2752054	2.7529398
H	-0.6151022	1.4216843	-1.2752080
H	-1.1542477	2.1901723	0.2331368
H	0.5187706	1.5846875	0.0854714
H	-0.3757371	-2.6496884	0.1273275
H	-0.0948874	-1.6853030	-1.3373618
H	1.0241144	-1.5529755	0.0381981
Si	-1.0174353	-0.2755527	0.4961885
I	-3.4103913	-0.6595008	-0.1682398

F : product CH_3OSiMe_3
18
Energy = -524.6226891580

O	1.3030419	-1.1342065	-1.1048727
Si	2.5226214	-2.0448298	-0.4105516
C	-0.0365482	-1.1295752	-0.5979839
C	2.0811956	-3.8716795	-0.4692175
C	4.0343898	-1.6809663	-1.4530500
C	2.7881499	-1.5194342	1.3753584
H	-0.0669551	-0.7737600	0.4398867
H	-0.4813758	-2.1319249	-0.6442836
H	-0.6262838	-0.4520183	-1.2213016
H	2.8965871	-4.4797659	-0.0575908
H	1.1807776	-4.0902071	0.1177209
H	1.9020801	-4.2027559	-1.4991706
H	4.9047940	-2.2354017	-1.0812167
H	3.8720721	-1.9706191	-2.4978210
H	4.2809701	-0.6130230	-1.4308608
H	3.0289863	-0.4517296	1.4422268
H	1.8987050	-1.7045837	1.9899328
H	3.6183695	-2.0801014	1.8230649

F$^+$: cation $SiMe_3OCH_2O(SiMe_3)_2^+$
44
Energy = -1417.953343650

O	1.6243761	0.4653321	-1.0879704
C	3.0690538	0.8533055	-0.6646248
H	3.0037339	0.9564838	0.4183453
O	3.9248655	-0.0898533	-1.1087020
Si	4.6482293	-1.3653393	-0.2112040
C	5.2083137	-0.6596447	1.4267495
H	4.3756772	-0.3368258	2.0622369
H	5.7572832	-1.4278519	1.9849585
H	5.8808971	0.1934894	1.2828837
C	3.4103713	-2.7490827	0.0199261
H	3.8939978	-3.5794617	0.5493027
H	2.5438668	-2.4479946	0.6178786
H	3.0482690	-3.1345597	-0.9386921
C	6.0604636	-1.8782905	-1.3094614
H	5.6982838	-2.2036756	-2.2914840
H	6.7639422	-1.0522209	-1.4607460
H	6.6098845	-2.7146585	-0.8617003
H	3.2279995	1.8077098	-1.1638628
Si	1.4198264	0.2572442	-2.8687243
C	1.6946708	-1.5415753	-3.2451574
C	2.6740129	1.3805069	-3.6599579
C	-0.3066151	0.8329393	-3.2627976
H	1.4645899	-1.7164492	-4.3035855
H	2.7362506	-1.8282823	-3.0757984
H	1.0482405	-2.1967682	-2.6538207
H	2.5287617	2.4293177	-3.3790602
H	3.7056223	1.0878671	-3.4457520
H	2.5272983	1.3122303	-4.7457052
H	-0.4436601	1.8937939	-3.0318534
H	-0.4319599	0.7135679	-4.3473353
H	-1.1031571	0.2584772	-2.7832881
C	1.1836751	0.1500054	1.8008618
C	-0.7271000	1.7877582	0.0552989
C	-0.5239780	-1.3271327	-0.2787118
H	1.9253788	-0.6516843	1.8640362
H	1.6423573	1.0899544	2.1243485
H	0.3956347	-0.0871625	2.5281151
H	-1.3425434	1.8326670	-0.8463282
H	-1.4013159	1.7898711	0.9209491
H	-0.1179206	2.6971828	0.1085676
H	-1.3165401	-1.4846546	0.4640177
H	-0.9982264	-1.3286503	-1.2636281
H	0.1593938	-2.1803556	-0.2187353
Si	0.3380434	0.2668020	0.1439853

G^{+} : cation $CH_3O(SiMe_3)_2^{+}$
31
Energy = -933.8513426050
O 1.1868794 -0.3095876 -1.0251552
C 2.4874511 -0.1478842 -0.3123320
Si 1.2789590 0.1365535 -2.7693034
Si -0.2866529 -0.4493726 0.0133887
H 2.6380373 -1.0321524 0.3011842
H 2.4439318 0.7622236 0.2841092
H 3.2651024 -0.0759837 -1.0702966
C -0.4089387 -0.1384971 -3.4918442
C 2.5343616 -1.0315007 -3.4887480
C 1.7860774 1.9252943 -2.7644902
C 0.3484160 -0.9342881 1.6926788
C -1.3225413 -1.8009600 -0.7304900
C -1.0415364 1.2499511 -0.0026653
H -0.3545185 0.2254118 -4.5268907
H -0.6815137 -1.1965023 -3.5315097
H -1.2065393 0.4222660 -2.9974193
H 3.5591133 -0.8397721 -3.1566735
H 2.2804641 -2.0727301 -3.2616613
H 2.5186931 -0.9161343 -4.5799043
H 2.7606687 2.0877388 -2.2927362
H 1.8604443 2.2760489 -3.8010805
H 1.0443395 2.5494659 -2.2543077
H 0.8701862 -1.8969981 1.6836103
H 0.9954686 -0.1816771 2.1527439
H -0.5317942 -1.0473395 2.3391348
H -2.0200352 -2.1433967 0.0445997
H -1.9144532 -1.4805946 -1.5900370
H -0.7088123 -2.6605618 -1.0212886
H -1.3765495 1.5598636 -0.9972881
H -1.9186128 1.2547160 0.6562747
H -0.3384712 1.9995988 0.3772355

H_2 : dihydrogen
2
Energy = -1.180052220925
H 0.0279549 0.0000000 0.0000000
H 0.7720451 0.0000000 0.0000000

I^{-} : anion iodide
1
Energy = -297.7563068331
I 0.0000000 0.0000000 0.0000000

Lut.B$(C_6F_5)_3$: adduct of 2,6-lutidine
51
Energy = -2536.666799414
B -0.1545118 -0.1063172 0.4286344
C 0.4711989 1.3926875 0.1298294
C 1.0378626 -1.0335033 -0.2605125
C -1.6654215 -0.3347050 -0.1948901
C -0.0727155 2.3615665 -0.7172839
C 1.7699393 1.6879764 0.5636998
C 1.9862915 -1.8363156 0.3768611
C 1.2712178 -0.8720463 -1.6328568
C -2.7314197 0.4115314 0.3182602
C -2.0541257 -1.2489230 -1.1792486
C 0.5952874 3.5306457 -1.0837472
F -1.3033727 2.2165017 -1.2609269
C 2.4722786 2.8374967 0.2297704
F 2.4039022 0.8285723 1.4058896
C 3.0426628 -2.4658517 -0.2791459
F 1.9374724 -2.0485618 1.7176889
C 2.3076687 -1.4795749 -2.3306343
F 0.4586263 -0.0688120 -2.3630682
C -4.0521870 0.3033480 -0.0982699
F -2.4964426 1.3402576 1.2812974
C -3.3636258 -1.3921913 -1.6360762
F -1.1696067 -2.1151376 -1.7325103
C 1.8760958 3.7764090 -0.6074540
F 0.0090799 4.4194760 -1.9076805
F 3.7095969 3.0580689 0.7144155
C 3.2065193 -2.2920567 -1.6469001
F 3.9123040 -3.2328535 0.4079225
F 2.4528849 -1.2857985 -3.6556648
C -4.3754177 -0.6096227 -1.0958309
F -5.0124251 1.0778885 0.4421861
F -3.6584932 -2.3042970 -2.5822363
F 2.5293726 4.8983073 -0.9476690
F 4.2171488 -2.8915577 -2.2971209
F -5.6416630 -0.7379774 -1.5214983
C -0.2349810 0.4547495 3.0709357
C -0.3143997 0.0338003 4.3985180
C -0.5485725 -1.2899148 4.7247226
C -0.7520690 -2.1792684 3.6837091
C -0.6642107 -1.7593111 2.3610931
N -0.3483759 -0.4547867 2.0427888
H -0.5993954 -1.6127336 5.7594743
H -0.2029328 0.7848397 5.1720583
H -0.9824299 -3.2213520 3.8725366
C -0.0716008 1.9409313 2.8886792
H -0.4878195 2.4219279 3.7766837
H 0.9840133 2.2206175 2.8356372
H -0.5806530 2.3293547 2.0136030
C -0.9761223 -2.7932228 1.3182780
H -0.4028817 -2.6950993 0.4006416
H -0.7898048 -3.7810165 1.7438200
H -2.0389963 -2.7360600 1.0580751

[LutH][ClB$(C_6F_5)_3$] : contact ion pair

53
Energy = -2997.549193120

B	-0.2418707	-0.7212248	0.2294848
C	-1.7803330	-1.0543314	-0.2100579
C	-0.3159526	-0.0843539	1.7360448
C	0.6442624	0.1417149	-0.8391634
C	-2.5101916	-0.3906391	-1.1991142
C	-2.5145183	-2.0117816	0.4998624
C	0.1817581	-0.5827007	2.9411166
C	-1.0434246	1.1044907	1.8703860
C	0.7153095	-0.2561057	-2.1813948
C	1.4869889	1.2084790	-0.5197025
C	-3.8396326	-0.6771184	-1.5013642
F	-1.9552369	0.6126078	-1.9232345
C	-3.8405171	-2.3335749	0.2285493
F	-1.9437610	-2.6745990	1.5346223
C	-0.0280849	0.0372448	4.1742085
F	0.9245752	-1.7151867	2.9965783
C	-1.2791398	1.7545754	3.0741827
F	-1.5426523	1.7028191	0.7582055
C	1.5477056	0.3304188	-3.1266164
F	-0.0373602	-1.2916610	-2.6212064
C	2.3465311	1.8208305	-1.4334627
F	1.5505101	1.7071079	0.7400280
C	-4.5109189	-1.6624557	-0.7880531
F	-4.4848406	-0.0001539	-2.4736471
F	-4.4839829	-3.2773347	0.9456312
C	-0.7653316	1.2109585	4.2472330
F	0.4859950	-0.4976898	5.3012507
F	-1.9846072	2.9026094	3.1175365
C	2.3826214	1.3780123	-2.7478937
F	1.5784836	-0.1185259	-4.3980474
F	3.1601912	2.8223992	-1.0429262
F	-5.7932225	-1.9544260	-1.0684404
F	-0.9746695	1.8157902	5.4297590
F	3.2194178	1.9390916	-3.6372918
C	3.8515353	-1.8703231	-2.1290425
C	4.9360358	-1.2373622	-2.7219599
C	5.6881174	-0.3237209	-1.9852511
C	5.3621852	-0.0518964	-0.6587386
C	4.2747090	-0.6904486	-0.0746706
N	3.5689843	-1.5609892	-0.8379966
H	6.5310891	0.1787389	-2.4480599
H	5.1761223	-1.4589101	-3.7550043
H	5.9364125	0.6556198	-0.0724851
C	2.9908854	-2.8881393	-2.8052811
H	2.9541151	-2.6993555	-3.8793553
H	3.4161680	-3.8868067	-2.6493500
H	1.9775790	-2.8870155	-2.3967536
C	3.8470370	-0.5051846	1.3454091
H	2.8162277	-0.8306651	1.4953715
H	4.4921796	-1.1032778	1.9995636
H	3.9476497	0.5413209	1.6390725
H	2.7047017	-1.9698033	-0.4235794
Cl	0.7187915	-2.4568612	0.2058384

LutHCl : H-bonded chloride
19
Energy = -788.0006558902

C	0.6271125	-1.1977183	-0.0000718
C	2.0179947	-1.2075884	-0.0004740
C	2.7129600	-0.0003718	-0.0008050
C	2.0183861	1.2070710	-0.0008432
C	0.6274996	1.1976512	-0.0003021
N	-0.0031334	0.0000667	-0.0001859
H	3.7984189	-0.0005478	-0.0011072
H	2.5421541	-2.1565800	-0.0005596
H	2.5428621	2.1558901	-0.0016949
C	-0.2205846	-2.4330364	0.0006477
H	0.0039949	-3.0384998	-0.8834315
H	0.0061952	-3.0389055	0.8838773
H	-1.2825481	-2.1770244	0.0020609
C	-0.2198086	2.4332377	0.0006840
H	-1.2818546	2.1775661	0.0022423
H	0.0074012	3.0391154	0.8838004
H	0.0047252	3.0385435	-0.8835141
H	-1.0853972	0.0002403	-0.0003602
Cl	-2.9878277	0.0005983	0.0000728

LutHI : H-bonded iodide
19
Energy = -625.3682660026

C	0.6495439	-1.2019220	0.0000705
C	2.0396296	-1.2082219	0.0009810
C	2.7336131	-0.0003750	0.0017225
C	2.0400226	1.2076997	0.0015823
C	0.6499330	1.2018503	0.0008783
N	0.0232237	0.0000646	0.0001893
H	3.8190264	-0.0005513	0.0021329
H	2.5640737	-2.1569400	0.0011783
H	2.5647794	2.1562482	0.0018236
C	-0.1974793	-2.4359127	-0.0008445
H	0.0227797	-3.0365415	-0.8892174
H	0.0325280	-3.0447294	0.8793363
H	-1.2609366	-2.1834404	0.0056417
C	-0.1966959	2.4361126	-0.0005205
H	-1.2602209	2.1839884	0.0082550
H	0.0351837	3.0464070	0.8781257
H	0.0220580	3.0350871	-0.8904017
H	-1.0335362	0.0002294	0.0004073
I	-3.4189760	0.0006550	-0.0013049

LutH$^+$: N-protonated 2,6-lutidine
18
Energy = -327.5641472182

C	1.2082953	-0.2231259	-0.0004049
C	1.2098840	1.1650835	-0.0001935
C	-0.0000980	1.8566067	-0.0002082
C	-1.2100224	1.1649811	-0.0000247
C	-1.2083078	-0.2232298	-0.0000901
N	0.0000202	-0.8446362	-0.0004350
H	-0.0001414	2.9416855	-0.0004962
H	2.1567799	1.6919262	0.0000902
H	-2.1569672	1.6917423	0.0003640
C	2.4372873	-1.0734474	-0.0001050
H	3.0454536	-0.8510379	-0.8823461
H	3.0428496	-0.8540970	0.8847137
H	2.1904720	-2.1376680	-0.0022882
C	-2.4372141	-1.0736782	0.0004479
H	-2.1902870	-2.1378744	0.0020942
H	-3.0445880	-0.8515963	0.8833185
H	-3.0436098	-0.8541397	-0.8837521
H	0.0000698	-1.8633725	-0.0007814

LutSiMe$_3^+$: silylium binding to Lut
30
Energy = -736.3614736019

Si	0.2562103	0.8609364	4.7916675
C	0.5507837	2.6854870	5.0905912
C	1.6602234	-0.0025825	3.9219606
C	-1.3703926	0.7965884	3.8669104
H	0.2374754	3.1817051	4.1634300
H	-0.0763799	3.0857265	5.8943752
H	1.5888966	2.9619048	5.2786429
H	1.4472343	-1.0625111	3.7487216
H	1.8166512	0.4725980	2.9459475
H	2.5974536	0.0685365	4.4836202
H	-2.2323237	0.9634141	4.5217504
H	-1.3343188	1.6389365	3.1646890
H	-1.5364024	-0.1111174	3.2855235
C	0.7515342	0.4124358	7.5751512
C	0.4400443	-0.0832273	8.8352632
C	-0.5242413	-1.0697997	8.9873757
C	-1.1054156	-1.6060056	7.8467527
C	-0.7886001	-1.1053221	6.5899687
N	0.0763618	-0.0415566	6.4644983
H	-0.7793220	-1.4495547	9.9711705
H	0.9846312	0.3038435	9.6888785
H	-1.7980009	-2.4375628	7.9091484
C	1.8773839	1.3947501	7.4461728
H	2.5665337	1.2335029	8.2777593
H	2.4319506	1.2662023	6.5149636
H	1.5243908	2.4277612	7.5045598
C	-1.3522203	-1.7887453	5.3798461
H	-2.2958502	-1.3380630	5.0610787
H	-0.6569215	-1.7775278	4.5386263
H	-1.5544367	-2.8297218	5.6403425

Lut : 2,6-lutidine
17
Energy = -327.1230377033

N	-0.0000129	0.9540216	-0.0001969
C	-1.1620199	0.2711684	-0.0001191
C	-1.1998407	-1.1278645	-0.0002487
C	-0.0000009	-1.8339237	-0.0000068
C	1.1998293	-1.1278487	0.0002347
C	1.1620085	0.2711889	-0.0000814
H	0.0000010	-2.9205868	-0.0000970
H	-2.1532889	-1.6486784	-0.0008041
H	2.1532786	-1.6486602	0.0009639
C	-2.4295466	1.0871858	-0.0001735
H	-3.0411209	0.8608796	-0.8813723
H	-3.0386415	0.8646235	0.8837135
H	-2.1841711	2.1509836	-0.0026978
C	2.4295473	1.0872012	0.0002156
H	2.1842150	2.1510123	0.0026629
H	3.0409412	0.8608851	0.8815427
H	3.0388225	0.8645832	-0.8835348

O(SiMe$_3$)$_2$: by-product
27
Energy = -894.1373340381

O	-0.0001432	0.0216192	-0.0218264
Si	-1.6398256	0.0057545	-0.0047195
Si	1.6395352	0.0047135	-0.0060280
C	-2.2574285	1.7423417	-0.3660105
C	-2.2401669	-1.1890302	-1.3238337
C	-2.2159690	-0.5525169	1.6937153
C	2.2578337	1.7425659	-0.3599444
C	2.2166316	-0.5616417	1.6894026
C	2.2382593	-1.1844500	-1.3309522
H	-3.3538740	1.7837060	-0.3657722
H	-1.8944568	2.4523781	0.3870965
H	-1.9085159	2.0860510	-1.3474056
H	-3.3360911	-1.2333548	-1.3527877
H	-1.8899637	-0.8843715	-2.3175783
H	-1.8679552	-2.2029520	-1.1328066
H	-1.8424116	-1.5577719	1.9237491
H	-1.8524914	0.1274550	2.4738056
H	-3.3111529	-0.5789589	1.7546905
H	1.9072556	2.0914644	-1.3389056
H	1.8968734	2.4490772	0.3974339
H	3.3543099	1.7830996	-0.3617507
H	1.8433975	-1.5681208	1.9145539

H	3.3118487	-0.5880848	1.7497403
H	1.8534015	0.1143227	2.4730790
H	3.3341217	-1.2301716	-1.3600278
H	1.8646882	-2.1988160	-1.1450217
H	1.8885303	-0.8743604	-2.3231857

$SiMe_3Cl$: chlorosilane
14
Energy = -869.6776581895

Si	0.0000132	-0.0000201	-0.3454961
C	-0.0000569	1.7902628	-0.8874499
C	1.5504196	-0.8951041	-0.8874130
C	-1.5503545	-0.8951853	-0.8874092
H	-0.0008051	1.8501256	-1.9832543
H	-0.8876821	2.3146844	-0.5171584
H	0.8882092	2.3144469	-0.5183675
H	1.6028186	-0.9242624	-1.9832045
H	2.4483095	-0.3887080	-0.5168554
H	1.5600019	-1.9264953	-0.5184664
H	-1.5605507	-1.9261216	-0.5172199
H	-2.4483300	-0.3880333	-0.5180971
H	-1.6020030	-0.9256740	-1.9831991
Cl	0.0000104	0.0000855	1.7637789

$SiMe_3H$: silane
14
Energy = -409.9782748062

Si	2.5155841	-2.0176785	-0.4461132
C	2.0518300	-3.8428785	-0.4674555
C	4.0774087	-1.7221617	-1.4561692
C	2.7512352	-1.4178312	1.3234897
H	2.8478845	-4.4510034	-0.0200322
H	1.1312779	-4.0244089	0.0998341
H	1.8923478	-4.1992455	-1.4920524
H	4.9233675	-2.2793591	-1.0347858
H	3.9450321	-2.0471589	-2.4949495
H	4.3470349	-0.6594321	-1.4671107
H	3.0003937	-0.3503856	1.3503049
H	1.8412670	-1.5665861	1.9168351
H	3.5655835	-1.9656009	1.8138614
H	1.3998424	-1.2398441	-1.0635129

SimI : iodosilane
14
Energy = -707.0311227089

Si	0.0000112	-0.0000277	-1.2676981
C	-0.0000359	1.7924136	-1.8095310
C	1.5522687	-0.8961914	-1.8095264
C	-1.5522192	-0.8962410	-1.8095356
H	-0.0004442	1.8427542	-2.9063970
H	-0.8878276	2.3173857	-1.4422690
H	0.8881179	2.3172291	-1.4429267
H	1.5961777	-0.9210077	-2.9063758
H	2.4507175	-0.3898346	-1.4421249
H	1.5625126	-1.9277614	-1.4429503
H	-1.5627902	-1.9276021	-1.4423854
H	-2.4506884	-0.3894981	-1.4427162
H	-1.5957799	-0.9216879	-2.9063835
I	-0.0000204	0.0000673	1.2403782

TS1 : TS for H_2-cleavage with Lut.$B(C_6F_5)_3$
53
Energy = -2537.822947856

B	-0.0332434	0.3977256	-0.0392261
C	-0.2806106	0.8396335	1.4862393
C	0.2484169	1.6472981	-1.0161299
C	-0.8645133	-0.8495711	-0.6155007
C	-1.5111964	0.7533505	2.1384871
C	0.7497422	1.4119857	2.2332076
C	1.3790649	1.8116379	-1.8140146
C	-0.6879245	2.6798562	-1.0874027
C	-1.0713490	-1.9992318	0.1570678
C	-1.4175494	-0.8962323	-1.9002364
C	-1.7059716	1.1827147	3.4481779
F	-2.5884461	0.2462505	1.4990087
C	0.6066188	1.8389528	3.5478579
F	1.9766102	1.5578711	1.6647728
C	1.5864057	2.9225185	-2.6261549
F	2.3474085	0.8590027	-1.8210177
C	-0.5257988	3.8044973	-1.8882208
F	-1.8351345	2.5881539	-0.3734928
C	-1.7610368	-3.1179205	-0.2925717
F	-0.5865416	-2.0582261	1.4171215
C	-2.1295133	-1.9921850	-2.3836535
F	-1.2849495	0.1403011	-2.7549429
C	-0.6386490	1.7238882	4.1597154
F	-2.9124679	1.0806566	4.0323804
F	1.6440151	2.3615025	4.2249584
C	0.6245966	3.9272299	-2.6633022
F	2.7000640	3.0329739	-3.3714174
F	-1.4646287	4.7657867	-1.9268089
C	-2.2991794	-3.1124957	-1.5768525
F	-1.9158488	-4.1956676	0.4944296
F	-2.6440246	-1.9801692	-3.6249665
F	-0.8095821	2.1369931	5.4235568
F	0.8025190	5.0045191	-3.4418422
F	-2.9740531	-4.1762527	-2.0304890
H	1.4548327	0.1248848	0.0952623
C	2.7017022	-2.8348648	1.2751055
C	3.3037843	-4.0838261	1.4512022
C	3.3907271	-4.9522340	0.3666974
C	2.8824846	-4.5491153	-0.8647701

C	2.2881592	-3.2885439	-0.9742579
N	2.1990244	-2.4576349	0.0825962
H	3.8547400	-5.9280317	0.4783314
H	3.6989871	-4.3613934	2.4233480
H	2.9425356	-5.1964812	-1.7340355
C	2.5985152	-1.8474644	2.4066816
H	1.5498048	-1.6406617	2.6426935
H	3.0876497	-2.2297584	3.3053256
H	3.0710744	-0.8995296	2.1294247
C	1.7174418	-2.8010130	-2.2780447
H	2.0889276	-1.7983935	-2.5098552
H	1.9757914	-3.4751093	-3.0976803
H	0.6256616	-2.7400973	-2.2160020
H	1.2331036	-0.6305934	0.0215199

TS2 : TS for hydride transfer from **A** to CO_2
56
Energy = -2726.548000605

B	-0.7059682	0.1847510	0.2489573
C	-1.2300275	-0.1544528	1.7386240
C	-1.8402461	0.3874654	-0.8720625
C	0.6446069	-0.6171050	-0.1288862
C	-1.2385198	-1.4617074	2.2297776
C	-1.7137448	0.8055886	2.6260042
C	-1.5544316	1.0467829	-2.0705367
C	-3.1759464	-0.0020141	-0.7351066
C	1.7076322	-0.6450528	0.7819901
C	0.8611093	-1.3646504	-1.2894849
C	-1.6729030	-1.8033493	3.5058852
F	-0.8358467	-2.4880265	1.4363322
C	-2.1537861	0.5121346	3.9138508
F	-1.7679218	2.1057769	2.2513035
C	-2.4909776	1.3235763	-3.0561737
F	-0.2807893	1.4480720	-2.3218503
C	-4.1507616	0.2466147	-1.6995698
F	-3.5956927	-0.6783745	0.3606196
C	2.8867379	-1.3529624	0.5808542
F	1.6177408	0.0432637	1.9472318
C	2.0368975	-2.0695208	-1.5427615
F	-0.0969436	-1.4651158	-2.2385086
C	-2.1323446	-0.8049874	4.3591897
F	-1.6625253	-3.0860459	3.9210922
F	-2.6000585	1.4876479	4.7301323
C	-3.8092315	0.9194731	-2.8667219
F	-2.1413389	1.9680334	-4.1866676
F	-5.4209217	-0.1622901	-1.5136722
C	3.0601017	-2.0651547	-0.6013411
F	3.8676784	-1.3431003	1.5074779
F	2.1942418	-2.7558412	-2.6916800
F	-2.5553020	-1.1117341	5.5978257
F	-4.7371476	1.1688175	-3.8049000
F	4.2029443	-2.7329870	-0.8325754
H	-0.2176246	1.4703627	0.3424854
C	4.7488883	1.6410660	0.5775188
C	5.9773973	1.0422864	0.3373240
C	6.2427575	0.4868327	-0.9135489
C	5.2874869	0.5475136	-1.9240700
C	4.0647385	1.1598163	-1.6766156
N	3.8472343	1.6676526	-0.4388384
H	7.1986873	0.0087351	-1.1005707
H	6.7115146	1.0075453	1.1331687
H	5.4785373	0.1269585	-2.9042255
C	4.3403908	2.2377207	1.8856332
H	5.2083283	2.3324883	2.5387280
H	3.8839476	3.2206841	1.7401197
H	3.5988211	1.5981566	2.3761024
C	2.9858972	1.3181044	-2.6985504
H	3.2574951	2.1191516	-3.3954167
H	2.8728459	0.3995758	-3.2786473
H	2.0331058	1.5713098	-2.2356517
H	2.9113769	2.0837880	-0.2384588
O	1.4471640	2.7418778	0.2533669
C	0.2323443	2.7574105	0.1211068
O	-0.7050666	3.4637174	-0.1214683

TS3⁻ : silylium transfer from $SiMe_3I$ to anion **B⁻**
52
Energy = -3106.008417658

O	0.4006200	0.1356640	-1.1696549
C	-0.8600676	0.1749337	-0.8602512
H	-1.0959366	0.1402798	0.2144244
O	-1.7566897	0.2417380	-1.7043038
Si	-4.1463483	0.1355169	-0.8856237
C	-3.6072514	-1.4980924	-0.1218738
C	-3.7008452	1.7516968	-0.0331128
C	-4.4809962	0.1663053	-2.7291880
H	-2.6316620	-1.8353977	-0.4781308
H	-3.5751740	-1.4102469	0.9701433
H	-4.3495073	-2.2652687	-0.3647743
H	-2.7236105	2.1346406	-0.3337966
H	-4.4607240	2.5018571	-0.2758258
H	-3.7117236	1.6158698	1.0542113
H	-3.5469873	0.1805568	-3.2959018
H	-5.0629629	-0.7164781	-3.0148132
H	-5.0717672	1.0523170	-2.9854754
I	-6.6449791	0.0473315	-0.0563042
B	1.5506922	0.0203472	-0.1499625
C	2.1655681	1.5337531	0.0649529
C	0.9923733	-0.6550340	1.2438530
C	2.6296742	-0.9361837	-0.9446421
C	3.5303754	1.8153532	0.1521058
C	1.3478555	2.6608278	0.1658319

C	0.2527286	-1.8419218	1.1963857
C	1.1813840	-0.1547968	2.5323888
C	2.9870143	-0.6128595	-2.2576545
C	3.2556733	-2.0772077	-0.4451761
C	4.0529914	3.0998488	0.2806677
C	1.8205622	3.9632973	0.2917428
C	-0.2876304	-2.4762969	2.3072128
C	0.6673530	-0.7600160	3.6796267
C	3.8590706	-1.3692118	-3.0343064
C	4.1342262	-2.8657917	-1.1857247
C	3.1911812	4.1876696	0.3443491
C	-0.0761322	-1.9275378	3.5688014
C	4.4366679	-2.5127717	-2.4943939
F	4.4490888	0.8145379	0.1456873
F	-0.0064186	2.5278097	0.1870930
F	0.0308805	-2.4435129	-0.0017661
F	1.9018435	0.9736403	2.7510986
F	2.5069292	0.5144045	-2.8368511
F	3.0586230	-2.4821172	0.8371515
F	5.3865717	3.2994268	0.3531496
F	0.9679380	5.0057882	0.3805712
F	-1.0007666	-3.6138259	2.1819949
F	0.8861269	-0.2221396	4.8974859
F	4.1626738	-1.0004249	-4.2977342
F	4.7004383	-3.9654944	-0.6424933
F	3.6750449	5.4378129	0.4697354
F	-0.5825455	-2.5216183	4.6639501
F	5.2837969	-3.2616120	-3.2265457

TS4 : silylium transfer from $SiMe_3I$ to **D**
31
Energy = -1305.732668379

O	3.6755789	-1.2069881	0.3281090
Si	4.6799199	0.2022751	0.0055598
C	2.3711537	-1.1873706	0.2836414
C	4.3804280	0.7116576	-1.7633315
C	4.2316279	1.5138019	1.2537637
C	6.3841675	-0.4978338	0.2783868
H	1.9085238	-2.1503067	0.5274912
O	1.7115553	-0.1830451	-0.0049262
H	5.0846753	1.5053502	-2.0416581
H	4.5376290	-0.1293346	-2.4481072
H	3.3648964	1.0927871	-1.9065371
H	4.9402802	2.3478606	1.1785898
H	3.2247380	1.9067277	1.0848439
H	4.2862880	1.1212919	2.2755195
H	6.5029659	-0.8628582	1.3046580
H	6.5872546	-1.3273103	-0.4080659
H	7.1414384	0.2762905	0.1058919
Si	-0.4603308	-0.0578033	-0.0741055
C	-0.3643657	-1.3769439	-1.4192793
C	-0.4634723	-0.5217907	1.7540721
C	-0.2268745	1.7410175	-0.5563115
H	0.5561812	-1.2837663	-2.0051857
H	-0.3878660	-2.3772850	-0.9697083
H	-1.2194345	-1.2992813	-2.0938623
H	0.5189779	-0.4191864	2.2256116
H	-1.1730588	0.1172524	2.2867391
H	-0.8059912	-1.5559460	1.8747129
H	-0.6855771	1.9214337	-1.5340451
H	-0.7360941	2.3859934	0.1669588
H	0.8297152	2.0211077	-0.6026282
I	-3.1998053	0.0828337	-0.1488637

TS5 : hydride transfer from $\mathbf{A}^-$ to $\mathbf{E}^+$
65
Energy = -3218.237915100

O	2.3193961	-1.2232243	-1.5178069
Si	2.2442675	-2.9179341	-1.9205853
C	1.6026990	-0.2268576	-1.9583979
C	1.0536229	-3.7504590	-0.7612903
C	1.7405350	-3.0302929	-3.7129872
C	4.0042811	-3.4440875	-1.6180193
H	0.8368708	-0.3973694	-2.7106468
O	2.0885618	0.9732022	-1.9157375
H	1.0388293	-4.8259954	-0.9767963
H	1.3608902	-3.6234715	0.2815229
H	0.0352521	-3.3699573	-0.8753549
H	1.8125398	-4.0748674	-4.0391076
H	0.7086361	-2.7057756	-3.8807246
H	2.4016722	-2.4362228	-4.3545395
H	4.6993927	-2.9332169	-2.2928804
H	4.3062453	-3.2302196	-0.5866134
H	4.1063383	-4.5236298	-1.7803700
Si	3.6114964	1.6938861	-1.4614825
C	4.1716566	2.4231700	-3.0854107
C	3.1750160	3.0093921	-0.2186611
C	4.8214508	0.4319823	-0.8175124
H	4.3562853	1.6420972	-3.8316948
H	3.4223814	3.1134939	-3.4879432
H	5.1053316	2.9811516	-2.9447152
H	2.7345618	2.5870832	0.6882073
H	4.0843106	3.5544355	0.0637890
H	2.4703143	3.7317952	-0.6421747
H	5.7552048	0.9531945	-0.5693477
H	4.4590342	-0.0678565	0.0841294
H	5.0535048	-0.3274960	-1.5703930
B	-0.4771037	0.0167940	0.0613380
C	0.0925492	-0.9531122	1.2258180
C	-0.3786147	1.6159996	0.3136170
C	-1.9360160	-0.3655026	-0.5398095
C	-0.5915106	-2.0112730	1.8282568

C	1.4166881	-0.8142353	1.6487110
C	-0.5113918	2.4782158	-0.7774852
C	-0.2076875	2.2534210	1.5426367
C	-3.1057865	0.0571558	0.0952250
C	-2.1439350	-1.0895043	-1.7084273
C	-0.0159664	-2.8631908	2.7702432
F	-1.8828197	-2.2763226	1.5149951
C	2.0388051	-1.6415874	2.5743025
F	2.1688810	0.1984241	1.1448645
C	-0.4630661	3.8643814	-0.6819636
F	-0.7060919	1.9625711	-2.0190776
C	-0.1535219	3.6376083	1.6879704
F	-0.1056449	1.5312751	2.6850536
C	-4.3841728	-0.2055707	-0.3855078
F	-3.0236741	0.7385775	1.2653115
C	-3.3999283	-1.3799675	-2.2336641
F	-1.0779929	-1.5691820	-2.4156033
C	1.3108242	-2.6824608	3.1447036
F	-0.7290967	-3.8687959	3.3138800
F	3.3268747	-1.4500715	2.9248269
C	-0.2794230	4.4508078	0.5665249
F	-0.5807393	4.6419579	-1.7778122
F	0.0163386	4.1986456	2.9021189
C	-4.5334571	-0.9303477	-1.5653079
F	-5.4768906	0.2229687	0.2770743
F	-3.5280486	-2.0856500	-3.3758806
F	1.8832513	-3.5004042	4.0438263
F	-0.2189084	5.7885634	0.6864407
F	-5.7592233	-1.1957739	-2.0498568
H	0.3673729	-0.1602364	-0.8819147

TS6 : silylium transfer from $SiMe_3I$ to **E**
45
Energy = -1715.739072524

O	0.9435513	-0.2780725	-0.3171676
C	1.8657774	-0.2618767	0.8042982
H	1.6217179	-1.1217158	1.4307855
O	3.1545885	-0.3642168	0.2927957
Si	4.5660671	0.0133743	1.1510223
C	4.5313945	1.8280879	1.6155921
H	3.6602034	2.0716096	2.2354733
H	5.4255862	2.0873414	2.1956718
H	4.5074990	2.4704455	0.7286187
C	4.6333922	-1.0598449	2.6845017
H	5.5672570	-0.8865292	3.2331652
H	3.8056822	-0.8389317	3.3695659
H	4.5872079	-2.1244194	2.4270833
C	5.9319872	-0.3858187	-0.0567629
H	5.9136695	-1.4449792	-0.3371971
H	5.8435962	0.2116593	-0.9712423
H	6.9110061	-0.1722524	0.3886728
H	1.7141540	0.6788800	1.3505526
Si	1.6251838	0.0808360	-1.8948888
C	0.1847210	0.3509955	-3.0601710
C	2.5922610	-1.3939688	-2.5143281
C	2.6037531	1.6802208	-1.8055318
H	0.6395852	0.6556884	-4.0131781
H	-0.4014714	-0.5497766	-3.2538793
H	-0.4916316	1.1529735	-2.7551431
H	3.5304080	-1.5342309	-1.9736396
H	1.9982062	-2.3108103	-2.4193546
H	2.8167313	-1.2569469	-3.5799270
H	3.6749811	1.4980171	-1.6869951
H	2.4523307	2.2590602	-2.7243096
H	2.2744873	2.3049962	-0.9673114
C	-0.8287703	-0.7754124	1.9300195
C	-1.2077703	1.5700967	-0.2148930
C	-1.3945332	-1.7002584	-1.0576842
H	-0.3540202	-1.7606949	1.9955352
H	-0.2136612	-0.0491185	2.4715726
H	-1.7960588	-0.8350152	2.4319400
H	-1.8714406	1.7804913	-1.0574756
H	-1.6422343	2.0576496	0.6638290
H	-0.2252864	2.0117505	-0.4119983
H	-1.9175810	-2.4876327	-0.5051035
H	-2.0383562	-1.4087901	-1.8908095
H	-0.4563996	-2.1103606	-1.4441886
Si	-1.1270123	-0.2768523	0.1394870
I	-3.9833742	-0.2028419	0.5121304

TS7 : hydride transfer from $\mathbf{A}^{-}$ to $\mathbf{F}^{+}$
79
Energy = -3628.251465697

O	3.6519642	-0.6190410	0.0212912
C	2.0809969	0.3317471	0.3175016
H	1.8401693	-0.1576788	1.2542954
O	2.3862487	1.5925438	0.3076797
Si	2.5553134	2.6869636	1.6568814
C	2.4568310	4.3376270	0.8070512
H	3.2382427	4.4337092	0.0447894
H	1.4865281	4.4755022	0.3192352
H	2.5922385	5.1486533	1.5319903
C	1.1286908	2.3395163	2.8023790
H	1.1378778	3.0634676	3.6262237
H	0.1732168	2.4331813	2.2754575
H	1.1723073	1.3380650	3.2430877
C	4.2246105	2.3732363	2.4312034
H	4.3862498	3.0880026	3.2473124
H	5.0349154	2.5041611	1.7068481
H	4.3023238	1.3681676	2.8576220
H	1.6062538	-0.0383404	-0.5816536
Si	4.3984886	-0.1990505	-1.4940892

C	5.2550835	-1.7192866	-2.1613863
C	5.5954250	1.1924056	-1.1573834
C	3.0378933	0.3190831	-2.6630759
H	5.7494884	-1.4373457	-3.1000498
H	6.0249587	-2.1231958	-1.4971405
H	4.5386118	-2.5149644	-2.3904853
H	6.3346451	0.9212222	-0.3963338
H	5.0695820	2.0928871	-0.8234319
H	6.1380514	1.4448207	-2.0764556
H	2.5176450	1.2277933	-2.3465158
H	3.4962211	0.5249588	-3.6386561
H	2.2973230	-0.4745711	-2.8141662
C	3.1252819	-1.5701416	2.7284074
C	3.8491700	-3.5074508	0.4831166
C	5.9588311	-1.4759643	1.5460243
H	3.1444279	-0.5488920	3.1223012
H	2.0814890	-1.8758343	2.5992535
H	3.5571246	-2.2163605	3.5036804
H	4.5333829	-3.7646095	-0.3299818
H	3.9867864	-4.2479471	1.2813464
H	2.8219175	-3.6002310	0.1156541
H	6.3140885	-2.2054467	2.2846137
H	6.5949480	-1.5712735	0.6602069
H	6.1062418	-0.4755697	1.9679488
Si	4.1546432	-1.8071490	1.1850989
B	-1.2244451	0.0749581	0.2430309
C	-1.7367283	1.6095463	0.0230179
C	-0.9552775	-0.7791294	-1.1323261
C	-2.1240575	-0.8527111	1.2363147
C	-2.7772423	2.2416340	0.7044435
C	-1.0110378	2.4611982	-0.8146097
C	-0.0946020	-1.8777910	-1.0859272
C	-1.5019875	-0.5365337	-2.3924572
C	-3.3760944	-1.3433226	0.8682356
C	-1.6715408	-1.2852373	2.4801916
C	-3.0835671	3.5965229	0.5706975
F	-3.5639784	1.5523931	1.5710274
C	-1.2743355	3.8149008	-0.9816685
F	0.0373430	1.9654848	-1.5285017
C	0.2454441	-2.6580611	-2.1861525
F	0.4802869	-2.2336614	0.1012828
C	-1.1972024	-1.2895516	-3.5251389
F	-2.3799212	0.4789529	-2.5767082
C	-4.1397552	-2.1929549	1.6602688
F	-3.9117226	-0.9711659	-0.3231646
C	-2.3956930	-2.1390099	3.3098760
F	-0.4602224	-0.8823957	2.9554114
C	-2.3270946	4.3933606	-0.2790769
F	-4.1060492	4.1434333	1.2622829
F	-0.5202467	4.5755070	-1.8064587
C	-0.3112997	-2.3573226	-3.4250993
F	1.1071880	-3.6936480	-2.0696677
F	-1.7518371	-0.9974392	-4.7195765
C	-3.6420173	-2.5958573	2.8966461
F	-5.3522132	-2.6278371	1.2546785
F	-1.9040297	-2.5295734	4.5066667
F	-2.6033785	5.7042643	-0.4189393
F	-0.0003360	-3.0932294	-4.5087744
F	-4.3609666	-3.4200349	3.6836101
H	-0.1419769	0.2064010	0.7784779

TS8 : silylium transfer from $SiMe_3I$ to **F**
32
Energy = -1231.643751783

O	2.1490428	-0.1212971	0.7420031
C	2.7233864	0.0481158	2.0800539
H	2.3493419	-0.7492335	2.7181950
H	3.8104807	-0.0221796	2.0109009
H	2.4405698	1.0273767	2.4693810
Si	3.3310647	0.0066844	-0.5380486
C	2.4624482	0.0572878	-2.1862303
C	4.4191815	-1.5039822	-0.3853736
C	4.2761100	1.5971960	-0.2601144
H	3.2254587	0.3224683	-2.9303679
H	2.0372257	-0.9058526	-2.4773787
H	1.6793003	0.8183672	-2.2381804
H	4.9533440	-1.5460931	0.5699766
H	3.8287284	-2.4215447	-0.4880864
H	5.1707598	-1.4972388	-1.1843899
H	4.8328712	1.6121675	0.6822322
H	5.0023747	1.7208373	-1.0732417
H	3.6090164	2.4663490	-0.2784316
C	-0.2223303	-0.3662427	2.3246953
C	0.1989382	1.7300992	-0.0937057
C	0.1374116	-1.5803481	-0.5859691
H	0.2543085	-1.2934712	2.6621598
H	0.1203393	0.4557171	2.9619114
H	-1.3000494	-0.4710274	2.4590503
H	-0.1508489	1.8065618	-1.1268259
H	-0.4823360	2.3287130	0.5184372
H	1.2039214	2.1606333	-0.0211757
H	-0.6159234	-2.2698137	-0.1927812
H	-0.1473740	-1.3425251	-1.6136513
H	1.1073338	-2.0897229	-0.5846402
Si	0.1283625	-0.0522584	0.5065396
I	-2.7773043	0.1137370	-0.0920847

TS9^{+} : iodide transfer from LutHI to **G**$^{+}$
50
Energy = -1559.220457504

O	2.9947838	-0.1472545	-0.1146678
Si	4.0628017	-0.4404914	-1.4555878

Si	3.0247254	-0.6920401	1.5301372
C	3.9491417	-2.2643100	-1.8396201
C	5.7756857	0.1102408	-0.9579660
C	3.4310331	0.5994515	-2.8711116
C	1.6823077	-1.9876124	1.6749223
C	2.6947529	0.8129401	2.5830884
C	4.7013141	-1.4194806	1.9010329
H	4.5910077	-2.5070547	-2.6950260
H	4.2720553	-2.8855646	-0.9971070
H	2.9241595	-2.5507446	-2.1028635
H	6.2939903	-0.6162589	-0.3264730
H	5.7464705	1.0715099	-0.4327606
H	6.3748204	0.2484894	-1.8667784
H	3.4442991	1.6694194	-2.6352048
H	4.1020656	0.4458905	-3.7256535
H	2.4221836	0.3268549	-3.1976957
H	1.8415121	-2.8040776	0.9616219
H	1.6916978	-2.4183360	2.6829403
H	0.6816019	-1.5742700	1.5069189
H	3.4028917	1.6142533	2.3451587
H	1.6810489	1.2120615	2.4689921
H	2.8260989	0.5527879	3.6402066
H	4.6526013	-1.8683621	2.9016250
H	4.9919504	-2.2111823	1.2033748
H	5.4879094	-0.6594164	1.9176436
C	1.3352112	0.4771287	-0.5560634
H	0.9307825	0.5327415	0.4409418
H	1.0300713	-0.3448161	-1.1819571
H	1.6489103	1.3980995	-1.0171823
I	-1.2186483	1.4285730	-1.2133027
C	-4.6279281	-0.0863881	0.8922308
C	-5.6187625	-0.9114395	1.4083827
C	-5.5059261	-2.2942149	1.2720822
C	-4.4022403	-2.8503609	0.6274841
C	-3.4123605	-2.0173083	0.1229847
N	-3.5719587	-0.6790312	0.2780874
H	-6.2824965	-2.9409109	1.6674741
H	-6.4722471	-0.4641488	1.9039533
H	-4.3015167	-3.9232265	0.5104396
C	-4.6449869	1.4067136	0.9586676
H	-5.5515892	1.7478979	1.4590940
H	-3.7722551	1.7785990	1.5057607
H	-4.6062639	1.8353894	-0.0481124
C	-2.1705350	-2.4944540	-0.5614991
H	-1.9098636	-1.8389705	-1.3971635
H	-1.3298548	-2.4896417	0.1428393
H	-2.3080335	-3.5145011	-0.9213271
H	-2.8392698	-0.0544850	-0.1319522

TS10 : TS for hydride transfer, to $O(SiMe_3)_2$
66
Energy = -3144.133521144

O	3.7066274	0.0347915	1.0942857
C	1.9543350	0.2416419	0.6879423
H	1.7185449	-0.8074861	0.6699566
H	1.6779634	0.8086276	1.5626416
H	2.0878335	0.7562109	-0.2501275
Si	4.6420959	1.4916416	1.2849579
C	6.1394035	1.3554745	0.1768498
C	5.0626541	1.6347725	3.0961410
C	3.5686386	2.9131770	0.7271270
H	6.7245712	2.2795897	0.2656439
H	6.8013829	0.5231567	0.4328291
H	5.8435994	1.2519195	-0.8730838
H	5.7394628	0.8435507	3.4320623
H	4.1571233	1.5966528	3.7126770
H	5.5533585	2.5978102	3.2832992
H	2.6616305	3.0415622	1.3254058
H	4.1608056	3.8310848	0.8338079
H	3.2775430	2.8381034	-0.3260298
C	2.8672992	-2.6997684	1.6556942
C	4.6378740	-1.9642938	-0.7778420
C	5.7351214	-1.7802756	2.1369069
H	2.4244563	-2.2996729	2.5744907
H	2.0683423	-2.8712681	0.9265440
H	3.2944788	-3.6811388	1.8976956
H	5.4381898	-1.3198003	-1.1558001
H	4.9609310	-3.0057613	-0.8958184
H	3.7519512	-1.8193972	-1.4065511
H	6.0885774	-2.8175622	2.0744849
H	6.5740640	-1.1332926	1.8661033
H	5.4654875	-1.5857722	3.1803816
Si	4.2525324	-1.6187355	1.0133304
B	-0.8730028	0.1842908	-0.1440019
C	-1.7055361	1.4374191	0.4624100
C	-0.6601474	0.1898553	-1.7578053
C	-1.3025053	-1.2613928	0.4636380
C	-3.0088215	1.3755139	0.9541164
C	-1.1099639	2.6956932	0.5540541
C	0.2828594	-0.6605845	-2.3387044
C	-1.3257697	1.0110954	-2.6690955
C	-2.0318186	-2.2321057	-0.2235153
C	-0.9236239	-1.6299365	1.7545432
C	-3.6745087	2.4676019	1.5070124
F	-3.7126179	0.2171118	0.8910809
C	-1.7304317	3.8144623	1.0971481
F	0.1563772	2.8744184	0.0843294
C	0.5738343	-0.7027322	-3.6965792
F	0.9812016	-1.5299170	-1.5501377
C	-1.0709719	1.0073071	-4.0399164
F	-2.2935174	1.8653692	-2.2539910
C	-2.3416411	-3.4820767	0.3062802

F	-2.4980691	-1.9801564	-1.4721326
C	-1.2029599	-2.8667075	2.3267366
F	-0.2357599	-0.7490067	2.5354161
C	-3.0299157	3.6976740	1.5816805
F	-4.9372791	2.3482224	1.9654857
F	-1.0945832	5.0031367	1.1599564
C	-0.1117069	0.1462928	-4.5602040
F	1.5053287	-1.5490230	-4.1833747
F	-1.7473719	1.8280204	-4.8685336
C	-1.9192425	-3.8056481	1.5918520
F	-3.0489503	-4.3810401	-0.4077216
F	-0.7892666	-3.1649518	3.5762872
F	-3.6555019	4.7636141	2.1132162
F	0.1495163	0.1313953	-5.8792634
F	-2.2052741	-5.0091085	2.1205917
H	0.2832033	0.3732110	0.3039577

Computational References

1 *TURBOMOLE V7.4*, **2019**, a development of University of Karlsruhe and Forschungszentrum Karlsruhe GmbH, 1989-2007, TURBOMOLE GmbH, since 2007; available from http://www.turbomole.com.

2 J. Tao, J. P. Perdew, V. N. Staroverov and G. E. Scuseria, *Phys. Rev. Lett.*, 2003, **91**, 146401.

3 S. Grimme, J. Antony, S. Ehrlich and H. Krieg, *J. Chem. Phys.*, 2010, **132**, 154104-154119.

4 S. Grimme, S. Ehrlich and L. Goerigk, *J. Comput. Chem.*, 2011, **32**, 1456-1465.

5 F. Weigend, M. Häser, H. Patzelt and R. Ahlrichs, *Chem. Phys. Lett.*, 1998, **294**, 143-152.

6 F. Weigend and R. Ahlrichs, *Phys. Chem. Chem. Phys.*, 2005, **7**, 3297-3305.

7 A. Klamt and G. Schüürmann, *J. Chem. Soc., Perkin Trans. 2*, 1993, 799-805.

8 K. Eichkorn, F. Weigend, O. Treutler and R. Ahlrichs, *Theor. Chem. Acc.*, 1997, **97**, 119-124.

9 F. Weigend, *Phys. Chem. Chem. Phys.*, 2006, **8**, 1057-1065.

10 P. Deglmann, K. May, F. Furche and R. Ahlrichs, *Chem. Phys. Lett.*, 2004, **384**, 103-107.

11 S. Grimme, *Chem. - Eur. J.*, 2012, **18**, 9955-9964.

12 F. Eckert and A. Klamt, *AIChE J.*, 2002, **48**, 369-385.

13 F. Eckert and A. Klamt, *COSMOtherm*, Version C3.0, Release 16.01; COSMOlogic GmbH & Co. KG, Leverkusen, Germany 2015.

14 Y. Zhao and D. G. Truhlar, *J. Phys. Chem. A*, 2005, **109**, 5656-5667.

15 F. Weigend, F. Furche and R. Ahlrichs, *J. Chem. Phys.*, 2003, **119**, 12753-12762.

16 L. Goerigk, A. Hansen, C. Bauer, S. Ehrlich, A. Najibi, S. Grimme, *Phys. Chem. Chem. Phys.* **2017**, *19*, 32184-32215.
